# Bacterial Surfaces and Barriers: Biogenesis and Pathogen–Host Interactions

**DOI:** 10.1021/acs.chemrev.5c01068

**Published:** 2026-10-16

**Authors:** Jackson Bartholomew-Schoch, Juaneisha K. Finnie, Andreas Gahlmann, Linda Columbus

**Affiliations:** 2358 University of Virginia, Charlottesville, Virginia 22904, United States

## Abstract

The bacterial surface represents the primary interface between pathogens and hosts, yet its chemical and architectural complexity challenges conventional classification. This Review examines structural organization, molecular composition, and biogenesis of bacterial surface components across the *Gracilicutes* and *Terrabacteria* clades, linking mechanistic detail to pathogenic function. We describe lipid diversity, integral membrane protein biogenesis, and lipoprotein processing at the inner membrane and how these components support surface remodeling, secretion, and virulence. We then examine the peptidoglycan layer and its role in immune recognition and surface protein display, teichoic acids and their contributions to antimicrobial resistance and immune evasion, and S layers, whose crystalline arrays mediate adhesion and immune modulation. The outer membrane section covers lipopolysaccharide biosynthesis, outer membrane protein biogenesis and folding, lipoprotein trafficking, and contributions to pathogenicity, while the mycobacterial section addresses the mycolyl-arabinogalactan-peptidoglycan complex and virulence factors. We discuss bacterial capsule diversity, biosynthesis, and use as vaccine antigens, along with adhesive surface structures such as fimbriae, pili, and curli fibers and their roles in colonization and biofilm formation, before covering secretion systems types I through XI and their contributions to virulence. This Review provides a framework for understanding how bacterial surface diversity shapes infection and identifies chemical vulnerabilities for therapeutics.

## Graphical abstract

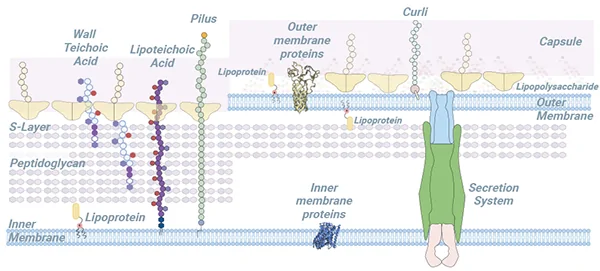

## 1 Introduction

Bacteria adapt to and evolve with their environment, with their secreted proteins and extracellular surface proteins evolving the fastest.^1^ Throughout this Review, the outer or extracellular surface refers to all structures that are exposed to or protrude into the extracellular environment, including the outermost membrane or layer, surface appendages, capsular polymers, and surface-associated secreted proteins. The bacterial extracellular surface controls how bacteria interact with other organisms, from communication within communities to direct contact between pathogens and hosts. Many bacteria dynamically remodel their surface properties in response to environmental changes, and this chemical diversity challenges our understanding of how membrane proteins fold, insert, and function. The outer surface of bacteria can be composed of lipoproteins, LPS, lipids, PG, and membrane proteins (MPs) with the composition varying among organisms. Each component can be modified to introduce additional chemical variability, enabling the adaptation of the outer bacterial surface to specific environmental niches and host interactions.

Bacteria are classified into two clades: *Gracilicutes* and *Terrabacteria*. The *Gracilicutes* clade contains only diderm bacteria, and the *Terrabacteria* clade contains both diderm (i.e., Gram-negative) and monoderm (i.e., Gram-positive) bacteria; both architectures can be found within the same phylum. The classic monoderm architecture, exemplified by model organisms such as *Bacillus subtilis* (*Terrabacteria*, *Firmicutes*), consists of an inner (cell) membrane (IM) and a PG layer. The IM is the first lipid bilayer barrier closest to the cytoplasm. The PG layer lies on the extracellular side of the IM and consists of alternating sugar residues (N-acetylglucosamine and N-acetylmuramic acid) cross-linked by short peptides into a rigid, meshlike layer. The classic diderm bacterial architecture, exemplified by *Escherichia coli* (*Gracilicutes*, *Proteobacteria*), adds a second lipid-containing OM beyond the IM and PG layer.

For many bacteria, membrane organization deviates considerably from the two classical monoderm and diderm architectures. For example, some monoderm bacteria possess additional outer layers yet lack the machinery to synthesize LPS or insert outer membrane proteins (OMPs), for these bacteria, a new classification within *Terrabacteria*, neoderm, was recently proposed. Some monoderm and diderm bacteria additionally possess an outermost proteinaceous surface (S) layer. Some bacteria have an IM and an S-layer but lack a PG layer, such as *Dehalococcoides mccartyi*, whereas cyanobacteria and *Atribacter laminatus* possess additional cytoplasmic membrane structures. Beyond the membrane layers themselves, many bacteria project capsular polymers, surface appendages, and multiprotein secretion machines that extend through or beyond the cell envelope to mediate direct contact with the environment and the host.

This review examines the structural organization, molecular composition, and biogenesis of bacterial surface layers and appendages (Figure 1), focusing on how each component contributes to pathogen–host interactions. The Review is organized from the innermost membrane to the outermost layers and appendages. We begin with the IM (Section 2) and cover lipid biosynthesis, the biogenesis of integral membrane proteins (IMPs), lipoprotein processing, and the roles of IM components in pathogenesis. We then discuss the PG layer (Section 3), including its biosynthesis, modification by sortase enzymes, and recognition by host immune receptors. Next, we describe teichoic acids (Section 4) and their roles in surface charge modulation, antimicrobial resistance, and host interactions. The S-layer (Section 5) is next, with sections describing secondary cell wall polysaccharide anchoring, S-protein biogenesis, and virulence functions. In Section 6 we examine the outer membrane (OM) of diderm bacteria (Section 6) in depth, encompassing LPS biosynthesis and immune recognition, OMP biogenesis and folding, LPS-OMP interactions, lipoprotein trafficking, and the contributions of these OM components to pathogenicity. We treat the mycobacterial cell envelope (Section 7) as a distinct architecture, discussing mycomembrane composition, PE/PPE protein biogenesis, and the roles of mycobacterial surface components in immune evasion and nutrient acquisition. In Section 8, we cover bacterial capsules (Section 8), including their chemical diversity, biosynthetic pathways, and anti-phagocytic and complement-evasive functions, as well as their exploitation as vaccine targets. From surfaces, we then move on to a discussion of pili, fimbriae, and curli (Section 9) regarding their structural diversity, assembly mechanisms, and roles in adhesion, colonization, and immune activation. Finally, in Section 10, we describe secretion systems from Type I through Type XI, with emphasis on their architecture, mechanisms, and contributions to pathogenesis. Together, these sections provide an integrated view of the bacterial surface as a chemically diverse and complex environment.

**Figure 1:**
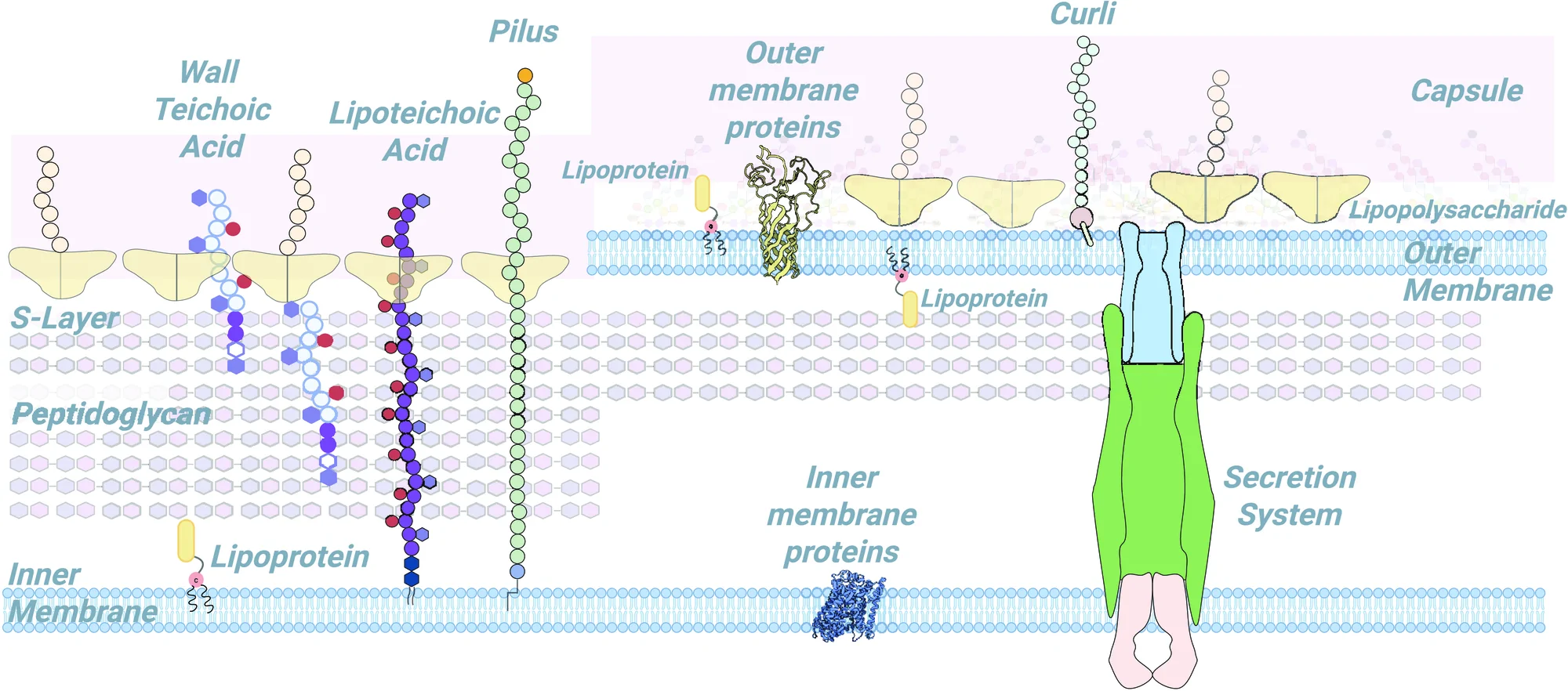
**Bacterial membranes, surfaces, and appendages.** The schematic illustrates the major bacterial surface structures and membrane components discussed in this review, organized from the innermost to the outermost layer. The IM houses integral IM proteins and lipoproteins anchored via lipid modifications. The PG (PG) layer, composed of alternating sugar residues crosslinked by short peptide stems, provides structural rigidity and is decorated with wall teichoic acid (WTA), which extends outward through the PG layer, and lipoteichoic acid (LTA), which is anchored in the IM. The S-layer, a crystalline array of surface layer proteins, is shown associated with the outer face of the PG layer. Where present, an OM contains LPS in its outer leaflet, integral OM proteins (OMPs), and lipoproteins anchored in its inner leaflet. Surface appendages including pili and curli fibers extend into the extracellular space and mediate adhesion and host interactions. A capsule, composed of polysaccharides, forms a diffuse outermost layer surrounding the cell. A multi-subunit secretion system is shown spanning from the cytoplasm through both membranes to the extracellular space. Together, these components represent the chemically diverse and architecturally complex bacterial surface whose biogenesis and roles in pathogen–host interactions are the subject of this review. Created in https://BioRender.com.

## 2 Overview of the Components and Organization of the IM

The IM is a phospholipid bilayer that forms the primary permeability barrier in all bacteria, separating the cytoplasm from the external environment. The IM is composed of chemically diverse glycerophospholipids whose specific composition varies across species and in response to environmental conditions. Embedded within or peripherally associated with this bilayer are three major classes of proteins: IMPs, which are predominantly α-helical and span the membrane via hydrophobic transmembrane segments; lipoproteins, which are anchored to the membrane surface through covalently attached lipid modifications; and peripheral MPs, which associate transiently with either face of the bilayer through electrostatic or hydrophobic interactions. Together, these components house the essential machinery for energy generation, nutrient transport, signal transduction, protein secretion, and cell envelope biosynthesis.

### 2.1 Overview and Structure of Lipids

Bacterial IM lipids are predominantly glycerophospholipids, which have a glycerol-3-phosphate backbone that is esterified at the *sn*-1 and *sn*-2 positions with fatty acyl chains, and they have a polar head group attached via a phosphodiester bond at the *sn*-3 position. Head group identity defines the lipid class: phosphatidylethanolamine (PE) has a zwitterionic headgroup, phosphatidylglycerol (PG) has a negatively charged headgroup, and cardiolipin (CL) has two phosphatidyl moieties linked through a central glycerol and, thus, has a dianion headgroup. Additional classes found across bacterial species include phosphatidylserine (PS), phosphatidylinositol (PI), and phosphatidylcholine (PC). Fatty acyl chain composition varies independently of headgroup. Bacterial membranes are composed of saturated, monounsaturated, and branched acyl chains through an FASII pathway. Glycolipids, in which one or more sugar residues replace the phosphate-containing headgroup, are also present in many bacterial IMs and serve as the lipid retention molecules in teichoic acid biosynthesis (Section 4).

#### 2.1.1 Overview and Structure of IMPs

IM proteins (IMPs) have transmembrane α-helices. The topology and folding of α-helical IMPs are governed by several physical and chemical principles. The most fundamental principle is the hydrophobic effect, which drives the partitioning of hydrophobic transmembrane segments into the lipid bilayer core.^2^ Transmembrane helices typically contain 18–25 hydrophobic residues, sufficient to span the ∼30 Å hydrophobic core of the membrane. The hydrophobicity threshold for membrane insertion can be quantified by biological hydrophobicity scales, with the Wimley-White scale being particularly predictive for bacterial IMPs.^3^

The “positive-inside rule” is a critical determinant of MP topology in bacteria. This principle states that positively charged residues (lysine and arginine) are preferentially located on the cytoplasmic side of the membrane, whereas the periplasmic loops tend to have fewer positive charges.^4,5^ This charge bias is likely due to two factors that may be related: the membrane potential and interactions with the Sec translocon. Experimentally inverting the charge distribution flips the topology of MPs, demonstrating the strength of this determinant.^6^

Helix–helix interactions within the membrane are mediated by specific packing motifs. The GxxxG motif allows close helix–helix contacts through glycine-mediated backbone interactions, while polar residues facing away from the helical contact site can form interhelical hydrogen bonds that stabilize oligomeric structures.^7^ Aromatic residues, particularly tryptophan and tyrosine, preferentially localize to the membrane-water interface regions, serving as ’anchors’ that position helices at the correct depth in the membrane.^8^

Lipid-protein interactions play crucial roles beyond simple hydrophobic matching. Anionic lipids such as PG and CL interact electrostatically with positively charged residues on protein surfaces, influencing both the localization and activity. The thickness and fluidity of the membrane also impact protein folding, hydrophobic mismatch between transmembrane helices, and bilayer thickness can drive conformational changes or protein aggregation.^9^ In bacteria that modulate membrane composition in response to temperature or pH stress by producing unique lipid species, lipid-protein interactions become particularly important for maintaining protein function.^10^

#### 2.1.2 Overview and Structure of Lipoproteins

Bacterial lipoproteins are a unique class of membrane-associated proteins that are anchored to either the periplasmic leaflet of IM or both the periplasmic and extracellular leaflets of the OM via lipid modifications (Figure 2). Lipoproteins perform diverse cellular functions critical to pathogenesis, including surface adhesion, signal transduction, and antibiotic resistance.^11,12^ Lipoproteins have a soluble domain that is anchored to the membrane at the N-terminus via a lipid moiety. The sequences and structures of the soluble domains of the mature lipoproteins are diverse. However, all preprolipoproteins start with an N-terminal signal sequence that is 10 to 25 residues long and ends with a consensus lipobox sequence, LAGC, with some variation in the first, second, and third positions. The signal sequence is cleaved (Figure 2) and the mature lipoprotein is formed.

**Figure 2:**
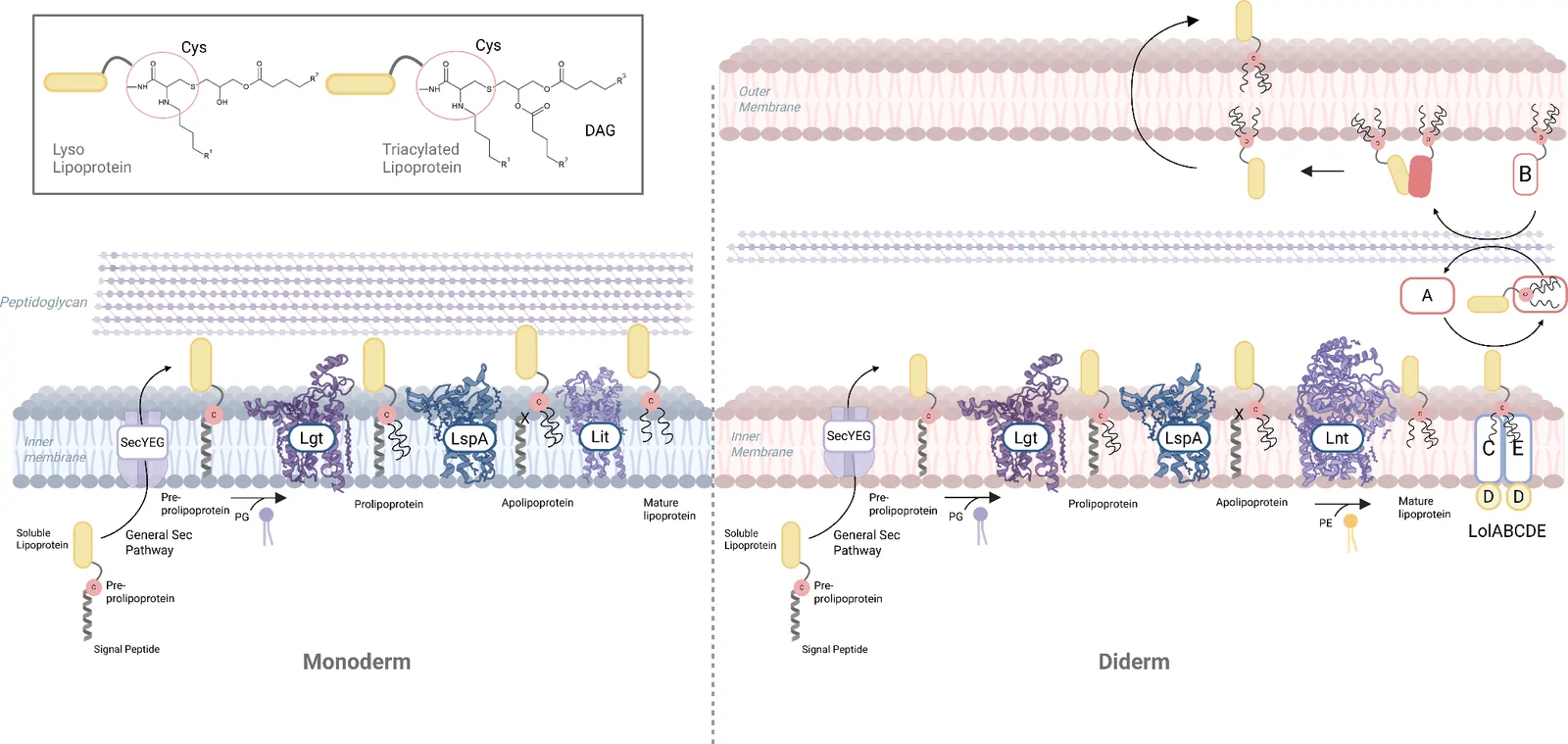
**Lipoprotein biogenesis and localization in monoderm and diderm bacteria.** Lipoproteins are synthesized as pre-prolipoproteins with N-terminal signal peptides that direct them to the Sec translocon (SecYEG) for membrane insertion. Following translocation, the conserved cysteine residue is lipidated by prolipoprotein diacylglyceryl transferase (Lgt), adding a diacylglycerol (DAG) moiety. The signal peptide is then cleaved by signal peptidase II (LspA/Lsp), generating an apolipoprotein. In diderm bacteria (right panel), an additional N-acylation by apolipoprotein N-acyltransferase (Lnt) produces triacylated lipoproteins (inset, chemical structure shown) that are sorted to the OM via the Lol trafficking system (LolABCDE). In monoderm bacteria (left panel), which lack an OM, diacylated lipoproteins remain anchored in the cell membrane. Organisms may contain different proteins that modify the apolipoprotein, such as lipoprotein intramolecular transacylase (Lit) that catalyzes intramolecular acyl transfer generating a lyso-lipoprotein. PE, phosphatidylethanolamine; PG, phosphatidylglycerol. Created with BioRender.com.

### 2.2 Biogenesis of the IM

The assembly of the IM requires coordinated biosynthesis and insertion of three distinct molecular classes: lipids, IMPs, and lipoproteins. Peripheral MPs are not covered in this Review due to the extensive scope. Lipids are synthesized in the cytoplasm and incorporated into the bilayer, establishing the physical foundations of the membrane. IMPs are inserted co- or post-translationally through dedicated translocon machinery, while lipoproteins undergo a conserved series of post-translational modifications before being anchored to the IM (or trafficked to the OM in diderm bacteria). Each of these processes is described in the following subsections.

#### 2.2.1 Lipid Biosynthesis

The bacterial type II fatty acid synthesis (FASII) pathway elongates alkyl chains through repeated cycles of condensation, reduction, and dehydration. This extensive and complex biosynthetic pathway has been discussed at length in various review articles; as such, this section will not cover it in detail.^13^ Beyond the canonical bacterial phospholipids such as phosphoethanolamine (PE), phosphatidylglycerol (PG), and cardiolipin (CL), bacteria have evolved a remarkable range of structural variations to adapt to their specific environments. Certain species produce unusual lipids, such as ornithine lipids (OLs), hopanoids (HOPs), glycolipids (GLs), and diacylglycerols (DAGs). The GLs are hypothesized to be glycerophospholipid substitutes under phosphorus-limiting conditions.^14^ OLs, while rare, contribute to greater bilayer order and contain a negatively charged carboxyl group rather than a phosphate group in glycerophospholipids.^15^ HOPs are structurally analogous to eukaryotic sterols and are thought to perform similar roles in modulating membrane fluidity and organization, including the possible formation of lipid nanodomains.^16^ Several lipid classes once considered exclusive to eukaryotes, such as ceramides, have been identified in bacteria through emerging biochemical and bioinformatics research. Thus, the lipid composition of bacteria has been an active area of research.

#### 2.2.2 IM Protein Biogenesis and Folding

IM proteins (IMPs) are integrated co-translationally via the Sec translocon or post-translationally via the YidC insertase.^17^ YidC is an insertase that can function independently or in cooperation with Sec translocon (also referred to as SecYEG). Some small IMPs with simple topologies insert via YidC alone, bypassing the Sec machinery entirely.^18^ YidC also assists with the folding of larger IMPs by stabilizing nascent chains and facilitating membrane integration. The mechanism of YidC-mediated insertion involves a hydrophilic groove that shields the hydrophobic transmembrane segments during membrane crossing.^19^ However, the Sec pathway is the primary route for most of the IMPs. The Sec system mediates the translocation and insertion of polypeptides in and across the IM and is the most conserved and universally distributed protein export pathway in eukaryotes, prokaryotes, and archaea.^20^ SecYEG is a heterotrimeric membrane-embedded channel that forms a protein-conducting pore with a central constriction and a lateral gate opening into the lipid bilayer, allowing integration of hydrophobic segments into the membrane.^17,21,22^ In addition to IMPs, Sec translocon substrates include periplasmic enzymes, prematurely unfolded OMPs, secreted enzymes, cell envelope assembly factors such as BamA and LptD, and numerous other proteins, which all have an N-terminal signal peptide that is typically cleaved after translocation.^20,23^ The Sec pathway was identified through genetic and biochemical studies in the 1970s and 1980s, and its structural basis was elucidated at high resolution by X-ray crystallography and cryo-electron microscopy. These structures revealed multiple conformational states corresponding to closed, primed, and actively translocating conformations.^21,22,24,25^

Translocation through the Sec channel is driven by the coordinated action of ATP hydrolysis and the proton motive force across the IM.^26,27^ A cytoplasmic peripheral ATPase (SecA) associates with the Sec complex and drives translocation of the unfolded protein through the Sec channel.^28,29^ Unusually long stretches of hydrophobic signal sequences or transmembrane domains are recognized by the signal recognition particle (SRP) as they emerge from the ribosome, targeting the ribosome-nascent chain complex directly to the SecYEG translocon at the membrane.^30^ Cycles of ATP binding and hydrolysis induce conformational changes in the cytosolic ATPase that promote directional insertion of successive polypeptide segments into the channel.^28,31,32^ At the same time, the electrochemical gradient across the membrane contributes to directional transport, likely by biasing diffusion of the polypeptide toward the periplasm and suppressing backward movement.^31,33^ The channel itself exhibits pronounced conformational flexibility: a plug domain occludes the pore in the resting state, the central constriction formed by hydrophobic residues expands during translocation, and the lateral gate opens to permit partitioning of transmembrane helices into the bilayer.^21,22,24,25^ Translocation can also occur post-translationally through the chaperone SecB, which binds newly synthesized preproteins and stabilizes them in an unfolded state, preventing aggregation.^34^ SecB then shuttles and transfers the bound, unfolded protein across the cytoplasm to the membrane-bound SecA channel for further translocation.^34^ Despite extensive structural and biochemical characterization of the Sec translocon, several fundamental questions about its mechanism remain unresolved. These include how the ATPase conformational cycles are coupled to substrate movement, what are the relative contributions of power-stroke versus Brownian ratchet models of translocation, and how is substrate flux regulated under conditions of high demand.^31,35–44^

The substrates of the Sec pathway are initially unfolded at the start of translocation; however, some proteins need to be properly folded in the cytoplasm and translocated across the IM. For these proteins, an alternative translocation system was used. The Tat (twin-arginine translocation) system is an additional protein insertion and export pathway that mediates the insertion or translocation of fully folded proteins across the IM.^45–47^ In contrast to the Sec system, which transports unfolded substrates, the Tat pathway accommodates proteins that have already acquired tertiary structure, often including tightly bound cofactors, such as metal centers, complex prosthetic groups, or assembled multimeric domains.^48^ The core Tat architecture comprises a membrane-embedded receptor complex that recognizes N-terminal signal peptides bearing a conserved twin-arginine motif, together with a dynamic oligomeric pore-forming assembly recruited upon substrate engagement.^49–51^ These components are distributed within the IM and undergo substrate-induced reorganization to form a transient translocation conduit.^50,52,53^ In diderm bacteria, translocated Tat substrates are delivered to the periplasm, where they perform functions such as redox catalysis, cofactor-dependent enzymatic reactions, cell envelope biogenesis, and virulence-associated processes.^45–47^ The Tat pathway was identified in the 1990s through genetic studies of protein export, independent of the Sec system. Subsequent structural work using cryoelectron microscopy, electron tomography, and biochemical reconstitution has revealed that the system assembles into variable-sized oligomeric complexes rather than a pre-formed, static channel.^49–53^

The Tat system is driven exclusively by the proton motive force across the IM without direct coupling to ATP hydrolysis.^47,54,55^ Substrate proteins bearing the twin-arginine signal peptide are first recognized by the membrane receptor complex, which triggers the recruitment and assembly of additional membrane components into a transient, size-adaptive pore that is capable of accommodating folded substrates of varying dimensions.^50,53,56^ Current mechanistic models propose that the proton gradient induces conformational changes and/or local membrane perturbations that facilitate protein translocation, although the precise coupling mechanism remains unresolved.^57,58^ A distinctive feature of the Tat pathway is its apparent ability to preserve membrane integrity while translocating large, folded proteins, suggesting that the translocation may be highly dynamic and involve localized thinning or transient lipid-bilayer disruption.^58–60^ Despite significant progress in understanding the Tat translocation mechanism, several key questions remain. These include how the system discriminates between properly folded and misfolded substrates, how pore size is regulated in response to substrate geometry, and how proton motive force is mechanistically coupled to large-scale membrane remodeling.^58,61–63^

Quality control of the IM is vital in both monoderm and diderm bacteria. The AAA+ protease FtsH recognizes and degrades misfolded IMPs, preventing the accumulation of non-functional proteins. The chaperone DnaK/DnaJ system also plays a role in maintaining IMP integrity, particularly for proteins with large periplasmic or cytoplasmic domains.^64^ In addition to chaperones, phospholipids influence the IMP folding and stability. Anionic lipids such as CL and PG cluster around certain IMPs, stabilizing functional oligomers.^65,66^ The asymmetric distribution of lipids between the inner and outer leaflets appears to differ between monoderm and diderm bacteria, suggesting distinct lipid microdomains that may influence the protein topology and function.^67^

#### 2.2.3 IM Lipoprotein Biogenesis

In both monoderm and diderm bacteria, lipoproteins are matured through a conserved post-translational processing pathway (Figure 2) that consists of a series of enzymes localized to the IM.^11^ The biogenesis of all lipoproteins (IM or OM) starts with the recognition of the N-terminal signal sequence by either the Sec or the Tat translocation system, which directs the nascent lipoprotein to the IM. Once at the membrane, diacylglyceryl transferase (Lgt) cleaves a DAG moiety from an endogenous PG lipid and covalently attaches it to a conserved cysteine residue within the lipoprotein motif.^11^ Following this diacylation, lipoprotein signal peptidase II (LspA) cleaves the N-terminal signal peptide.^11^ Finally, a variable third acyl modification occurs, which is dependent on the bacterial species or a specific lipoprotein. This modification may involve N-acylation by lipoprotein N-acyltransferase (Lnt) or transacylation by intramolecular lipoprotein transferase (Lit).^11^ Upon completion of this pathway, the mature lipoproteins are competent to perform their cellular functions. Lgt and LspA are essential in diderm bacteria,^68,69^ and Lnt and Lit are not essential in certain bacteria. However, for some bacteria, such as *Helicobacter pylori* ^70^ and *Streptococcus pneumoniae*,^71^ Lnt is not essential for survival but is essential for colonization.

In monoderm bacteria, mature lipoproteins either remain associated with the IM to perform their functions or are secreted into the environment. In diderm bacteria, many IM lipoproteins play roles in cell viability and overall maintenance, including membrane homeostasis, envelope integrity, and stress-signaling responses.^72^ Although these functions are vital for cell survival, they are not directly involved in infection pathways or pathogenesis. Lipoproteins in diderm bacteria can be transported to the OM and is described in Section 6.2.4.

### 2.3 Role of IM in Pathogenesis

The IM interacts directly and indirectly with the host pathogens. In monoderm bacteria, the IM components are more directly exposed to host immune surveillance and can engage host receptors or modulate immune signaling. In diderm bacteria, the IM is further shielded by the OM and its pathogenic contributions are largely indirect. The IM provides the structural foundation for translocation complexes, secretion systems, lipid trafficking, and surface-display pathways that arm the bacterial exterior with virulence factors. Across both architectures, the lipids, integral proteins, and lipoproteins of the IM collectively shape the outcome of infection through mechanisms ranging from direct immune activation to support surface remodeling and toxin delivery.

#### 2.3.1 IM Lipids in Pathogenesis

Beyond proteins and lipoproteins, the lipids comprising the IM bilayer also contribute significantly to pathogenesis. Bacteria use many lipid moieties as carriers during the synthesis of surface-layer components (e.g., PGs, teichoic acids, and polysaccharides). These surface layers extend into the extracellular space and serve both as structural and pathogenic functions. Pathogenic bacteria have evolved additional mechanisms to adapt their membranes to host environments. For instance, during infection, *S. aureus* acquires exogenous unsaturated fatty acids from host cells and incorporates them into its membrane lipids. These acquired fatty acids increase the production of branched-chain fatty acids, which reduces lipoprotein-mediated toll-like receptors (TLR)­2 activation.^73^ An example of membrane reorganization in bacteria in response to antibiotic delivery after infection is found in *Enterococcus faecalis*, which utilizes exogenous oleic acid to increase resistance to the membrane-targeting antibiotic daptomycin.^74^ Daptomycin, and other membrane-disrupting antimicrobials such as amphotericin B or polymyxin B, intercalate into microbial membranes, where the partitioning of these molecules/peptides disrupts the stability of the cell membrane, leading to cell death.^74,75^ Daptomycin is positively charged and partitions into the membrane through interactions with negatively charged phospholipid head groups, such as PG. Studies have shown that *E. faecalis* cells that are not readily resistant to daptomycin scavenge oleic acid and incorporate it into their membranes, leading to a downstream decrease in PG synthesis and conferring resistance to the molecule.^74^ The obligate bacterium *Chlamydia trachomatis* relies on scavenging oleic acids from host cells to form unsaturated fatty acids.^76^ Acyltransferase LpaT and acyl-ACP synthetase AasC enable the bacterium to incorporate and synthesize phospholipids not endogenously produced by the cell, thereby saving energy during infection for other biosynthetic pathways.^77^

#### 2.3.2 IM Proteins in Pathogenesis

The IM machinery is responsible for supporting PG synthesis, sorting, and modifying lipoproteins and OMPs, supporting secretion systems, assembling pili, and much more. Because of this central role, several components are involved in the pathogenesis. Secretion systems (Section 10), such as the Type III secretion system (T3SS), span the entire cell envelope. A portion of the complex is embedded in the IM and extends through the periplasm and the OM to reach the extracellular space, where it secretes proteins from the cell. Thus, the IM provides a supporting role in infection by containing anchoring components of the secretion systems.^78^ Similarly, the IM supports host-cell interaction by trafficking LPS to the OM, specifically via the LptFGC protein complex, which accounts for half of the LPS trafficking complex.^79^ Although lipids and IMPs provide a more indirect link to pathogenesis, some lipoproteins, particularly in monoderm bacteria, play pathogenic roles.

#### 2.3.3 IM Lipoproteins in Pathogenesis

Monoderm IM lipoproteins fulfill diverse roles in pathogenesis, including the acquisition and transport of extracellular iron.^80^ Iron is essential for bacterial growth, yet its limited bioavailability in the host necessitates specialized uptake systems for a successful infection. In *S. aureus*, the number of lipoprotein-mediated iron uptake systems correlates positively with pathogenicity.^81^ *S. aureus* encodes eight lipoproteins involved in iron acquisition and transport, including the well-characterized proteins FhuD1 and FhuD2. These lipoproteins bind iron­(III)-hydroxamate siderophores and transfer them to the adenosine triphosphate binding cassette (ABC) transporter FhuCBG.^82^ Bacterial lipoproteins also bind to amino acids, peptides, and lipids to assist in transport. Other bacterial lipoproteins are critical to fitness and pathogenicity. For instance, PrsA is a highly conserved surface lipoprotein found across many monoderm bacterial species, including *S. pneumoniae*, *B. subtilis*, *S. aureus*, and others.^83^ PrsA, a parvulin-type peptidyl-prolyl *cis/trans*-isomerases (PPIase), catalyzes the structural rotation of proline residues between *cis* and *trans* conformations and is crucial for post-translational protein folding at the membrane.^84^ Although PrsA is highly conserved across monoderm bacteria, the substrates differ across species. In group A *Streptococcus*, PrsA inactivation resulted in a significant decrease in active exotoxin B (SpeB), as well decreased adherence to host cells, biofilm formation, and virulence.^85,86^ Like PrsA, several other lipoproteins interact directly with host proteins and cells.

Some bacterial lipoproteins interact with TLRs on the host cells and engage in cell invasion. Secreted lipoproteins bind TLR2 through their lipid modification at the N-terminus and trigger a cascading immune response from the host.^87^ A genomic cluster of lipoproteins in *S. aureus* contributes to internalization of the bacterium into nonprofessional antigen-presenting cells and is, thus, directly involved in bacterial virulence.^88^ Although there are numerous examples of lipoprotein directly facilitating host interactions, there are still many lipoproteins of unknown function that need to be investigated.

#### 2.3.4 Outer-IM Vesicles in Pathogenesis

Mercade *et al.* report in multiple publications that outer-IM vesicles (O-IMVs) are secreted by various pathogenic diderm bacteria such as *Neisseria gonorrhoeae, Pseudomonas aeruginosa*, and *Acinetobacter baumanii*.^89^ These O-IMVs contain periplasmic proteins, LPS, and other OM lipids, cytoplasmic proteins, and other components such as DNA, ATP, and IM proteins.^89^ Proteomic analysis of O-IMVs from *N. gonorrhoeae* found that 13.7% of the proteins were IM and 18% were cytoplasmic proteins.^89^ The thiol:disulfide interchange protein DsbD and pyruvate dehydrogenase were found in the O-IMVs.^89^ *Shewanella vesiculosa* M7^T^ increases in O-IMV secretion during the late exponential to early stationary phase.^90,91^ In response to prophage-mediated explosive cell lysis, single- and double-layer O-IMVs were observed.^90,91^ O-IMVs may facilitate the delivery and secretion of bacterial cytoplasmic and IM components providing an additional mechanism of host interactions.

### 2.4 IM Therapeutic Targets

#### 2.4.1 IM Antibiotic Targets

The bacterial IM houses several essential biosynthetic and assembly machineries that are either absent from or sufficiently divergent in eukaryotic cells to permit selective therapeutic intervention. Many IM components and processes are thus promising antibiotic targets, with inhibition often resulting in bactericidal activity due to downstream effects on cell envelope integrity. The bacterial type II fatty acid synthesis system, a set of discrete monofunctional enzymes distinct from the mammalian fatty acid synthesis pathway, is an antibiotic target. FabI, the enoyl-acyl carrier protein reductase, catalyzes the final reductive step of the fatty acid elongation cycle and is the most advanced target in this pathway. Triclosan and isoniazid established proof of concept for FabI inhibition. Afabicin (Debio 1450), a prodrug targeting staphylococcal FabI, completed a Phase 2 clinical trial for acute bacterial skin and skin structure infections caused by *staphylococci*.^92^ CG400549, another FabI inhibitor, is also in clinical development for staphylococcal infections.^93^ The mycobacterial FabI homolog, InhA, is inhibited by isoniazid. However, isoniazid is a prodrug that requires activation by a catalase-peroxidase. InhA inhibitors that bypass this activation step are under development to address resistance arising from catalase-peroxidase mutations.^94^ FabH, the β-ketoacyl-ACP synthase III that initiates each elongation cycle, represents an underexploited but promising target with diverse inhibitor scaffolds reported in preclinical studies.^95^

The lipoprotein processing enzyme LspA (Figure 2), is essential for survival in diderm bacteria and is inhibited by the natural cyclic depsipeptides globomycin and myxovirescin. Crystal structures of methicillin-resistant *S. aureus* (MRSA) LspA bound to both compounds revealed provided a structural framework for inhibitor design.^96^ Computationally designed cyclic peptide analogues of globomycin with improved stability exhibit activity against a group of highly virulent, antibiotic-resistant bacteria responsible for most global hospital-acquired infections.^97^

In mycobacteria, the IM MmpL3 exports trehalose monomycolate across the IM as an obligate step in mycolic acid cell wall biosynthesis (see Section 7) and is the target of multiple structurally diverse compound classes. SQ109, an ethambutol analog and MmpL3 inhibitor, has completed Phase 2*b*/3 clinical trials for MDR pulmonary tuberculosis and shows synergistic activity with front-line agents including isoniazid, rifampicin, and bedaquiline.^98^

#### 2.4.2 IM Vaccine Targets

The IM is the most occluded bacterial surface from the extracellular space and does not present antigens readily recognized by the host immune system, making it a poor source of vaccine candidates compared with outer-surface components. Despite this limitation, IM lipoproteins can achieve a surface exposure. The iron-acquisition lipoprotein FhuD2 of *S. aureus* similarly achieves surface exposure despite IM anchoring and has been developed as a vaccine antigen. FhuD2 is included in the multicomponent 4C-Staph formulation, alongside the conserved lipoprotein Csa1A and secreted virulence factors. This formulation showed protection across multiple murine infection models.^99^ Beyond their roles as direct antigens, the lipid modifications added to lipoproteins at the IM by Lgt confer adjuvant activity that stimulates TLR2. This stimulatory property is exploited to enhance the immunogenicity of recombinant vaccine antigens by engineering the lipoprotein signal peptide and lipobox into heterologous proteins.^100^

## 3 Overview of the Components and Organization of the PG Layer

PG is a complex, unique essential macromolecule of bacterial cells comprised of alternating *N*-acetylglucosamine (NAG) and *N*-acetylmuramic acid (NAM), with short oligopeptides attached to the NAM sugar (Figure 3).^101,102^ The peptide chain can then be crosslinked to other peptide chains, forming the full structure of the PG layer. The PG layer is synthesized, cross-linked, and remodeled by glycosyltransferases, transpeptidases, and hydrolases (Figure 3).^103^

**Figure 3:**
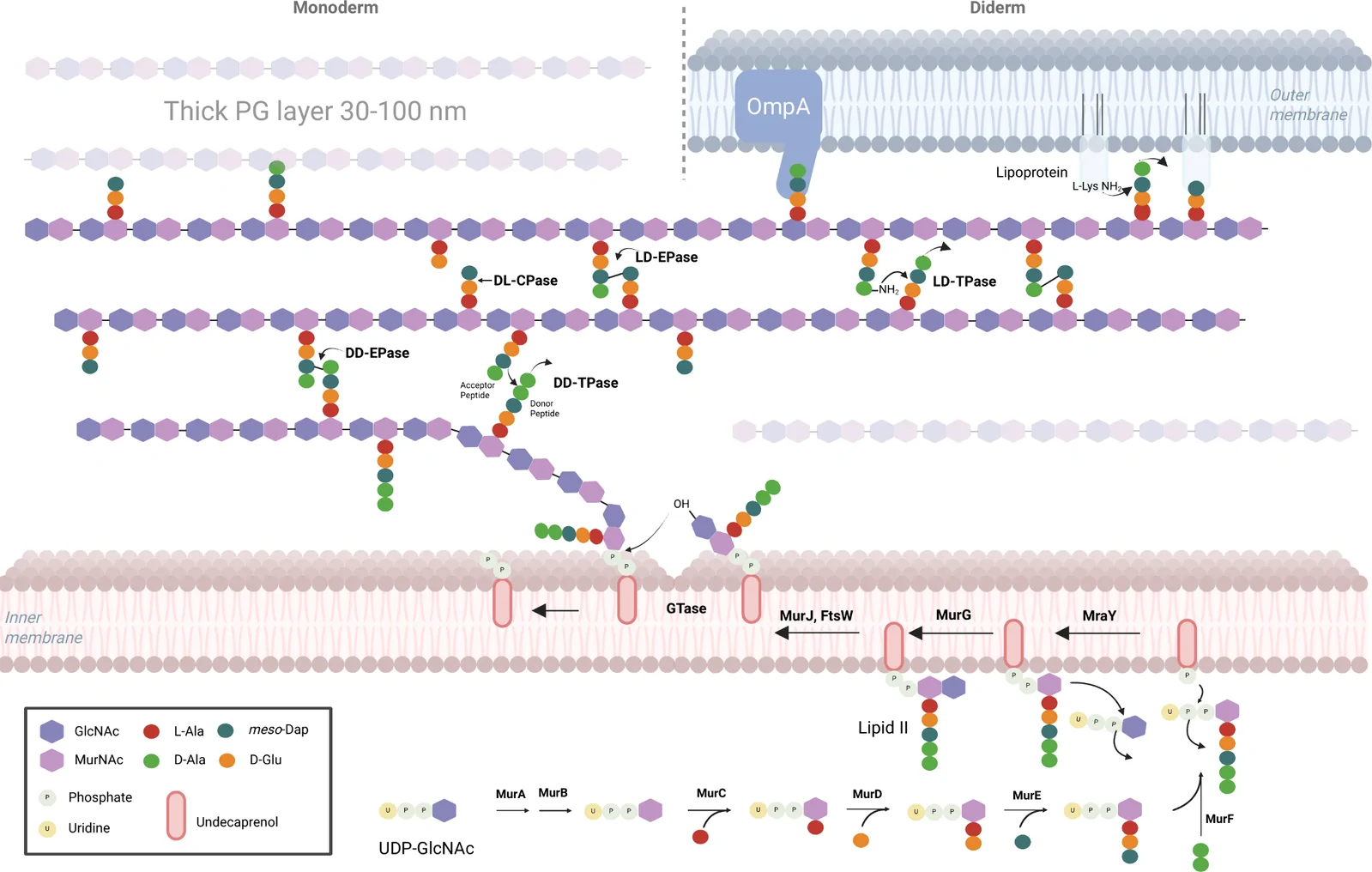
**PG biosynthesis and remodeling in monoderm and diderm bacteria.** Cytoplasmic synthesis begins with UDP-GlcNAc, which is converted to UDP-MurNAc by MurA and MurB. The Mur ligases MurC, MurD, MurE, and MurF sequentially add l-alanine (red), d-glutamate (orange), *meso*-diaminopimelic acid (*meso*-Dap, teal), and a d-alanine-d-alanine dipeptide (green) to form the pentapeptide stem. MraY transfers the sugar-peptide unit to undecaprenyl phosphate to form Lipid I; MurG then adds GlcNAc to produce Lipid II. The flippases MurJ and FtsW translocate Lipid II to the periplasmic face, where glycosyltransferases (GTase) polymerize glycan strands. DD-transpeptidases (DD-TPase) crosslink adjacent peptide stems via acceptor and donor peptides, while DD-endopeptidases (DD-EPase) cleave crosslinks and DL-carboxypeptidases (DL-CPase) trim peptide stems. LD-transpeptidases (LD-TPase) form an alternative crosslink type and, in diderms, covalently attach Lpp to the PG via l-Lys NH_2_. In diderms (right), OmpA associates non-covalently with the PG layer, and lipoproteins are anchored covalently. The monoderm PG layer (left) is substantially thicker (30–100 nm) than that of diderms. Created in https://BioRender.com and adapted from Figure 1 in.^111^ Available under a CC-BY-SA 4.0 license. Copyright 2020 Egan et al.

PG can be covalently modified by proteins carrying C-terminal sorting sequences, such as LPXTG and NPQTN (Figure 4). There are six families of sortase enzymes (SrtA–F), each classified based on primary sequence. Sortase A, the prototype of class A, catalyzes the covalent attachment of LPXTG-bearing surface proteins to the PG (Figure 4). Substrate proteins are first exported across the cytoplasmic membrane by the Sec machinery, then folded by an as-yet uncharacterized post-translocational mechanism and retained at the membrane via their C-terminal cell wall sorting signal (CWSS). Sortase A cleaves the LPXTG motif between threonine and glycine residues,^104^ forming a thioester-linked acyl-enzyme intermediate. The substrate protein is then transferred to lipid II through a nucleophilic attack from the amino group of the pentaglycine crossbridge on Lipid II.^105^ Transpeptidases and glycosyltransferases subsequently incorporate the Lipid II-linked protein into the mature PG network. The role of sortase enzymes in pilus polymerization and cell wall attachment during pili biogenesis is discussed in Section 9.2.3.

**Figure 4:**
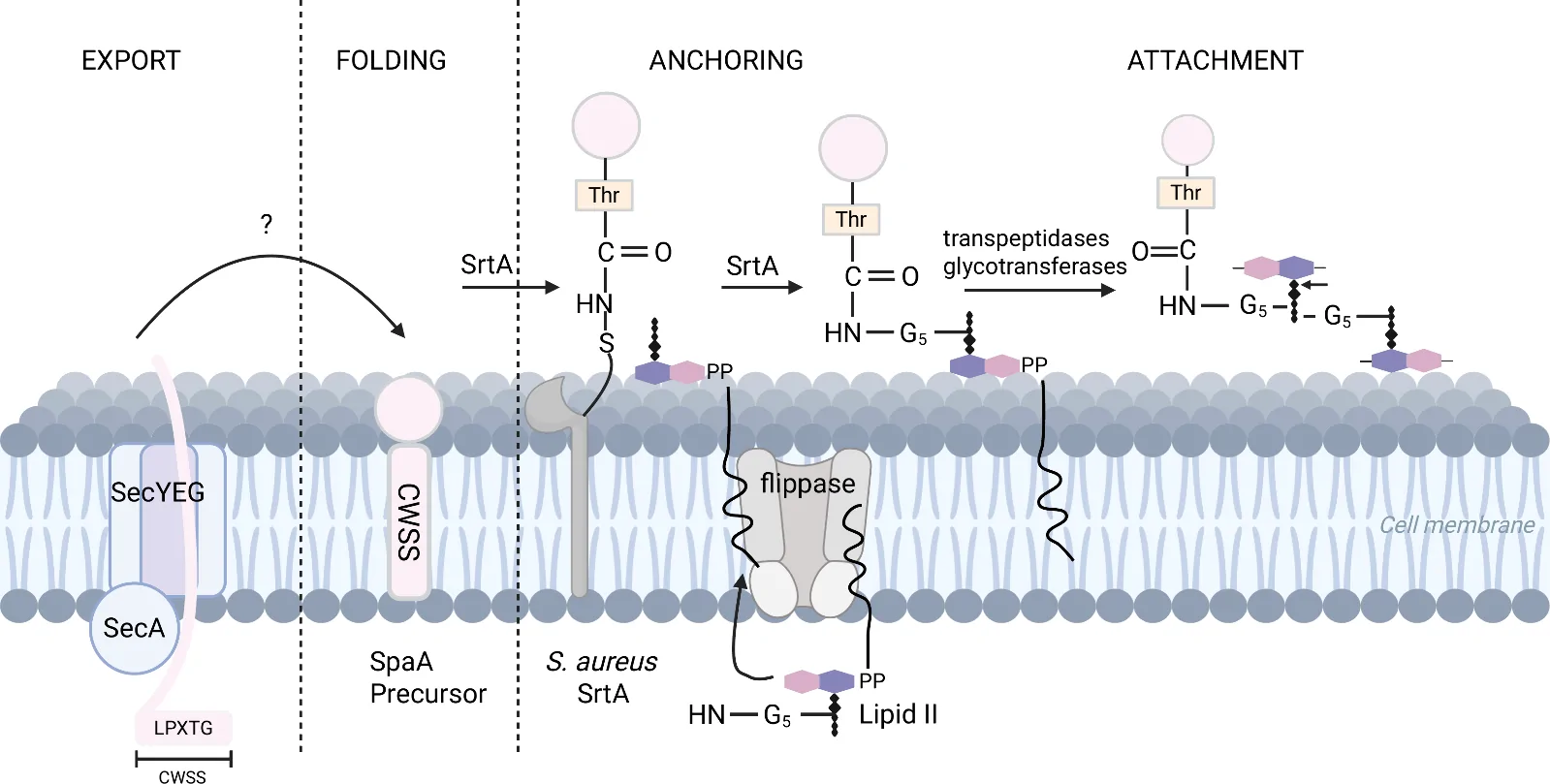
**Sortase A-mediated surface protein anchoring in monoderm bacteria.** The four sequential stages of sortase A (SrtA)-mediated covalent attachment of LPXTG-bearing surface proteins to the PG (PG) cell wall are shown, modeled after *S. aureus*. In the Export stage, the substrate protein (SpaA precursor) carrying an N-terminal signal peptide and C-terminal cell wall sorting signal (CWSS), composed of an LPXTG motif followed by a hydrophobic domain and positively charged tail, is translocated across the cytoplasmic membrane by the Sec translocon (SecA). In the Folding stage, the translocated precursor undergoes posttranslocational folding by an unknown mechanism (indicated by “?”). In the Anchoring stage, the folded precursor is retained at the membrane via its CWSS, where SrtA cleaves the LPXTG motif between threonine and glycine, forming a thioester-linked acyl-enzyme intermediate (C=O–S). The amino group (NH) of the pentaglycine (G_5_) crossbridge within the Lipid II PG precursor attacks the thioester, transferring the substrate to Lipid II. In the Attachment stage, transpeptidases and glycosyltransferases incorporate the Lipid II-linked protein into the mature PG network, covalently anchoring it to the cell wall for stable surface display. This mechanism underlies both individual surface protein anchoring and, with class C and E sortases, the polymerization and anchoring of sortase-assembled pili (Section 9.1.3). Created in https://BioRender.com and adapted from Figure 1 in^545^ and Figure 1 in.^920^ Figure 1 in ref 545 is available under a CC-BY 4.0 license (Copyright 2024 Chang et al.); Figure 1 in ref 920 is reproduced with permission (Copyright 2011 John Wiley and Sons).

### 3.1 PG Biogenesis

PG biosynthesis (Figure 3) begins in the cytoplasm, where the precursor UDP-N-acetylmuramic acid (UDP-MurNAc) is assembled from UDP-N-acetylglucosamine (UDP-GlcNAc) through the sequential action of MurA and MurB, which is then built into a pentapeptide stem by MurC, MurD, MurE, and MurF.^106,107^ MraY and MurG convert this product into lipid II, where flippases such as MurJ flip the lipid II across the IM to the periplasmic face.^108–110^ Glycosyltransferases polymerize the glycan strands and penicillin binding proteins (PBPs) crosslink the peptide stems to a form a mesh-like network which is continuously remodeled throughout the bacterial cell cycle to accommodate cell elongation, division, and shape maintenance.^111^ In diderms, LD-transpeptidases (LD-TPases) form additional crosslinks and covalently attach the lipoprotein Lpp to the OM in *E. coli*. Additionally, the PG can be non-covalently modified with OMPs, such as OmpA, which further stabilizes the cell wall.^112^

While the core biosynthetic machinery is broadly conserved across bacteria, the architecture and composition of the final PG layer differ considerably between monoderm and diderm bacteria. Monoderm bacteria, such as *B. subtilis* and *S. aureus*, possess a thick, multilayered PG wall that ranges from 30 to 100 nm in thickness and is often heavily crosslinked.^113,114^ This robust barrier is further decorated with accessory polymers such as wall teichoic acids, which are covalently attached to PG and contribute to cell wall rigidity, surface charge, and ion homeostasis.^115,116^ Teichoic acids are discussed in Section 4. In diderm bacteria, the PG layer is significantly thinner than in monoderm bacteria, measuring roughly 2 to 7 nm and located within the periplasmic space.^102,113^ The periplasmic space spans approximately 13–25 nm in width in *E. coli* and is not a fixed compartment; cryo-electron microscopy studies show it behaves as a compressible gel whose width varies with osmotic conditions.^117^ In diderm bacteria, the PG layer must be physically anchored to the OM to maintain envelope integrity, a function carried out by a subset of OM lipoproteins and OMPs.^118,119^ The degree of peptide crosslinking is less in diderm bacteria compared to their monoderm counterparts. The composition of PG differs as well, with *meso*-Dap acid found in most diderm species and l-lysine more common in monoderm bacteria.^113^ These chemical and structural differences have important implications for antibiotic targeting, immune recognition, and the overall mechanical properties of the bacterial cell envelope.

### 3.2 PG in Pathogenesis

The PG layer is a biological stimulant in host–pathogen interactions, leading to cytotoxicity and activation of macrophages. The innate immune system recognizes muropeptides as PG fragments. Muropeptides are recognized by host cell receptors nucleotide-binding oligomerization domains (NOD)­1 and NOD2.^120,121^ NOD1 and NOD2 act as an immune alarm system by triggering rapid inflammatory and antimicrobial responses. NOD1 specifically recognizes a *meso*-Dap-containing muropeptide found exclusively in diderm bacterial PG, while NOD2 recognizes muramyl dipeptide (MDP), a fragment present in both monoderm and diderm bacterial PGs.^122^ *Legionella pneumophila* has adapted to this immune response by modifying its PG with PgdA, a deacetylase that converts N-acetylglucosamine (NAG) in PG to glucosamine.^123^ This PG modification reduces cleavage of PG by lysozymes, decreases murapeptide release, and thus evades NOD receptor recognition.^123^

Internalin, InlA, in *L. monocytogenes*, is a PG-bound surface protein that interacts with and binds host E-cadherin molecules, which contributes to adhesion and internalization of the bacteria.^124^ Another family of cell wall PG-anchored virulence factors is microbial surface components recognizing adhesive matrix Molecules (MSCRAMM),^125^ SdrF, a *S. epidermis* MSCRAMM, participates in the initial adhesion to host cells by binding type 1 collagen and keratins.^126^ Other MSCRAMMs bind to fibronectin in host cells, promoting cell adhesion and facilitating infection.^127^

### 3.3 PG Therapeutic Targets

#### 3.3.1 PG Antibiotic Targets

PG is the most structurally and therapeutically exploited component of the bacterial cell envelope. PG has the most abundant antibiotic targets identified to date because of its role in maintaining cellular integrity across virtually all bacteria, its absence from eukaryotic cells, and the accessibility of its biosynthetic pathway at multiple chemically distinct steps.^128^ Therapeutics targeting each biosynthetic step have yielded clinically important antibiotics, and several are under active development.^128,129^

The extracellular cross-linking step catalyzed by PBPs is one of the oldest known antibiotic targets. β-Lactam antibiotics (e.g., penicillins, cephalosporins, carbapenems, and monobactams) inactivate PBPs by covalently mimicking the d-Ala-d-Ala terminus of the peptide stem, blocking transpeptidation.^128^ Resistance driven by the acquisition of PBP2a in MRSA is the paradigmatic clinical problem: PBP2a has low β-lactam affinity and requires allosteric activation by cell wall fragments at a site distal to the active site. Current strategies focus on small-molecule adjuvants that bind this allosteric site to restore β-lactam activity.^130^ Glycopeptide antibiotics target the lipid II pentapeptide and thereby block its incorporation into PG. The lipoglycopeptides dalbavancin, oritavancin, and telavancin extend this mechanism with additional membrane-anchoring moieties that improve activity against vancomycin-resistant strains.^131^ Lipid II itself has emerged as a particularly attractive target because it is the single essential convergence point of the PG biosynthetic pathway. Clovibactin, isolated from an uncultured soil bacterium, kills drug-resistant Gram-positive pathogens without detectable resistance by binding the pyrophosphate moiety common to multiple lipid-anchored PG precursors (lipid II, lipid III, and C55-PP).^132^ At the membrane-associated biosynthetic steps, the phospho-MurNAc-pentapeptide translocase (MraY) that generates lipid I has no commercially available inhibitor despite being essential in all bacteria. Natural product nucleoside antibiotics including caprazamycins, muraymycins, and riburamycins inhibit MraY with broad-spectrum activity and low toxicity in preclinical studies; however, incomplete mechanistic understanding of inhibitor binding continues to hinder clinical translation.^133^ PG hydrolases, which remodel PG during growth and division, represent a recently recognized target class. Small-molecule inhibitors of these enzymes are in early development and show synergistic activity with existing antibiotics.^134^ The broader preclinical pipeline includes inhibitors of the cytoplasmic ligases as well as the glucosamine-1-phosphate acetyltransferase/uridyltransferase that synthesize the UDP-GlcNAc substrate required in PG and LPS biosynthesis.^129^

#### 3.3.2 PG Vaccine Targets

PG is surface-accessible and therefore immunologically visible, yet it has not yielded direct vaccine antigens in a conventional sense. Unfortunately, the NOD and murapeptide interactions are too non-specific to develop an adaptive vaccine response, but MDP, the minimal NOD2-active PG fragment, has a long history as a vaccine adjuvant. MDP stimulates cytokine production and enhances adaptive immune responses to co-administered antigens.^135^ Clinical application of native MDP has been limited by pyrogenicity, rapid elimination, and poor oral bioavailability; extensive structure-activity relationship studies have produced synthetic analogues with reduced pyrogenicity and improved pharmacokinetic profiles, and several MDP-based adjuvants are under investigation for use with bacterial and viral vaccine antigens.^135,136^ PG itself is not a direct vaccine antigen because the PG backbone is highly conserved across both pathogenic and commensal bacteria and because the adaptive immune response to PG is predominantly innate and T-cell-independent.^137^

## 4 Overview of the Structure and Organization of Teichoic Acids

Monoderm PG layers can be modified with glycolipid polymers known as teichoic acids (TAs; Figure 5) that extend outward from the cell. TAs can be anchored to the cell membrane as lipoteichoic acids (LTA) or covalently linked to the PG as wall teichoic acids (WTA) (Figure 5). Both LTAs and WTAs are unique to monoderm bacteria and have roles in biofilm formation and bacterial adhesion as well as controlling cell shape and cell division.^115^ LTA has polymerized glycerol phosphate repeat units and is covalently attached to PG. WTAs have a disaccharide base, covalently attached to PG, and a long polymer of sugar phosphate units (e.g., glycerol phosphate or ribitol phosphate) linked through a phosphodiester bond. WTAs are anchored to the outer leaflet of the IM through a covalent linkage to a glycolipid. TA chemical modifications identified include acylation (e.g., d-alanine), esterification (of the d-alanine modifications), choline, and various sugar moieties, such as GlcNAc.^138,139^ TA protein modification occurs when choline-binding proteins (CBPs) bind to TAs, decorating the bacterium’s surface. CBPs are a diverse protein family that non-covalently bind to phosphorylcholine moieties via a choline binding domain found on both lipoteichoic and wall teichoic acids.^140^

**Figure 5:**
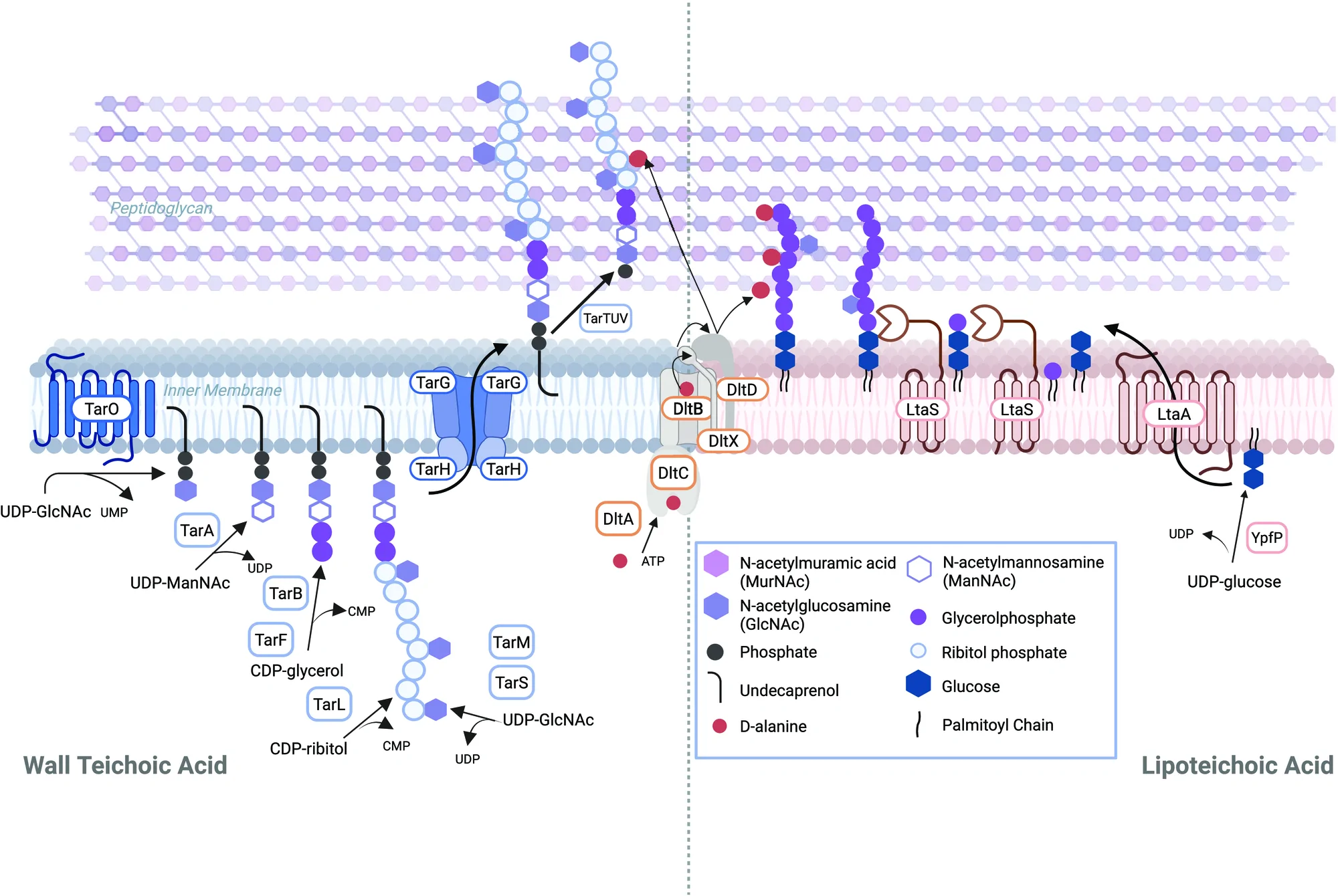
**Biosynthesis pathways of wall teichoic acid (WTA) and lipoteichoic acid (LTA) in** *S. aureus* **.** WTA biosynthesis (left) is initiated in the cytoplasm by TarO, which transfers N-acetylglucosamine-1-phosphate from UDP-GlcNAc onto the lipid carrier undecaprenyl phosphate, releasing UMP. TarA adds N-acetylmannosamine (ManNAc) from UDP-ManNAc, and TarB transfers the first CDP-glycerol unit. TarF extends the linkage unit, and TarL polymerizes the ribitol-phosphate backbone from CDP-ribitol. The nascent WTA polymer is modified by TarM (α-GlcNAc glycosylation) and TarS (β-GlcNAc glycosylation) from UDP-GlcNAc in the cytoplasm, before the ABC transporter complex TarGH flips the completed polymer across the IM. TarTUV translocate the mature WTA to the extracellular face of the PG layer for covalent attachment to MurNAc residues. LTA biosynthesis (right) occurs at the outer leaflet of the IM. YpfP synthesizes the diglucosyl-diacylglycerol (Glc_2_-DAG) glycolipid anchor from UDP-glucose. LtaA flips the glycolipid anchor to the outer leaflet. LtaS enzymes then polymerize glycerophosphate (GroP) units derived from phosphatidylglycerol onto the glycolipid anchor, extending the LTA chain at the membrane surface. Both WTA and LTA are modified by d-alanylation through the Dlt pathway: transferring d-alanine to the TA backbone to reduce net negative surface charge and modulate interactions with cationic antimicrobial peptides. Molecular components are color-coded as indicated in the legend. Created in https://BioRender.com.

TAs are critical to the basic cellular architecture and division. In *S. aureus*, TAs direct the positioning of the cell wall synthesis machinery.^138^ LTAs in *S. aureus* help regulate autolytic enzyme activity, preventing excessive self-digestion of the cell wall.^138^ In *S. aureus* and *L. monocytogenes*, WTAs contribute to cell morphology, and their loss results in severe defects in cell shape and division.^141^ In *B. subtilis*, CBPs modulate the activity of autolysins, the enzymes responsible for controlled cell wall turnover.^142^

### 4.1 Biosynthesis of TAs

The biosynthesis of teichoic acids (Figure 5) in monoderm bacteria involves two distinct pathways for WTAs and LTAs, each requiring a dedicated set of enzymatic machinery. The synthesis of WTAs in *S. aureus* proceeds through two major stages that take place on opposite sides of the cytoplasmic membrane. The process begins in the cytoplasm, where a N-acetylglucosamine-1-phosphate transferase (TarO in *S. aureus*) transfers N-acetylglucosamine-1-phosphate onto the lipid carrier undecaprenyl phosphate, forming a membrane-anchored intermediate from which the polymer is subsequently assembled.^143^ TarA and TarB add sugar and ribitol-phosphate units to build the polymer, and the completed chain is exported by the TarGH ABC transporter.^144,145^ Once on the exterior face of the membrane, the WTA polymer is covalently attached to the PG layer through the action of LytR-CpsA-Psr (LCP) (TarTUV), which transfers the completed polymer onto muramic acid residues within the cell wall.^146^

The biosynthesis of LTAs occurs on the outer leaflet of the cytoplasmic membrane. In *S. aureus* and *B. subtilis*, the enzyme LtaS polymerizes glycerol-phosphate units derived from PG directly onto a glycolipid anchor.^147^ In order for LTA polymers to be modified with GlcNAc (or galactose in some species), GlcNAc must be transported to the periplasm. A set of three proteins (a cytoplasmic glycotransferase, a flippase, and a periplasmic glycotransferase) is required.^148,149^ The cytoplasmic glycotransferase utilizes UDP-galactose to form a C_55_–P-galactose, which is transported across the membrane by the flippase. Once in the periplasm, the periplasmic glycotransferase transfers GlcNac to the LTA backbone. This extracellular glycosylation mechanism may also modify the WTA.^150^

Glycosyltransferases add further modifications to the teichoic acid backbone, such as the incorporation of d-alanine through the Dlt pathway, esterification, and the attachment of glycosyl groups. The Dlt pathway, which is widespread among pathogens including *S. aureus*, *L. monocytogenes*, and *Streptococcus pyogenes*, is well characterized.^116,151^ DltA adenylates free d-alanine and loads it onto DltC as a thioester, DltB then transfers it to a tyrosine on DltX, and DltD finally transfers the d-alanine onto the LTA.^152–155^

### 4.2 TAs in Pathogenesis

In *S. aureus*, wall teichoic acids facilitate nasal colonization by interacting with host ligands present on nasal epithelial cells, and animal models in which WTA biosynthesis has been genetically disrupted show dramatically reduced colonization efficiency.^156^ WTAs interact with the scavenger receptor SREC-I expressed on keratinocytes and contributes to staphylococcal skin colonization. Given that nasal and skin carriage of *S. aureus* is a well-established risk factor for invasive infection, this adhesion function carries considerable clinical significance.^157^ The glycotransferase GtcA in *L. monocytogenes* adds galactose residues to WTAs. Strains lacking this modification exhibit reduced virulence in animal models, indicating that the glycosylation modification contributes to pathogenicity.^149,158^ In *Staphylococcus epidermidis*, LTAs contribute to resistance against human β-defensins produced by skin epithelial cells and increase bacterial survival.^159^ In *Enterococcus faecium* and *E. faecalis*, modifications to LTA structure influence the degree to which these molecules stimulate TLR2-mediated inflammatory signaling, with more heavily modified LTAs sometimes evading robust immune detection and allowing the bacteria to persist in host tissues with reduced inflammatory clearance.^160,161^ Teichoic acids also play important roles in modulating complement activation, which is a central pillar of the innate immune response against bacterial pathogens. CBPs (PspA and PspC) in *S. pneuomoniae* lyse human red blood cells during infection.^140^ Another CBP in *S. pneuomoniae*, CbpC, plays a pivotal role in dampening host autophagy during infection, promoting bacterial cell survival.^162^ Finally, CbpG in *S. pneuomoniae* is an adherence factor that also cleaves the host extracellular matrix after secretion.^163^

In response to antimicrobial peptides, bacteria modifiy TAs. d-Alanylation reduces the overall negative charge of the bacterial surface, thereby diminishing the electrostatic attraction between the cell wall and cationic antimicrobial peptides such as defensins and cathelicidins. Thus, d-alanylation provides the bacterium with resistance to a front-line component of the host innate immune system.^164,165^ This antimicrobial resistance can be seen in group A *Streptococcus*, where the loss of the d-alanylation-responsible enzyme DltA results in increased net negative charge at the cell surface and higher susceptibility to antimicrobial peptides.^164^ Finally, d-alanylation of TAs plays a supplementary role in lipoprotein binding to TLR2 during host–pathogen interaction, where the loss of these d-alanylated teichoic acids contributes to a loss in lipoprotein binding.^166^

### 4.3 TA Therapeutic Targets

#### 4.3.1 TA Antibiotic Targets

Teichoic acids and their associated CBPs represent therapeutically attractive targets because of their surface accessibility, essential structural and pathogenic roles, and the absence in mammalian cells. The WTA biosynthetic pathway is particularly rich in potential drug targets because it is dispensable under standard laboratory conditions, yet essential during infection. This distinction implies that WTA pathway inhibitors cause minimal on-target toxicity to commensal bacteria while targeting MRSA and other antibiotic-resistant pathogens.^167^

The WTA biosynthetic pathway has generated sustained interest as an antibiotic adjuvant target, particularly against MRSA. Genetic or pharmacological inactivation of TarO does not kill bacteria outright but abolishes WTA production. Loss of WTA prevents the proper localization of PBP4, the enzyme responsible for highly cross-linked PG. The reduction in crosslinks increases the permeability of antibiotics and resensitizes MRSA to β-lactam antibiotics that would otherwise be ineffective.^167^ Multiple small-molecule TarO inhibitors have been identified, including ticlopidine and the more potent compound tunicamycin. Natural products such as berberine inhibit TarO and the glycosyltransferases TarS and TarM, thereby disrupting WTA glycosylation. These inhibitors restore oxacillin and methicillin susceptibility in both wild-type and mutant MRSA strains.^168^ The ABC transporter TarGH is another validated target, and an inhibitor, Targocil, has been developed. A cryo-EM structure of the TarGH homolog bound to Targocil identified the inhibitor-binding site and a substrate translocation mechanism, providing a framework for structure-guided drug design.^169^ WTA has additionally been exploited as a targeting handle for antibody-antibiotic conjugates. DSTA4637S (Genentech) is a THIOMAB antibody conjugate in which an anti-WTA IgG1 monoclonal antibody is linked via a protease-cleavable linker to the rifamycin-class antibiotic dmDNA31. Phagocytic cells ingest DSTA4637S-opsonized *S. aureus*, and intracellular cathepsins release dmDNA31, which in turn kills the intracellular reservoir of bacteria that conventional antibiotics cannot reach. A Phase 1a study of DSTA4637S in healthy volunteers resulted with a favorable safety and pharmacokinetic profile. However, a subsequent Phase 1b study in patients with complicated *S. aureus* bacteremia revealed infusion-related reactions that led to the discontinuation of the trial.^170,171^

#### 4.3.2 TA Vaccine Targets

The surface exposure of WTA and LTA, combined with their structural roles in bacterial–host recognition, makes them good candidates for vaccine-based strategies. However, their high conservation across commensal and pathogenic species has historically complicated their use as direct antigens. The CBP PspA of *S. pneumoniae* has received the most sustained attention as a vaccine antigen. PspA is present in virtually all clinically relevant *S. pneumoniae* strains and interferes with complement activation by blocking C3b deposition on the bacterial surface and binding lactoferrin to inhibit the alternative pathway.^172^ Recombinant PspA fragments, fusion constructs combining PspA with other CBPs, and nanoparticle-based formulations have all demonstrated strong immunogenicity and protection across multiple animal models, and early phase clinical trials have confirmed safety and immunogenicity in humans. The primary challenge remains antigenic variability across the two major PspA clades and their further subdivisions, which has driven efforts to identify broadly cross-reactive fragments.^172^ LTA itself has been tested as a vaccine antigen in *S. aureus* but has not yielded successful clinical candidates, in part because the adaptive immune response to LTA is T-cell-independent and does not generate durable immunological memory and in part because anti-LTA antibodies have not demonstrated consistent opsonophagocytic killing in preclinical models.^99^

## 5 Overview of the Structure and Organization of the Surface-Layer

Surface (S) layers are crystalline arrays of surface-layer proteins (SLPs) that are typically on the outside of both monoderm and diderm bacteria. SLPs interact with phages, host cells, small molecules, and each other to form biofilms. SLPs self-assemble into two-dimensional crystalline arrays with remarkable diversity of symmetry patterns. SLPs exhibit minimal sequence conservation, even among closely related species, and glycosylation patterns vary considerably across organisms. Some SLPs are nonglycosylated, while others are heavily glycosylated. Many organisms have S-layers that are composed of a mix of nonglycosylated SLPs and glycosylated SLPs with varying glycosylation patterns.

Despite their low sequence conservation, SLPs share recurring structural features at the domain level. Most SLPs are modular proteins composed of two functionally distinct regions: a conserved N-terminal anchoring domain that mediates attachment to the cell surface, and a C-terminal self-assembly domain that drives lattice formation.^173,174^ The self-assembly domain typically adopts an immunoglobulin-like or β-sheet-rich fold, and the stacking of these domains across adjacent monomers generates the regular repeating geometry of the S-layer lattice.^175–177^ The lattices themselves display oblique (p1, p2), square (p4), or hexagonal (p3, p6) symmetry depending on the species, with unit cell dimensions typically ranging from 4 to 35 nm.^174,178^ SLPs range considerably in molecular weight, generally falling between 40 and 200 kDa, and in many organisms the S-layer is composed of a single abundant SLP species, though some bacteria, such as *Bacillus anthracis*, produce heterogeneous arrays containing subunits of distinct sizes and functions.^179,180^ In organisms with heavily glycosylated SLPs, the glycans are attached to serine, threonine, or tyrosine residues and can constitute a substantial fraction of the total molecular mass, influencing both the biophysical properties of the lattice and its interactions with the external environment.^181–184^

SLPs interact non-covalently or attach covalently to a structurally and functionally diverse class of anionic glycopolymers, collectively termed “non-classical” secondary cell wall polysaccharides (SCWPs). Teichoic acids (wall teichoic acids, WTAs, and lipoteichoic acids, LTAs) are considered “classical” SCWPs and are discussed in Section 4. The best-characterized SCWPs are the pyruvylated polysaccharides that serve as anchoring ligands for S-layer homology (SLH)-domain proteins,^185^ but the SCWP family also encompasses a variety of species-specific capsular and quasi-capsular polysaccharides in both monoderm bacteria and diderm bacteria.

SCWPs are heteropolysaccharides covalently attached to the PG layer via muramic acid residues of the PG backbone.^186,187^ Unlike wall teichoic acids, which are largely built from polyol–phosphate repeat units (Section 5.1), SCWPs are composed of neutral and charged sugar residues including hexoses, hexosamines, and uronic acids, arranged in species-specific repeat units of two to six sugars.^188^ The anchoring interaction between SCWPs and SLPs is mediated by dedicated protein domains whose identity varies between organisms, and this variation reflects fundamental differences in cell wall anchoring strategy across bacterial lineages.

In *B. anthracis*, the SCWP is a pyruvylated trisaccharide repeat of ManNAc, GlcNAc, and GalNAc; the ketal pyruvate modification at the 4,6-position of ManNAc is the critical chemical feature recognized by the SLH domains of S-layer proteins Sap and EA1, providing the molecular basis for S-layer anchoring to the cell wall.^185,189^ This SLH-domain/pyruvylated-SCWP interaction is considered an ancestral mechanism for SLP anchoring and is conserved across many SLH-domain-carrying bacteria, including *Geobacillus stearothermophilus* and *Paenibacillus alvei*.^190^

In *Clostridioides difficile*, the anchoring polymer and recognition domain are both distinct from those of the *B. anthracis* system. The predominant anchoring polysaccharide is polysaccharide II (PSII), a hexaglycosyl phosphate repeat, which interacts with the cell wall binding domain 2 (CWB2). CWB2 is a structurally unrelated SLP.^190^ PSII attachment to PG is mediated by LCP enzymes LcpA and LcpB, and this attachment is essential for bacterial viability.^191^ The S-protein organization and anchoring diversity extend further to deep-branching extremophiles such as *Thermus thermophilus* and *Deinococcus radiodurans*, which possess S-layers positioned outside an atypical OM that lacks lipopolysaccharides and interact with both the OM and PG through distinct mechanisms.^192^

### 5.1 S-Layer Biogenesis

S-Layer biogenesis requires the coordinated execution of two distinct but interdependent biosynthetic programs: the production and surface display of the S-layer protein itself and the synthesis and cell wall attachment of the secondary cell wall polysaccharide (SCWP) that anchors it. In organisms that use SLH domain-mediated anchoring, an SLP secreted in the absence of its cognate pyruvylated SCWP fails to associate stably with the cell surface, and conversely, a properly assembled SCWP without its SLP partner provides no protective lattice. The pathways differ substantially between monoderm and diderm bacteria, reflecting the structural differences in their cell envelopes, but in both cases, the result is the same: a self-assembled crystalline protein array precisely positioned at the outermost surface of the cell. The following subsections describe the biogenesis of SCWPs and S-layer proteins in turn with particular emphasis on the molecular mechanisms of secretion, modification, anchoring, and lattice assembly.

#### 5.1.1 SCWP Biogenesis

SCWP biosynthesis (Figure 6) in *B. anthracis* initiates with TagO, a UDP-*N*-acetylglucosamine:undecaprenyl-phosphate transferase that generates the lipid-linked murein linkage unit onto which the SCWP trisaccharide repeat is built.^189,193^ The trisaccharide repeat unit [→4)-β-ManNAc-(1→4)-β-GlcNAc-(1→6)-α-GlcNAc-(1→] requires dedicated glycosyltransferases for its assembly, including the products of *wpaA* and *wpaB*, which are proposed to polymerize repeat units or transfer the completed polymer to the linkage unit prior to PG attachment.^194^ CsaB, a ketal pyruvyltransferase encoded within the conserved S-layer gene cluster, modifies the SCWP with a pyruvate group; this modification is the critical chemical feature recognized by the SLH domains of Sap and EA1, and loss of CsaB abolishes S-layer anchoring entirely.^185^ Following assembly and modification, mature SCWP chains are transferred from the lipid carrier to the C6-hydroxyl of MurNAc residues in PG by LCP family enzymes, the same enzymes that attach wall teichoic acids to PG (Section 4.1) and capsular polysaccharides (CPS) in monoderm bacteria (Section 8.1). *B. anthracis* encodes six LCP paralogs with partially overlapping but distinct roles in SCWP deposition, vegetative growth, and sporulation.^195,196^ Additional modifications including galactosylation of the trisaccharide backbone contribute to proper cell wall envelope assembly and are required for full virulence.^197^

**Figure 6:**
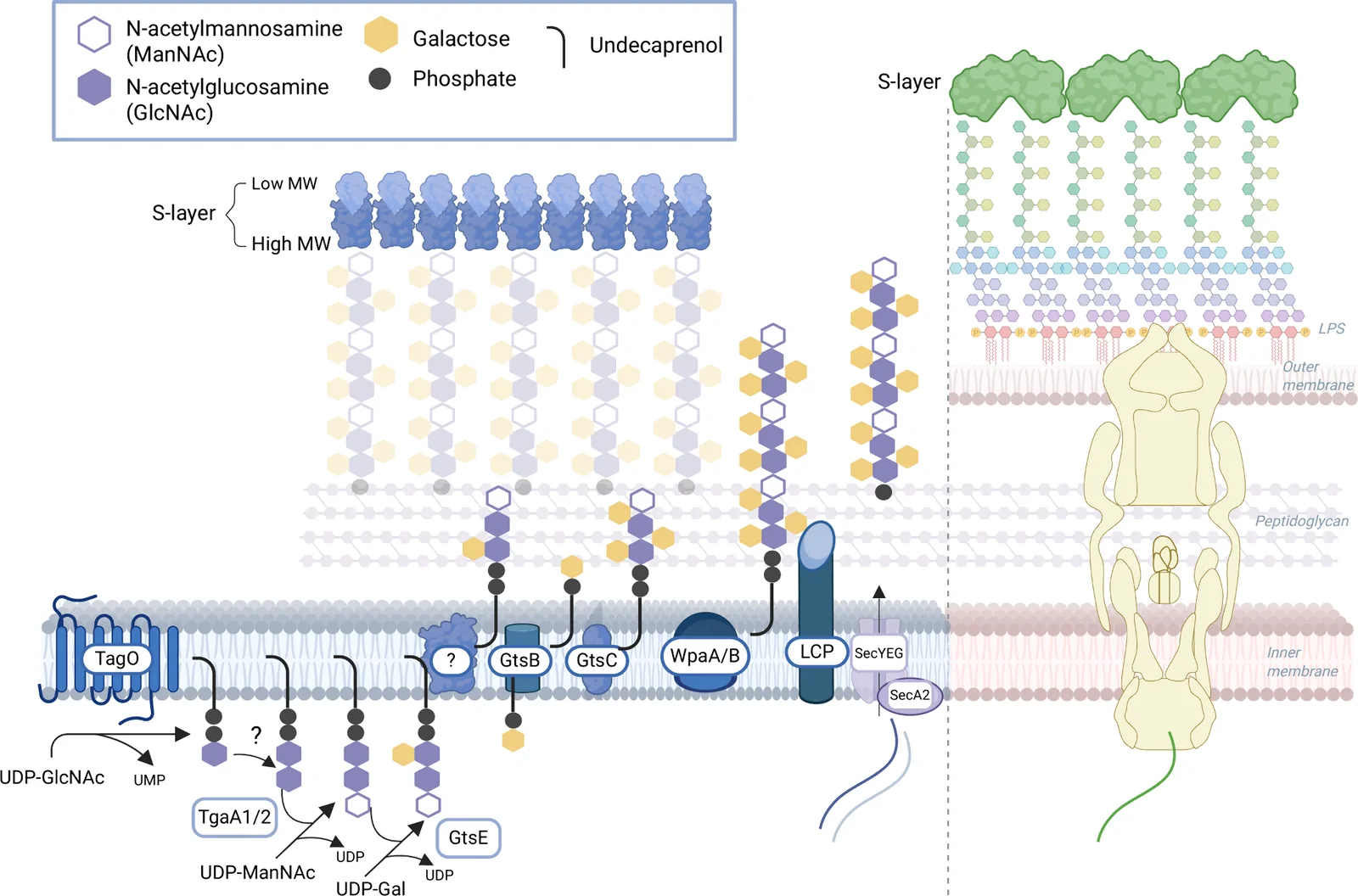
**S-Layer biogenesis and assembly in bacteria.** (Left) SCWP biosynthesis and S-layer assembly in *B. anthracis* as a monoderm model. SCWP synthesis is initiated at the IM by TagO, which transfers GlcNAc-1-phosphate from UDP-GlcNAc onto the undecaprenyl phosphate lipid carrier, releasing UMP. The identity of the glycotranferase for adding the second GlcNAc is unknown (?). TgaA1/2 then add a ManNAc from UDP-ManNAc releasing UDP. GtsE adds galactose from UDP-Gal, and an unknown membrane-associated transporter flips the modified undecaprenyl to the outer leaflet of the IM. GtsB transports a galactose modified undecaprenyl phosphate lipid to the outer leaflet of the IM. GtsC catalyzes galactose modification of the pentasaccharide. WpaA/B polymerizes the polysaccharide. An LCP enzyme then transfers the mature SCWP from the lipid carrier onto MurNAc residues of the PG layer. S-layer proteins (blue/light blue) are secreted across the IM by the Sec translocon/SecA2 complex and self-assemble on the cell surface into a crystalline array composed of heterogeneous low molecular weight and high molecular weight subunits. (Right) S-layer assembly in *Aeromonas salmonicida* and *Aeromonas hydrophila* as diderm models. In these organisms, the S-layer proteins (green) assemble on the OM surface, anchored to LPS via non-covalent protein–carbohydrate interactions. The S-layer protein is secreted across both membranes by a T2SS (yellow, see Section 10). Molecular components are color-coded as indicated in the legend. Created in https://BioRender.com and adapted from Figure 2 in^173^ and Figure 1 in.^921^ Figure 2 in ref 173 is available under a CC-BY 4.0 license (Copyright 2014 Fagan and Fairweather); Figure 1 in ref 921 is reproduced with permission (Copyright 2022 American Society for Microbiology).

The overall logic of the *B. anthracis* pathway (lipid-carrier-mediated repeat unit assembly, pyruvylation, LCP-dependent PG ligation, and SLH-domain recognition) is conserved as a general strategy among SLH-domain-carrying bacteria, although the specific polysaccharide structures and modifying enzymes vary considerably between species. In *G. stearothermophilus* and related thermophilic bacilli, the SCWP is similarly pyruvylated and the CsaB orthologue is required for pyruvylation and S-layer anchoring.^185^ In *P. alvei*, the SLH domains of the glycosylated S-layer protein SpaA bind to pyruvylated SCWP and the PG directly, which provides additional anchoring not observed in *B. anthracis*.^198^

#### 5.1.2 S-Protein Biogenesis

In monoderm species such as *B. anthracis* and *C. difficile*, S-layer precursors are secreted via accessory Sec systems involving SecA2 and SecY2, with signal peptides cleaved upon membrane translocation (Figure 6).^199–201^ Diderm bacteria use secretion systems; however, different processes are utilized depending on the species.^202–204^ Following secretion, proteins anchor to the cell wall through distinct mechanisms: monoderm SLPs have SLH-domains that bind secondary cell wall polymers such as pyruvylated polysaccharides, while diderm SLPs associate with LPS via protein–carbohydrate interactions.^205–207^ Recent cryotomography studies reveal that S-layer assembly is spatially coordinated with PG synthesis and cell division, with new proteins incorporating at poles and division sites where lattice discontinuities facilitate insertion.^208^ Some species such as *C. difficile* require proteolytic processing by cysteine proteases (Cwp84) for proper S-layer maturation and assembly.^209^ Some bacteria change the S-layer in response to environmental conditions or to evade the host immune system. For example, *B. anthracis* expresses switchable SLPs (Sap and EA1), with Sap produced during exponential growth and EA1 during the stationary phase.^179^ Campylobacter fetus employs antigenic variation of S-layer proteins to evade adaptive immunity during chronic infections.^210^

### 5.2 S-Layer in Pathogenesis

SLPs are critical virulence factors mediating pathogen–host recognition and immune modulation through multiple mechanisms.^211,212^ In *C. difficile*, the predominant S-layer protein SlpA contains high and low molecular weight subunits. The high molecular weight subunit mediates adhesion to intestinal epithelial cells and extracellular matrix components and, thus contributes to colonization.^213,214^ The intact S-layer induces both pro-inflammatory cytokines (IL-1β, TNF-α) and regulatory cytokines (IL-10) through interactions with pattern recognition receptors including TLR2 and dendritic cell-specific ICAM-3-grabbing non-integrin (DC-SIGN).^212,213,215^ In *Tannerella forsythia*, an oral pathogen, S-layer deficiency abolishes adherence to gingival epithelial cells, demonstrating the essential role in colonization.^216^ Conversely, probiotic *Lactobacillus* species utilize S-layers for beneficial host interactions, with *Lactobacilus acidophilus* S-layer proteins blocking pathogen adhesion by occupying DC-SIGN binding sites phenotypes.^217^

Additionally, the cell envelope of *C. difficile* features a prominent S-layer composed of SLPs that mediate host cell adhesion and immune evasion, contributing to its pathogenicity.^190,218,219^ This proteinaceous outer layer is essential for colonization and toxin delivery, which underscores the diverse strategies bacteria employ to establish infection.^190,220^

### 5.3 S-Layer Therapeutic Targets

#### 5.3.1 S-Layer Antibiotic Targets

S-Layers are the outermost bacterial surface in many Gram-positive pathogens and are accordingly exposed to host immune surveillance and potential therapeutic agents. Their lineage-specific composition, high abundance, and essential roles in adhesion, immune evasion, and cell envelope integrity make them attractive targets for both antibiotic adjuvant strategies and vaccine development.^190^ The SLH-SCWP anchoring interface represents a discrete, chemically accessible point of vulnerability. Disruption of this interaction would collapse the S-layer assembly without requiring intracellular drug penetration.^221^

Despite the structural rationale, no commercially available antibiotics that directly target S-layer proteins (SLPs) or SCWPs are currently available. The SLH–SCWP interface has been proposed as a drug target based on crystallographic evidence that the terminal pyruvylated ManNAc moiety of the SCWP serves as the nearly exclusive anchoring point for the SLH domain trimer of *P. alvei* SpaA. The co-crystal structures define two distinct binding sites and show that engagement of a single pyruvylated monosaccharide is sufficient to trigger receptor rearrangement, suggesting that small molecules or synthetic SCWP analogs designed to block or mimic this interaction could prevent S-layer assembly.^221^ The broader appeal of the S-layer as a drug target is further supported by work in *C. difficile*, where SlpA mediates epithelial adhesion and tight junction disruption, functions essential to colonization. Thus, pharmacological interference with S-layer assembly or surface presentation could reduce virulence independently of toxin production.^212^ However, antibiotic development targeting the S-layer remains at the structural biology and target validation stage; no inhibitor series targeting this interface has yet been reported in clinical or late preclinical literature.

#### 5.3.2 S-Layer Vaccine Targets

S-Layer proteins have a more developed translational record as vaccine antigens than as drug targets, primarily in the context of *C. difficile* and *B. anthracis*. In *C. difficile*, the predominant S-layer protein SlpA has been proposed as a surface antigen component to complement toxoid-based vaccine formulations.^212^ The *C. difficile* vaccine landscape has been shaped by the Phase 3 CLOVER trial (NCT03090191), which tested Pfizer’s detoxified toxin A/B vaccine PF-06425090 in 17 535 adults aged 50 years or older at increased risk of CDI. The primary efficacy end point was not met, although the vaccine significantly reduced CDI symptom duration, the need for medical attention, and antibiotic use among those who developed CDI.^222^ This failure of a toxin-focused approach has intensified interest in surface protein antigens, including SlpA, as components of next-generation multicomponent *C. difficile* vaccines.^223^ In *B. anthracis*, both EA1 and the SCWP have been investigated as supplementary antigens in recombinant anthrax vaccine formulations to broaden immune coverage beyond the current protective antigen (PA)-based approach, although no S-layer-based anthrax vaccine has advanced to clinical trials.^190^ Across both pathogens, the central challenge for S-layer-based vaccines is antigenic variation. *C. difficile* undergoes phase-variable S-layer switching between different SLP variants, and *B. anthracis* switches between Sap and EA1 depending on the growth phase, both of which could reduce the breadth of S-layer-directed immune responses.^190^

## 6 Overview of the Components and Organization of the OM

The OM is composed of LPS, lipids, lipoproteins, and integral OMPs (Figure 7), and typically is attached to the outer surface of the PG.^113^ The composition and complexity of the OM varies significantly. For instance, *Thermotoga maritima* has a detached OM (at the poles) with lipid and protein compositions that deviate from those of other diderm bacteria.^224^ This asymmetrical OM has glycerophospholipids in the inner leaflet and LPS in the outer leaflet.^225^ The outermost LPS layer is essential for structural integrity and mediates interactions with the host environment.^226^ Strong lateral interactions among LPS monomers are mediated by divalent cations, which boost the cell barrier’s impermeability.^101,227^ The OM also contains lipoprotein complexes and β-barrel OMPs that support nutrient uptake and protein trafficking while maintaining the barrier.^218,227^

**Figure 7:**
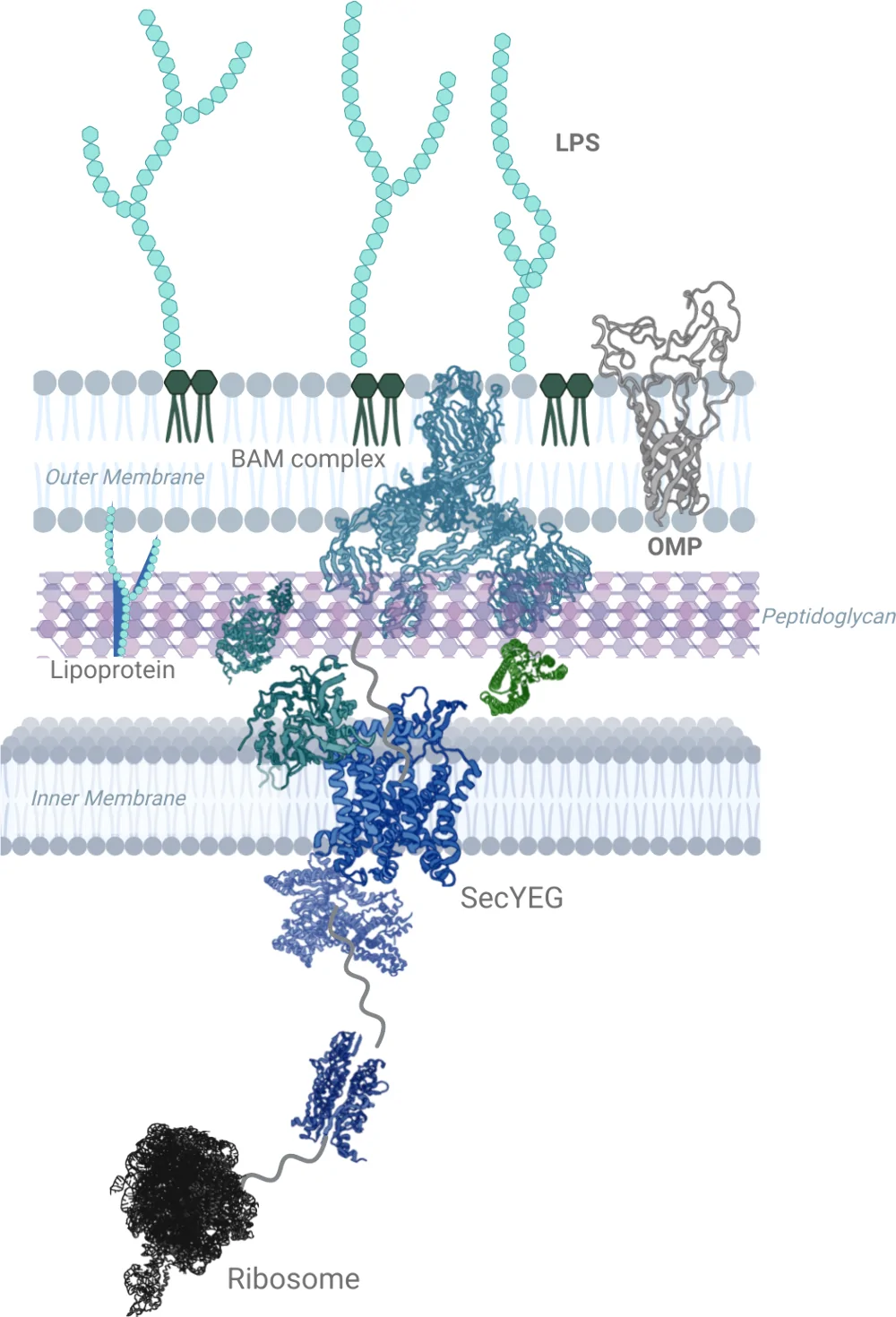
**OMP biogenesis pathway in Diderm bacteria.** OMPs are synthesized by cytoplasmic ribosomes (black, intracellular) and translocated across the IM via the Sec translocon (blue, IM) as unfolded polypeptides. In the periplasmic space, nascent OMPs are bound by chaperones (shown in green and teal) that prevent aggregation and premature folding while escorting them through the aqueous periplasm. The chaperone-OMP complex is delivered to the β-barrel assembly machinery (BAM complex, cyan) at the OM, a multi-subunit protein complex that catalyzes the folding and insertion of β-barrel OMPs into the OM. Once properly inserted, OMPs (light gray) adopt their characteristic β-barrel structure and perform diverse functions, including nutrient transport, signaling, and structural roles. The OM also contains lipopolysaccharide (LPS) on its outer leaflet and lipoproteins that connect the OM to the PG layer. This coordinated pathway ensures proper localization and folding of OMPs, which are essential for Diderm bacterial cell envelope integrity. Created in https://BioRender.com.

### 6.1 OM Lipid Composition and Structure

The asymmetry is actively maintained by transport systems such as the Mla pathway.^228^ A complementary quality control mechanism is provided by the OM phospholipase PldA (also known as OmpLA), which degrades phospholipids that accumulate in the outer leaflet and, thereby, directly removes the lipid asymmetry defect at its source.^229^ PldA also functions as a sensor of outer leaflet disruption, generating fatty acid second messengers that signal the cell to upregulate LPS biosynthesis and restore homeostasis.^230^

LPS consists of three domains: the multifunctional lipid A endotoxin embedded in the outer leaflet; the core oligosaccharide of approximately 8–12 sugar residues, comprising a conserved inner core of Kdo and heptose residues covalently linked to lipid A, and a more variable outer core; and the O-antigen, a polysaccharide composed of 3-5 sugars, repeating up to 25 times.^231,232^ While most OMs of diderm bacteria are composed of LPS, *N. gonorrhoeae*, *Neisseria meningitidis*, and *Haemophilus ducreyi* instead possess lipooligosaccharide (LOS). LOS closely parallels LPS in lipid A structure and overall function but lacks the O-antigen.^233^ Some diderm bacteria lack LPS entirely. *B. burgdorferi* (Spirochetes) lacks LPS in its OM and instead populates its outer surface with an abundance of surface-exposed lipoproteins.^234^ *Sphingomonas* species similarly lack LPS but replace it with glycosphingolipids in the outer leaflet of the OM, demonstrating that the canonical LPS composition of the diderm OM is subject to evolutionary modification.^235^

#### 6.1.1 OMP Structure

OMPs are antiparallel β-sheet that closes into a barrel, with the sequence of each strand alternating hydrophobic and hydrophilic residues. This alternating pattern, with hydrophobic residues facing the lipid environment and hydrophilic residues lining the barrel interior or forming interstrand hydrogen bonds, is essential for membrane insertion and stability.^236–238^ Aromatic residues form prominent ’aromatic belts’ at the membrane interfaces.^239^ These aromatic residues preferentially partition at the membrane-water interface, anchoring the barrel at the correct depth in the membrane and providing thermodynamic stability.^240^ The positioning of aromatic belts is remarkably conserved across diverse OMPs, and the distance between the extracellular and periplasmic belts correlates with the hydrophobic thickness of the OM (∼25-27 Å, thinner than the IM).^241^ The number of β-strands in bacterial OMPs typically ranges from 8 to 36, with the barrel diameter determined by strand and shear number.^242^ Strand tilt relative to the barrel axis averages 40-50, optimizing hydrogen bonding between strands and the overall barrel stability. Many OMPs have a ’β-signal’ at the C-terminus, typically ending with an aromatic residue and a polar or charged residue in the penultimate position, while the -3 position contains an aromatic residue, which is important for recognition by the BAM complex. That mediates the insertion of OMPs into the OM.^243^

### 6.2 OM Biogenesis

Building and maintaining the OM therefore requires the coordinated activity of at least four distinct systems: the LPS synthesis and transport machinery that establishes outer leaflet asymmetry, the Sec and BAM machineries that translocate, fold and insert β-barrel OMPs, the LPS-OMP interaction network that organizes the mature membrane into protective structure, the Lol pathway that traffics lipoproteins to their final destinations, and the transport of glycerophospholipids to the inner leaflet of the OM to maintain asymmetry that helps prevent influx of toxins. The following subsections describe each of these processes.

#### 6.2.1 LPS Biogenesis

LPS biosynthesis is a multi-stage process that proceeds from the cytoplasm to the OM outer leaflet and can be divided into three sequential phases: synthesis of lipid A and its glycosylation with Kdo, assembly and attachment of the core oligosaccharide and O-antigen, and transport across the cell envelope via the Lpt machinery.

Lipid A biosynthesis proceeds through a conserved nine-enzyme pathway at the cytoplasmic face of the IM, known as the Raetz pathway.^244,245^ The first committed step is the deacetylation of UDP-3-O-(R-3-hydroxymyristoyl)-N-acetylglucosamine by LpxC, which is tightly post-translationally controlled and serves as the primary regulatory checkpoint for LPS synthesis.^244^ Sequential reactions catalyzed by LpxD, LpxH, LpxB, and LpxK generate the lipid IV_A_ intermediate, to which two 3-deoxy-D-*manno*-oct-2-ulosonic acid (Kdo) residues are added by the bifunctional enzyme KdtA. Addition of the secondary acyl chains then produces the mature hexa-acylated Kdo_2_-lipid A endotoxin moiety embedded in the inner leaflet of the IM.^244,245^ Core oligosaccharide assembly is completed on the cytoplasmic face by sequential glycosyltransferases, generating the lipid A core (rough LPS, R-LPS).^245,246^

In smooth LPS (S-LPS)-producing bacteria, the O-antigen polysaccharide is synthesized separately. In the widely distributed Wzx/Wzy-dependent pathway, repeat units are assembled on an undecaprenyl phosphate (Und-P) lipid carrier at the cytoplasmic face of the IM, flipped to the periplasmic face by the Wzx flippase, polymerized by Wzy, and then ligated to the completed lipid A core by the WaaL O-antigen ligase.^245,246^ In the ABC transporter-dependent pathway, used by organisms such as *Brucella abortus*, the O-antigen polymer is assembled on Und-PP and translocated by the Wzm/Wzt ABC transporter complex.^247,248^

Regardless of the O-antigen assembly route, the completed R-LPS or S-LPS must be flipped from the inner to the outer leaflet of the IM by the essential ABC transporter MsbA before it can be exported.^249^ Cryo-EM structures of MsbA in multiple functional states show that LPS binds deep within the transporter cavity, establishing extensive hydrophilic and hydrophobic contacts, and that ATP-driven transitions from inward- to outward-facing conformations catalyze translocation.^250^ In unipolarly growing bacteria such as *B. abortus*, MsbA localizes preferentially to the old cell pole, while the downstream Lpt complex concentrates at the new pole, implying that LPS undergoes lateral diffusion in the IM before reaching the export machinery.^248^

Once in the periplasmic leaflet of the IM, LPS is extracted and transported to the OM by the seven-component Lpt system, which forms a continuous transenvelope bridge.^226^ The IM ABC transporter LptB∼2∼FGC uses ATP hydrolysis to extract LPS from the membrane and powers its movement through the periplasmic carrier LptA, which relays it to the OM translocon formed by LptD and LptE.^226^ LptD is a 26-stranded β-barrel through whose lateral gate LPS is inserted into the outer leaflet of the OM; crystal structures of LptD/LptE complexes with bound LPS have directly visualized this insertion step and the role of the lateral gate.^251^ The unidirectional, energy-dependent nature of LPS biogenesis maintains the characteristic lipid asymmetry of the OM that is essential for the impermeability barrier.

#### 6.2.2 OMP Biogenesis and Folding

OMP biogenesis starts with translocation across the IM via the Sec pathway, with signal peptides (SPs) directing them to SecYEG (Figure 7).^231^ The N-terminal SP sequence facilitates their translocation across the IM.^252^ Once the SP is recognized and processed, periplasmic chaperones SurA, Skp, FkpA, and DegP prevent aggregation and premature folding of OMPs in the aqueous periplasm (Figure 7).^253–255^ SurA is the primary periplasmic chaperone, recognizing unfolded OMPs through the conserved hydrophobic motif (ϕXϕϕPro) via its peptidylprolyl isomerase (PPIase) domains which maintain them in a folding-competent state and facilitate their folding.^256^ SurA escorts OMPs, while Skp forms a trimer that encapsulates OMPs to promote the early stages of OMP assembly.^253,257–259^ Skp also acts as a quality control chaperone, directing the removal of stalled OMPs that have undergone incomplete folding for degradation by DegP.^253,260^ FkpA responds to heat-shock stress and, unlike other periplasmic chaperones, increases the folded yield but decreases the folding rate of OMPs.^261^ DegP provides both chaperone and protease activities depending on temperature and cellular stress.^262,263^ The absence of SurA results in slower translocation of the protein to the BAM complex. The absence of Skp facilitates direct binding of SurA to the protein.^264–266^ The conserved ‘β-signal’ motif (GXXϕXϕ) located at the C-terminus of OMPs is an important recognition determinant for the BAM complex, including the essential accessory lipoprotein BamD.^264,267–269^ BamA, the core component of the BAM complex, contains N-terminal periplasmic polypeptide transport-associated (POTRA) domains that interact with unfolded OMPs delivered by periplasmic chaperones and coordinate their assembly.^264,269,270^ The accessory lipoproteins BamBCDE contribute to the stability, organization, and regulation of the BAM complex, modulating BamA conformational dynamics and substrate assembly rather than directly mediating the transfer of unfolded OMPs into the BamA β-barrel lumen.^271–273^ Together, these components stabilize the complex and facilitate efficient transfer and folding of unfolded OMPs into the lumen of the C-terminal β-barrel BamA.^264,269,270,274^ OMPs that lack the β-signal may exhibit impaired BAM recognition and consequently remain in the periplasm or undergo degradation.^264^ Differences in the β-signal sequence appear to be species- and clade-specific, suggesting an adaptation between the BAM complex and nascent OMP substrates.^264,265,275^ These chaperones work synergistically, with partially redundant functions ensuring robust OMP delivery.^255^

The final step of OMP biogenesis occurs at the BAM complex (Figure 7). In *E. coli*, BamA, a 16-stranded β-barrel with five N-terminal periplasmic polypeptide transport-associated (POTRA) domains interact with unfolded substrates from periplasmic chaperones.^218,264,276^ Several models have been proposed to explain how the BAM complex coordinates OMP assembly into the OM, and these models may represent complementary mechanisms rather than mutually exclusive pathways. The “budding” or “threading” model proposes that BamA transiently accommodates unfolded OMPs within its lumen, where the substrate progressively folds into β-hairpins that are subsequently released or “bud” laterally through the BamA barrel to assemble into the mature β-barrel.^277,278^ In contrast, the “BAM-assisted“ model proposes that the complex functions primarily as a chaperone-like catalyst that locally destabilizes the membrane environment, thereby lowering the energetic barrier for spontaneous OMP folding and insertion.^274,278^ Both models emphasize the dynamic nature of BamA and the importance of the surrounding lipid environment in facilitating OMP assembly.^274,277,278^

Structural and biochemical studies have proposed additional models that describe specific stages of this process. The “swing” model proposes that OMP engagement begins through an interaction between the substrate’s C-terminal β-signal and the first β-strand of BamA, forming an inter-β-barrel seam. Subsequent conformational changes in BamA promote a swinging motion of the partially assembled OMP away from the BamA barrel and into the surrounding membrane, where β-strand augmentation and progressive folding can occur.^278^ Related “β-signal exchange” and “β-barrel switching” models emphasize the transient formation of a hybrid β-barrel between BamA and the incoming OMP. In these models, β-strand interactions between the substrate β-signal and BamA initiate assembly, followed by progressive β-strand exchange that allows the substrate to displace BamA and form an independent β-barrel.^279^ Rather than representing entirely distinct mechanisms, these models provide complementary frameworks for understanding how β-signal recognition, BamA conformational dynamics, OMP folding, and membrane insertion may be coordinated during OMP assembly.

Experimental evidence further supports an important role for β-signal recognition during the early stages of BAM-mediated assembly. In *E. coli*, the small molecule antibiotic darobactin inhibits binding of the autotransporter EspP β-signal to BamA and prevents subsequent OMP assembly, whereas other BAM-targeting compounds, including Polyphor peptide 7 and MRL-494, interfere with substrate binding and subsequent stages of assembly.^280–282^ These findings support a model in which β-signal engagement with BamA initiates membrane insertion and BAM-mediated assembly proceeds through multiple mechanistically distinct stages. Collectively, current structural and biochemical evidence supports a dynamic model in which BamA transiently engages nascent OMPs through their β-signal, undergoes conformational rearrangements that facilitate substrate insertion, and coordinates progressive β-barrel folding with release into the OM. The relative contributions of threading, membrane-assisted, swing, and strand-exchange mechanisms may vary among OMP substrates and their respective folding pathways, rather than reflecting a single universal assembly mechanism for all OMPs.^278,280–282^

Atypical OMs, such as those in *T. maritima*, other *Thermotogae*, *Spirochaetes*, and *Bacteroidetes* have unusual and unknown biogenesis pathways. In *T. maritima* and other *Thermotogae*, the detached toga membrane requires specialized trafficking mechanisms that involve vesicular transport and distinct lipid compositions.^224,283^ In *Treponema pallidum*, Tp92, also known as Tp0326 (BamA), is evolutionarily distinct from that found in other diderm bacteria, which suggests the OMPs may also be atypical.^284,285^ The *T. pallidum* BAM complex is larger than *E. coli* (∼300 to 400 kDa versus 232 kDa) and lacks BamBCDE accessory lipoprotein subunits.^284^ Moreover, *T. pallidum* BamA has a positively charged lumen and an unusual extracellular loop containing polyserine-rich sequences found only in pathogenic treponemes.^284^ Unlike typical diderm bacteria, the OM of *T. pallidum* is thought to be fragile and deficient in OMPs and is, thus, more susceptible to disruption by detergents, even at relatively low concentrations.^284^ Similarly, the *Flavobacterium johnsoniae* BAM complex structure is different than the canonical *E. coli* BAM complex.^286,287^ *F. johnsoniae* BAM complex contains an extracellular region (composed of BamG and H) that extends over the substrate folding site, as well as an additional subunit that inserts into the BamA pore.^286,287^

Importantly, atypical OMP architecture is not restricted to variations in BAM composition or biogenesis pathways. Although most characterized OMPs form individual β-barrels, structural and bioinformatic analyses have identified OMPs containing multiple β-barrel domains within a single polypeptide, expanding the known architectural diversity of OM β-barrels.^288^ Other OMPs form higher order oligomeric assemblies in which multiple small β-barrel subunits collectively generate a larger membrane-spanning pore. For example, the *E. coli* curli secretion protein CsgG comprises nine approximately four-stranded protomers that assemble into a 36-stranded β-barrel secretion channel.^289–291^ More recently, the pentameric PopA from the predatory bacterium *Bdellovibrio bacteriovorous* was shown to form an unusual porin-like oligomeric architecture associated with lipid trapping, further illustrating the structural diversity of β-barrel OMP assemblies.^292^ Conversely, some OMPs depart entirely from the canonical β-barrel fold. The *E. coli* polysaccharide export protein Wza, for example, forms a large α-helical transmembrane barrel with a central cavity that accommodates the passage of assembled capsular polysaccharide.^293^ Collectively, these examples demonstrate that OMPs encompass a broader range of architectures than the canonical single β-barrel paradigm, including multibarrel proteins, oligomeric β-barrel assemblies, and entirely α-helical transmembrane structures. This structural diversity likely necessitates corresponding adaptations in the pathways responsible for OMP targeting, assembly, and function across diverse diderm bacterial lineages.

#### 6.2.3 LPS-OMP Interactions and Organization

Direct interactions between OMPs and LPS have been identified in several systems. In *E. coli*, interactions between the OmpC porin and LPS have been proposed to facilitate OmpC insertion into the OM, suggesting that LPS contributes to both OMP biogenesis and OM organization.^294–296^ The ferrichrome receptor FhuA (a 22-stranded β-barrel) co-crystallized with a bound LPS molecule.^297^ The FhuA-LPS contacts occur primarily through electrostatic interactions between the phosphate residues of lipid A and Kdo I and II sugar moieties and basic, positively charged amino acids of FhuA.^297^ This FhuA structure revealed a conserved LPS-binding motif consisting of four basic amino acids, which was identified in other prokaryotic and eukaryotic LPS-binding proteins.^298^ Solution NMR spectroscopy demonstrated that *P. aeruginosa* OprH directly binds LPS through predominantly electrostatic interactions localized to extracellular loops 2 and 3.^299,300^ The OM protease OmpT requires LPS as an essential cofactor for catalytic activity; the crystal structure identifies a putative LPS-binding site containing conserved basic residues (Arg138, Arg175, Lys226).^301,302^

LPS forms a tightly integrated supramolecular network with OMPs. Molecular dynamics simulations reveal that each OMP has a unique pattern of interactions with surrounding LPS molecules, determined by the protein’s composition, extracellular domain architecture, and positioning of residues at the membrane interface (referred to as “LPS fingerprint”).^303,303,304^ Both OMPs and LPS molecules are proposed to have limited lateral mobility once inserted into the OM. Furthermore, the OMPs and LPS segregate into clusters, forming an ordered protein–lipid lattice that provides structural integrity and mechanical strength.^305,306^ OmpA plays a critical role in organizing this lattice by remaining immobile through anchoring to the PG and strongly interacting with surrounding OMPs via LPS bridges.^305^

#### 6.2.4 OM Lipoprotein Biogenesis

OM lipoproteins have diverse cellular functions, and many are virulence factors.^307,308^ Lipoproteins are matured through a post-translational lipoprotein-processing pathway in the IM as described in Section 2 (Figure 2). Following translocation across the IM, lipidation, and signal sequence cleavage, lipoproteins that lack IM retention signals (an Asp residue in the 2 position) are selectively transported to the OM through the localization of lipoproteins (Lol) pathway (Figure 2).^309^ This process is initiated by the ABC transporter complex LolCDE, embedded in the IM. Powered by ATP hydrolysis, LolCDE extracts mature lipoproteins from the IM and transfers them to the periplasmic chaperone LolA.^307^ LolA shields the hydrophobic lipid moieties of lipoproteins and transports them across the aqueous periplasm.^307,310^ The LolA-lipoprotein complex subsequently interacts with LolB, an OM receptor that facilitates lipoprotein insertion into the inner leaflet of the OM. LolB exhibits a higher affinity for lipoproteins than LolA, promoting an efficient transfer and release of the substrate.^307,310^ Although LolA and LolB share structurally similar hydrophobic cavities, differences in amino acid composition contribute to their differential binding affinities and functional roles.^311,312^

In addition to lipoprotein trafficking, the OM must maintain a highly asymmetric lipid composition in which glycerophospholipids are predominantly restricted to the inner leaflet.^228,313^ The maintenance of this asymmetry is mediated in part by the maintenance of lipid asymmetry (Mla) pathway, which spans the IM, periplasm, and OM.^313^ The Mla system comprises the IM ABC transporter MlaFEDB, the periplasmic lipid-binding protein MlaC, and the OM lipoprotein MlaA, which associates with OmpC or OmpF.^314–316^ Current evidence supports a primary role for Mla in retrograde glycerophospholipid transport, whereby mislocalized glycerophospholipids are extracted from the OM by MlaA, transferred to MlaC, and shuttled across the periplasm to MlaFEDB for reinsertion into the IM.^314,316,317^ Structural and biochemical studies have provided mechanistic insight into this process, demonstrating that MlaFEDB can bind and translocate phospholipids through its central cavity.^315,316^ Although Mla-mediated anterograde glycerophospholipid transport from the IM toward the OM has also been proposed, the physiological directionality and contribution of this activity remain debated. Thus, Mla is best characterized as a lipid trafficking pathway that helps maintain OM lipid asymmetry by removing and recycling aberrantly localized glycerophospholipids.^314,316^

Following insertion into the inner leaflet of the OM by LolB, a subset of lipoproteins are translocated to the outer leaflet or cell surface through dedicated secretion pathways, including the surface lipoprotein assembly modulator (Slam), type II secretion system (T2SS), and the type V secretion system (T5SS) (see Section 10).^318–320^ For example, the pullulanase lipoprotein from *Klebsiella oxytoca* is a substrate of T2SS, whereas the lipidated autotransporter NalP from *N. meningitidis* is secreted through the T5SS.^321,322^ Together, these pathways illustrate the diverse mechanisms by which diderm bacteria traffic lipoproteins from their site of synthesis at the IM to distinct destinations within or beyond the OM.

### 6.3 OM in Pathogenesis

#### 6.3.1 OMPs in Pathogenesis

The OM of diderm bacteria is a crucial permeability barrier that protects against components of the innate immune response, antibiotics, and other antimicrobials.^218,227^ β-Barrel OMPs are key for the membrane’s integrity and selective permeability. They perform several essential tasks, including membrane biogenesis, cell signaling, protein import and export, and the transport of toxins and metabolites. In addition, they act as virulence factors in pathogenic bacterial strains.^231^ OMPs in diderm bacteria are crucial for survival and pathogenicity.^220^ The OmpA family has several different functions and provides structural stability, connecting the OM to the PG layer via interactions with Braun’s lipoprotein.^305,323^ OmpA has an unusual domain organization in which an N-terminal eight-stranded β-barrel spans the OM and a C-terminal periplasmic domain binds PG, making it one of the few OMPs with a large functional domain on the periplasmic face.^324^ Without OmpA, the OM loses mechanical cohesion, making the bacteria more susceptible to environmental stress and potentially affecting their ability to cause infections.^305,324^ OmpA also contributes to host–pathogen interactions through both direct and indirect mechanisms. *E. coli* K1 OmpA mediates invasion of brain microvascular endothelial cells to facilitate meningitis,^325^ *Klebsiella pneumoniae* OmpA attenuates immune evasion *in vitro* and *in vivo;* however, these effects appear to involve downstream host signaling and bacterial defense mechanisms rather than a single defined direct receptor-mediated interaction.^326^ Enterohemorrhagic *E. coli* O157:H7 OmpA induces dendritic cell cytokine production, although the contribution of other bacterial surface components to this response cannot be excluded.^327^ Collectively, these findings indicate that OmpA influences pathogenesis through a combination of direct interactions with host cells and indirect effects arising from its structural and regulatory roles in the OM.

Beyond the OmpA family, other OMPs mediate host interactions. The multivalent adhesion molecule (MAM) binds fibronectin and phosphatidic acid on host membranes, thereby stabilizing attachment.^328^ The phase-variable opacity-associated (Opa) proteins of *N. gonorrhoeae* and *N. meningitidis* are 8-stranded β-barrel proteins that bind to carcinoembryonic antigen-related cell adhesion molecules (CEACAMs) and heparan sulfate proteoglycans (HSPGs) on epithelial cells, neutrophils, and lymphocytes. Opa proteins engage multiple CEACAM receptors (CEACAM1, 3, 5, and 6), facilitating bacterial adhesion and invasion and modulating both innate and adaptive immune responses.^329–332^ Opa binding to CEACAM1 on epithelial cells promotes colonization and prevents epithelial shedding, while interaction with CEACAM3 on neutrophils enhances phagocytosis and bacterial killing.^333^

*H. pylori* express an exceptionally large family of OMPs, with approximately 30 *hop* (Helicobacter OMP) genes encoding adhesins that bind to diverse host glycan and protein receptors.^334^ Hop proteins are 8-stranded OMPs with a large extracellular domain (varying between 15-108 kDa) inserted in the first extracellular loop.^335^ Three examples of Hop proteins are the blood group antigen-binding adhesin (BabA), sialic acid-binding adhesin (SabA), and Helicobacter OMP Q (HopQ). BabA binds Lewis b and related ABO blood group antigens on gastric epithelial cells.^336,337^ SabA binds to sialyl-dimeric-Lewis x glycosphingolipid, which facilitates persistent colonization during chronic infection.^338^ HopQ binds to CEACAMs on gastric epithelial cells and promotes adherence.^339^

Trimeric autotransporter adhesins (TAAs; also known as type Vc secretion systems) represent another functionally diverse OMP family, with the prototype YadA from *Yersinia enterocolitica*. YadA forms a homotrimeric and facilitates bacterial adhesion and confers autoagglutination, serum resistance, and protection from phagocytosis.^340–342^ The closely related *N. meningitidis* adhesin NadA binds β1 integrins to mediate epithelial cell adhesion and invasion.^343,344^ NadA is one of the four protein components of the Bexsero® vaccine.^345^ TAAs vary significantly in the size of their extracellular domains. For example, the TAA AtaA, from *Acinetobacter*, is 3630 amino acid and forms a fibrous structure that is more than 1 MDa and 260 nm long.^346^

Intimin and invasin are both inverse autotransporters (Type Ve secretion system), comprising an N-terminal β-barrel translocation unit embedded in the OM and a C-terminal extracellular passenger domain that mediates host adhesion (see Section 10.1.4).^347^ Intimin, expressed by enteropathogenic and enterohemorrhagic *E. coli*, presents its passenger domain to engage the translocated intimin receptor (Tir), which the bacterium delivers into the host cell membrane via the T3SS,^348^ as well as host nucleolin^349^ and β1 integrins^350^ in Tir-independent interactions. Invasin, expressed by *Yersinia* species, similarly projects its C-terminal passenger domain to bind multiple β1 integrin family members (α3β1, α4β1, α5β1, α6β1, αvβ1) with picomolar affinity, triggering bacterial internalization through a zipper-like mechanism^351,352^ and proinflammatory cytokine production.^353,354^

#### 6.3.2 LPS in Pathogenesis

LPS is recognized by the host innate immune system primarily by the TLR4-myeloid differentiation factor (MD-2) signaling complex.^244,355^ LPS has substantial antigenic diversity through modifications such as altered acylation and phosphorylation patterns, unusual glycosidic linkages, and the incorporation of rare monosaccharides.^225,244^ This structural heterogeneity directly affects host immune recognition and susceptibility to immune-mediated clearance. Chemical variations within the O-antigen and core oligosaccharide regions can alter the bacterial sensitivity to complement-mediated killing and antibody recognition. However, chemical variations in lipid A enable pathogens to evade innate immune detection.^225^ In particular, modifications to lipid A, such as reduced acylation or altered phosphate group decoration, can attenuate TLR4 activation and thereby diminish host inflammatory responses and promote bacterial persistence.^225,356^

Beyond immunological functions, LPS also serves as a critical structural component of the OM by contributing to the membrane integrity and permeability control. The densely packed and highly negatively charged O layer shields the bacterium from environmental stressors, including detergents, bile salts, and antimicrobial compounds.^357^ Hydrophobic antibiotics such as macrolides, rifamycin, novobiocin, and fusidic acid preferentially partition into the lipid A domain of LPS but are severely impeded by the tight packing and low membrane fluidity imposed by its saturated acyl chains.^358^ The acylation state of lipid A and sugar composition of the O-antigen influence the overall permeability of the OM and antibiotic susceptibility.^358^

The essential role of LPS in bacterial viability and its strong immunogenic properties have made it a target for vaccine development. Certain modified forms of LPS have been used in the design of live-attenuated and glycoconjugate vaccines due to their ability to stimulate protective antibody responses.^359^ However, these development efforts have been complicated by the extensive structural variability of LPS among bacterial serotypes, which has hindered the achievement of broad spectrum protection.^244,359^

#### 6.3.3 OM Lipoprotein in Pathogenesis

OM lipoproteins in diderm bacteria play direct roles in host–pathogen interactions. Surface lipoproteins of pathogenic bacteria have a wide variety of functions, ranging from metal scavenging and acquisition to hijacking of host cellular machinery. A few examples are described to illustrate the different roles that lipoproteins play in bacterial pathogenesis. TbpB is a surface lipoprotein that is involved in iron acquisition in many pathogenic diderm bacteria such as *N. meningitidis, Moraxella bovis, and Actinobacillus suis*.^360^ TbpB binds to host iron transport proteins, such as transferrin.^360^ OspE in *B. burgdorferi* binds to an inhibitor of the alternative complement pathway of the immune system, Factor H (FH).^361^ OspE binds and recruits FH to the bacterial cell surface, thereby disrupting the complement cascade and evading complement-mediated cell lysis.^361^

#### 6.3.4 OM Vesicles in Pathogenesis

OMVs are spherical nanostructures that originate from blebbing of the OM and play a significant role in bacterial pathogenicity.^362^ These vesicles contain proteins, lipids, and LPS that can modulate host responses far away from the bacteria of origin.^363^ OMVs facilitate the dissemination of virulence factors, enabling bacteria to deliver toxins, proteases, and phospholipases to host cells without requiring direct bacterial–host interaction.^362,363^ For example, *P. aeruginosa* OMVs contain enzymes such as protease, phospholipase C, alkaline phosphatase, and proelastase, all of which contribute to host tissue damage and the establishment of infection.^364^ OMVs also function as “immune decoys” that absorb and neutralize antimicrobial peptides, thereby reducing the efficacy of antimicrobial treatments against pathogenic bacterial populations.^363,365^ Some pathogens use OMVs to modulate host immune responses by selectively up- or downregulating inflammation depending on OMV cargo and the infection stage.^363^

### 6.4 OM Therapeutic Targets

#### 6.4.1 OM Antibiotic Targets

The OM of diderm bacteria has many therapeutic targets across the three components: LPS, OMPs, and surface-exposed lipoproteins. Essential components and enzymes critical to their synthesis are viable antibiotic targets. LPS, surface OMPs, and OMV-packaged antigens are directly accessible to host immune surveillance, making them central to successful vaccine adjuvant platforms.^128^

LPS biosynthesis involves multiple enzymatic steps, none of which have mammalian counterparts. Enzymes that synthesize lipid A are essential for the survival of most diderm bacteria. LpxC is the most intensely pursued target. Multiple inhibitors have demonstrated potent antibacterial activity in preclinical models.^366^ LpxH is also a promising target. Inhibitors with *in vivo* activity against wild-type *Enterobacterales* are reported to suggest that LpxH is a viable next-generation target for multidrug-resistant diderm infections.^367^ The Lpt transenvelope complex (LptABCDEFG) is structurally well-characterized, and competitive inhibitors of LptA-LptC and LptD-LptE interactions have been identified. Although preliminary, these inhibitors provide proof of concept for blocking LPS insertion as a bactericidal strategy.^368^ A clinical candidate, zosurabalpin (RG6006), derived from tethered macrocyclic peptide antibiotics with potent antibacterial activity against carbapenem-resistant *A. baumannii* that (in phase 3 trials, NCT05614895) inhibits the LptB_2_FGC complex and blocks the transport of bacterial lipopolysaccharide from the inner membrane to the outer membrane.^369,370^ The BAM complex represents another OM target with several bactericidal inhibitors identified.^371–374^ However, a potential resistance to BamA-targeting inhibitors can arise through the upregulation of BamK, a BamA paralog in some bacteria.^372,374,375^

#### 6.4.2 OM Vaccine Targets

The OM has many surface components already used in vaccines. Lipid A derivatives as adjuvants, OM proteins as antigens, and OM vesicles as multicomponent antigen-delivery platforms used effectively to generate vaccines. To use lipid A as an adjuvant, isolated lipid A is chemically detoxified through a two-step process of acid and base hydrolysis, which remove specific phosphate and acyl groups to produce 3-O-desacyl-4’-monophosphoryl lipid A (3D-MPLA).^376^ 3D-MPLA powerfully activates TLR4 and avoids the inflammatory toxicity seen with native LPS. 3D-MPLA is the active immunostimulatory component of the AS01 adjuvant system, which is incorporated in Shingrix (recombinant zoster vaccine), RTS, S/AS01E (malaria vaccine), and an approved RSV vaccine. AS01 triggers a rapid innate immune response that in turn shapes durable, antigen-specific T-cell and antibody responses, and this effect holds regardless of a recipient’s pre-existing immune status.^377^ The four components of the meningococcal serogroup B vaccine 4CMenB (Bexsero, GSK) consist entirely of OMPs, lipoproteins, and OMVs: the lipoproteins factor H binding protein (fHbp) and Neisseria heparin-binding antigen (NHBA), the OMP NadA (a TAA OMP), and an OMV incorporating the OMP PorA.^378^ The 4CMenB vaccine effectiveness against invasive MenB disease is 71–95%, with cross-protection observed against non-B serogroups and moderate cross-protection against *N. gonorrhoeae* in adolescents.^378^ The extracellular domains of TAAs are antigens in several other vaccines. For example, pertactin and filamentous hemagglutinin (from *Bordetella pertussis*) are TAA antigens for acellular pertussis (aP) vaccines including DTaP.^379^ More broadly, OMVs are an increasingly utilized vaccine platform: naturally produced or engineered OMVs can carry LPS, PG fragments, lipoproteins, and OMPs and provide intrinsic adjuvant activity.^380^ The ability to genetically engineer OMV cargo and to detoxify or modify the LPS has expanded their application across diverse bacterial pathogens in preclinical and early clinical development.^380^

## 7 Overview of the Components and Organization of Mycobacterial Cell Envelope

Mycobacteria, belonging to the *Terrabacteria* clade within the Actinobacteria phylum, possess one of the most distinctive and pathogenically relevant cell envelopes among bacteria.^218,220^ The mycobacterial OM (MOM) (Figure 8) is different from that of diderm bacteria.^218,220^ Mycolic acids (long-chain fatty acids consisting of approximately 70-90 carbons) coat the inner leaflet, which constitutes approximately 30% of the cell envelope dry weight.^218,220^ The mycolic acid leaflet is covalently linked to arabinogalactan, which is in turn covalently attached to PG, forming the mycolyl-arabinogalactan-PG (mAGP) complex (Figure 8).^218,381^ This covalent integration, which is unique among bacteria, creates an impenetrable barrier. The mAGP complex confers antibiotic resistance and is crucial to viability; mutations reduce mycolic acid content, alter colony morphology, decrease persistence in host cells, or create unstable OMs with altered drug permeability.^381^ Complete loss of mycolic acids is lethal to *Mycobacterium tuberculosis* but not lethal to the closely related *Corynebacterium* species, the specialized adaptations mycobacteria have undergone.^381^ The outer leaflet contains loosely-packed phthiocerol dimycocerosate (PDIM), trehalose dimycolate (TDM, or cord factor), trehalose monomycolate (TMM), and other complex glycolipids (Figure 8).^381^

**Figure 8:**
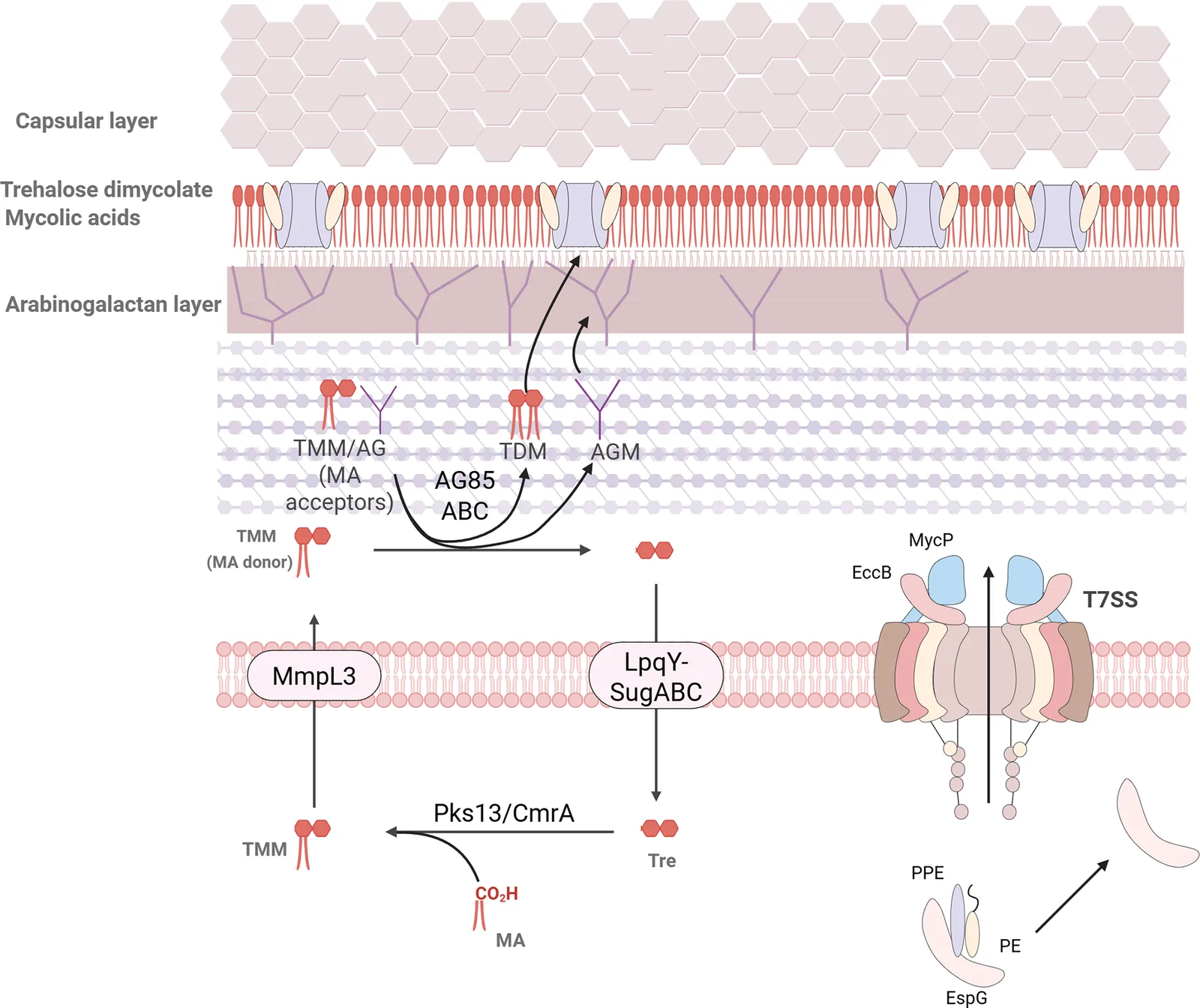
**Mycomembrane biogenesis.** The mycobacterial cell envelope is organized in concentric layers: the IM, the arabinogalactan layer, and an outer mycomembrane composed of trehalose dimycolate (TDM) and mycolic acids, topped by an outermost protein layer. Mycolic acid biosynthesis is initiated in the cytoplasm by Pks13/CmrA, which condenses mycolic acid (MA) precursors to produce trehalose monomycolate (TMM). TMM is transported across the IM by the MmpL3 transporter and serves as the mycolic acid donor for transfer reactions in the arabinogalactan layer, generating TDM and arabinogalactan mycolates (AGM). The Ag85ABC complex facilitates additional mycolic acid transfer steps within the arabinogalactan layer. The LpqY-SugABC system imports trehalose (Tre) from the mycomembrane back to the cytoplasm to sustain TMM synthesis. PE (proline-glutamate) and PPE (proline–proline–glutamate) surface proteins are secreted via the Type VII secretion system (T7SS), whose machinery includes the membrane-spanning EccB subunit and the surface-associated protease MycP. The cytoplasmic chaperone EspG binds a conserved hydrophobic motif in the N-terminal domain of the PPE protein and directs the PE/PPE heterodimeric complex to the cognate ESX translocon for translocation across the IM as a heterodimer. Created in https://BioRender.com.

Porins in the mycomembrane facilitate the diffusion of hydrophilic nutrients across the mycobacterial OM, which is otherwise highly impermeable because of its mycolic acid content. MspA, the major porin of *Mycobacterium smegmatis*, is the first structurally characterized mycobacterial OMP and is homooctameric with a single central channel approximately 9.6 nm in length that facilitates the passive diffusion of small, hydrophilic solutes.^382,383^ OmpATb specifically facilitates permeability to small water-soluble nutrients such as serine and plays a role in enabling *M. tuberculosis* to respond to acidic pH conditions.^384^

Several other types of proteins associate with the mycobacterium that are not β-barrel OMPs. In *Corynebacterium glutamicum*, PorA and PorH form hetero-oligomeric channels essential for nutrient uptake.^385,386^ PorA requires mycoloylation, a post-translational modification unique to *Corynebacteriales*, for proper channel function, and both proteins are co-transcribed and must associate to form functional pores in the mycomembrane.^387^ Mycobacterial species have a set of proteins (Proline-glutamate (PE) and proline–proline glutamate (PPE) proteins) that non-covalently associate with the outer leaflet of the mycomembrane and form non-specific channels that allow small molecule nutrients to permeate the mycomembrane.

### 7.1 Mycomembrane Biogenesis

#### 7.1.1 Mycolic Acid Biosynthesis, Transport, and Mycomembrane Assembly

Mycolic acid biosynthesis proceeds through a sequential two-stage fatty acid synthesis pathway unique to mycobacteria. The multifunctional eukaryotic-type FAS-I enzyme first synthesizes medium-chain fatty acids (C16–C26) from acetyl-CoA.^388^ These products are then elongated by the discrete, ACP-dependent FAS-II system, composed of monofunctional enzymes including the condensing enzymes KasA and KasB, the dehydratases HadAB and HadBC, the ketoreductase MabA, and the enoyl-ACP reductase InhA. The final elongated products are meromycolic acids of C40–C60 length.^389,390^ Unlike FAS-II systems in other bacteria, the mycobacterial FAS-II cannot initiate de novo synthesis and is dedicated solely to elongation of FAS-I products.^391^ The meromycolic acids are subsequently modified by S-adenosylmethionine-dependent methyltransferases that introduce cyclopropane rings, methyl branches, and oxygenated functionalities defining the three major mycolic acid classes: alpha-, keto-, and methoxymycolates.^392,393^ Final condensation of the meromycolate chain with a C24–C26 fatty acid derived from FAS-I is catalyzed by the polyketide synthase Pks13, which performs a Claisen-type condensation and thioesterase-mediated release to yield the mature mycolic acid.^389,394^

Following synthesis at the cytoplasmic membrane, mature mycolic acids must be transported across the IM and arabinogalactan layer to assemble the mycomembrane’s outer leaflet. This flipping step is mediated primarily by MmpL3, an IM transporter, which exports mycolic acids in the form of TMM.^393^ Cryo-EM and X-ray structures of MmpL3 have revealed a proton motive force-driven transport mechanism, with a flexible linker between the two transmembrane subdomains implicated in the translocation process.^395^ Once TMM reaches the periplasm and arabinogalactan layer, the mycolyltransferases Ag85A, Ag85B, and Ag85C (serine esterases of the carboxylesterase family) covalently attach mycolic acids from TMM to arabinogalactan termini to form arabinogalactan mycolates (AGMs) or to a second TMM molecule to generate trehalose dimycolate (TDM).^396,397^ The spatial coordination of this process is regulated in part by the periplasmic protein PgfA, which interacts with MmpL3 and influences the polar localization of TMM transport, thereby linking mycomembrane assembly to the asymmetric growth program of mycobacteria.^398^

#### 7.1.2 Mycomembrane-Associated Protein Biogenesis

Proteins associated with or inserted into the mycomembrane face unique biogenesis requirements as they must navigate through the IM, periplasm, and mAGP complex to reach the mycomembrane. With respect to OMP biogenesis, it remains unknown how mycomembrane OMPs are inserted into the mycomembrane; a BAM homologue has not been identified.

Proline–glutamate (PE) and proline–proline glutamate (PPE) proteins are transported across the mycobacterial IM via Type VII (ESX) secretion systems and associate to the outer surface of the mycomembrane (see Section 10.1.6). PE/PPE proteins form heterodimeric complexes (Figure 8) that are recognized by system-specific cytoplasmic chaperones called EspG.^399,400^ The EspG chaperones bind to a conserved hydrophobic hh motif in the PPE protein’s N-terminal domain, preventing aggregation of the PE/PPE complex and directing it to the cognate ESX translocon (ESX-1, ESX-3, or ESX-5) for translocation across the IM.^399,401^ The EspG-binding domain on PPE proteins serves as the primary determinant of the system specificity. Swapping the EspG1-binding domain for the EspG5-binding domain reroutes substrates from ESX-1 to ESX-5.^402^ Following translocation, PE/PPE surface proteins localize to and become anchored to the mycomembrane OM, where they remain surface-associated.^403–405^ While the precise molecular mechanism of membrane anchoring remains unknown, once localized to the mycomembrane, PE/PPE proteins form small-molecule channels that facilitate the uptake of essential small-molecule substrates across the hydrophobic mycobacterial OM barrier.^404,406,407^

### 7.2 Mycomembrane in Pathogenesis

Surface-exposed PE/PPE proteins represent a large and functionally diverse protein family that plays critical roles in nutrient acquisition and host–pathogen interactions in mycobacteria. PPE51 (Rv3136) functions as a gateway for trehalose uptake across the mycomembrane. Deletion of the *ppe51* gene prevents bacterial growth on trehalose, maltose, and lactose as sole carbon sources, but maintains bacterial growth on glucose and glycerol.^408,409^ In *M. smegmatis*, the PE9/PE10 heterodimer is a unique example of PE/PE protein interaction, which deviates from the typical PE/PPE interaction more commonly found.^410^ This surface-localized complex interacts with TLR4 on macrophage and triggers the TRIF pathway and enhances interferon-β secretion.^410^ PPE36 and PPE62 are involved in heme-iron acquisition.^406^ PPE36 modulates host inflammatory responses by promoting ubiquitination and degradation of a signal transducer for the immune system.^411^ PPE38 is required for the secretion of all PE and PPE proteins via the ESX-5 system (Figure 8 and 11 and Section 10.1.6), with loss-of-function mutations in *ppe38* preventing the secretion of these protein families.^404^

Mycobacterial glycolipids are critical virulence factors with dedicated immune evasion functions.^381,412^ Trehalose dimycolate, the most abundant glycolipid in *M. tuberculosis*, directly interacts with C-type lectin receptors on macrophages. This interaction impedes phagosome acidification and promotes granuloma formation, both of which facilitate bacterial persistence.^413^ Phthiocerol dimycocerosate facilitates escape from phagolysosomes and contributes to virulence; its absence in bacterial strains reduces the pathogenicity. The hydrophobic mycolic acid layer facilitates the release of immunomodulatory lipids and also a high degree of impermeability.^414^

### 7.3 Mycomembrane Therapeutic Targets

#### 7.3.1 Mycomembrane Antibiotic Targets

The mycobacterial cell envelope is a clinically relevant bacterial surface for the development of antibiotics and vaccines. The global burden of tuberculosis motivates therapeutic development, and the unique structural properties of the mycomembrane provide therapeutic targets specific to *M. tuberculosis*. The biosynthetic enzymes and transporters that assemble this barrier are critical for *M. tuberculosis* viability and provide multiple therapeutic targets.^415,416^

Ethambutol targets the arabinosyltransferases (EmbB and EmbC), which catalyze arabinosylation of arabinogalactan, yet resistance is emerging.^417^ The cryo-EM structure of *M. smegmatis* EmbB confirmed that resistance mutations cluster around the putative active site, consistent with direct drug-protein interaction.^418^ The antigen 85 complex (Ag85A, Ag85B, Ag85C), is another validated cell wall target. Cyclipostins and cyclophostin analogues bind covalently to the catalytic serine of Ag85C and inhibit both mycolyltransferase and triacylglycerol synthase activities, and reduce trehalose dimycolate synthesis and arabinogalactan mycolylation in culture.^419^ The FAD-dependent oxidase (DprE1) that contributes to the synthesis of arabinogalactan was identified as the target of benzothiazinones (BTZs) through a genetic and biochemical screen.^420^ BTZ043 kills *M. tuberculosis in vitro*, *ex vivo*, and in murine tuberculosis (TB) models and was the first compound to establish DprE1 as a druggable target.^421^ Subsequent optimization produced PBTZ169 (macozinone) and a broad range of non-covalent inhibitor scaffolds.^422^ The recently approved BPaLM regimen is a cocktail of bedaquiline, pretomanid, linezolid, and moxifloxacin. Pretomanid targets cell wall synthesis indirectly by inhibiting cytochrome bd oxidase of the electron-transport chain.^423^ Overall, specific inhibitors of mycobacterial cell envelope synthesis are underdeveloped despite the promise of these inhibitors to increase the accessibility of antibiotics across the mycomembrane.^424^

#### 7.3.2 Mycomembrane Vaccine Targets

TB vaccine development is an active area of bacterial vaccine research globally, with 16 candidates currently in clinical evaluation and four in active Phase 3 efficacy trials. The currently used Bacillus Calmette-Guérin (BCG) vaccine provides inconsistent and incomplete protection against adult pulmonary disease.^425^ The most advanced vaccine candidate is M72/AS01E, a recombinant fusion protein derived from two *M. tuberculosis* antigens (Mtb32A and Mtb39A, both involved in cell wall metabolism and persistence) formulated with the AS01E adjuvant system containing 3D-MPLA (mentioned in Section 7.3.2) and QS-21 (a triterpene saponin that activates the immune system’s antigen-presenting cells). In GSK’s Phase 2b trial, M72/AS01E demonstrated approximately 50% protection against progression to active pulmonary TB over three years without safety concerns, establishing proof of concept for a protein subunit TB vaccine.^426^ A Phase 3 efficacy trial initiated in March 2024 and results are anticipated within approximately five years.^425^ Surface-exposed PE/PPE proteins are a functionally diverse antigen family with established immunogenicity in natural infection. PPE18 is both a virulence factor and a potential vaccine antigen.^427^ Proteins secreted by the ESX-1 type VII secretion system (specifically ESAT-6 and CFP-10) are among the most immunodominant *M. tuberculosis* T cell antigens and are included in multiple subunit vaccine formulations. The inclusion of these proteins in next-generation vaccines is expected to improve on BCG’s limited efficacy against adult pulmonary disease.^415,425^

## 8 Overview of the Components and Organization of Bacterial Capsules

Bacterial capsules are surface-associated polymers that envelop the cell exterior from the cell wall. By forming a thick, hydrated barrier outside the cell wall or OM, capsules protect bacteria from desiccation, bacteriophage predation, and host immune defenses, including phagocytosis, complement-mediated killing, and antimicrobial peptides.^428^ The specific contribution of a given capsule to virulence depends on its chemical composition, charge, and structural relationship to host molecules, and can include resistance to phagocytosis, evasion of complement-mediated killing, and molecular mimicry of host tissue, discussed in Section 8.2. Because the capsule is often the most exposed and immunogenic bacterial surface structure, it is subject to strong diversifying selection by host adaptive immunity: serotype-specific antibodies favor bacteria that switch to antigenically distinct capsule types, driving the extensive serotype diversity seen within single pathogens.^429^ In *E. coli*, this diversity is further amplified by recombination and plasmid-mediated horizontal transfer of capsule gene clusters between species.^428^ Capsules are anchored to either the PG layer in monoderm bacteria or the OM in diderm bacteria, and they are classified primarily by their chemical composition.^430,431^ The most common capsules are polysaccharide-based, but polypeptide- and hybrid-based capsules also exist. Within the polysaccharide category, capsules are further divided into heteropolysaccharide types, which are composed of two or more distinct monosaccharides in their repeat units, and homopolysaccharide types, which are built from a single sugar or disaccharide. The diversity of polysaccharide capsule structures arises from variation in monosaccharide identity, glycosidic linkage position and configuration (L or D), chain branching, and chemical modifications such as O-acetylation, pyruvylation, and phosphorylation.^432^

### 8.1 Polysaccharide Capsules

Heteropolysaccharide capsules are the most prevalent capsule type and are distributed broadly across both monoderm and diderm pathogens. Their repeat units typically contain two to six monosaccharides, including common sugars such as glucose (Glc), galactose (Gal), mannose (Man), rhamnose (Rha), fucose (Fuc), N-acetylglucosamine (GlcNAc), N-acetylgalactosamine (GalNAc), and N-acetylmannosamine (ManNAc). Uronic acids, including glucuronic acid (GlcA) and galacturonic acid (GalA), are frequently incorporated and confer negative charge to the capsule. Despite common components, there is wide diversity in capsule composition and within serotypes of the same bacteria. *S. pneumoniae* produces at least 93 antigenically distinct capsule serotypes, most of which are built on trisaccharide repeating units, although hexasaccharide (serotype 12F) and tetrasaccharide (serotypes 4 and 6A) repeat units also occur.^433,434^ *K. pneumoniae* produces at least 77 serotypes with main-chain monosaccharides that include mannose, glucose, galactose, fucose, and rhamnose.^435^ *E. coli* produces approximately 80 antigenically distinct capsule types.^436^ *N. meningitidis* is divided into serogroups A, B, C, W, X, Y, and Z based on capsule polysaccharide structure. Serogroups B and C carry polysialic acid capsules and are described below. Serogroup A capsule is composed of α-1,6-linked N-acetylmannosamine-1-phosphate. Serogroup W carries a capsule of alternating sialic acid and galactose residues, and serogroup Y carries alternating sialic acid and glucose residues.^437^ *Haemophilus influenzae* produces six capsule types designated a through f. Serotype b (Hib) carries a polyribose ribitol phosphate capsule in which ribose and ribitol units are joined by phosphodiester bonds.^438,439^ Serotype a capsule consists of a polyribose ribitol phosphate polymer similar to but antigenically distinct from the Hib capsule.^440^ *S. aureus* produces at least 11 capsule types and *Streptococcus agalactiae* (group B Streptococcus, GBS) produces nine serotypes (Ia, Ib, and II through VIII), which contain terminal sialic acid residues on their side chains, and the serotype differences arise from variation in the underlying backbone sugars.^441^ These are just a few examples; many more bacteria and serotypes have been identified.

Homopolysaccharide capsules are composed of a single monosaccharide or disaccharide unit polymerized into long linear chains. A defining feature of this group is structural identity or close resemblance to mammalian glycosaminoglycans or cell-surface glycans, which confer immunological advantages discussed further in the pathogenicity section. Hyaluronic acid (HA) capsules are composed of alternating β-1,3-linked GlcNAc and β-1,4-linked GlcA residues, an exact chemical replica of the vertebrate extracellular matrix component hyaluronic acid.^442^ HA is a non-sulfated glycosaminoglycan compared to sulfated glycosaminoglycans such as heparin, heparan sulfate, chondroitin sulfate, dermatan sulfate and keratan sulfate. HA capsules are produced by *S. pyogenes* (Group A *Streptococcus*),^443^ *Streptococcus equi*,^444^ *Pasteurella multocida* serogroup A,^445^ and many others. Polysialic acid (PSA) capsules are composed of polymerized N-acetylneuraminic acid (Neu5Ac, sialic acid) residues. *E. coli* K1 ^446^ and *N. meningitidis* serogroup B have α-2,8-linked PSA.^447^ *N. meningitidis* serogroup C carries α-2,9-linked PSA.^447^ *Mannheimia haemolytica* serotype A2, a bovine respiratory pathogen, also produces a PSA capsule.^448^ Heparosan capsules are composed of alternating α-1,4-linked GlcNAc and β-1,4-linked GlcA residues, structurally resembling the mammalian glycosaminoglycan heparin before its sulfation. *E. coli* K5 is the canonical heparosan-producing strain and serves as the industrial source for biosynthetic heparin production.^449^ Chondroitin-like capsules are composed of alternating GlcNAc and GlcA residues in a β-1,3/β-1,4-linked pattern, present in *E. coli* K4,^450^ and resemble the mammalian glycosaminoglycan chondroitin. Another capsule type, the Vi capsule, is found in *Salmonella enterica* serovar Typhi,^451^ *Citrobacter freundii*,^452,453^ members of *Burkholderiales*.^454^ Vi capsule is composed of α-1,4-linked N-acetylgalactosaminuronic acid residues with partial O-acetylation at the C3 position^455^ and constitutes a distinct homopolysaccharide type.

#### 8.1.1 Peptide Capsules

A small number of bacteria produce capsules composed of amino acid polymers rather than sugars. Poly-γ-d-glutamic acid (PDGA) capsules are composed of d-glutamic acid residues polymerized through their γ-carboxyl groups into long, high-molecular-weight chains.^456^ PDGA capsules are produced by *S. epidermidis, B. anthracis* ^457^ and *Bacillus licheniformis*.^458^ *B. subtilis* ^459^ and *S. epidermidis* ^460^ produces PDGA synthesized from both D- and l-glutamic acid. However, rather than use the polymer as a capsule, it secretes the polymer into the environment as a biofilm.^461^ The F1 protein capsule is found only in *Yersinia pestis* and is composed of non-covalently aggregated linear fibrils of a single protein, Caf1.^460^ The fibrils are translocated and assembled through the fimbrial chaperone:usher system^461^ such as those described in Section 9.1.1.

#### 8.1.2 Hybrid Capsules

Some bacteria produce capsules that combine polysaccharide and protein components or that do not conform to the standard definitions above. *Bacillus megaterium* produces a capsule containing both polysaccharide and polypeptide components, although the precise structural organization of this hybrid capsule remains incompletely characterized.^462^ *Francisella tularensis* produces two distinct surface structures classified as capsules. The O-antigen capsule is a polysaccharide composed of mannose, rhamnose, and deoxy sugars.^463^ The capsule-like complex (CLC) contains both protein and carbohydrate components and shares features with a loosely associated surface coat.^464^

#### 8.1.3 Capsule Modifications

Chemical modifications alter the physical properties, charge, and antigenicity of CPSs without changing their core repeat unit. O-Acetylation is one of the most widespread modifications and is often the sole determinant of serological differences between otherwise structurally similar capsules.^465^ In *S. pneumoniae*, inactivation of the WcjE O-acetyltransferase converts serotype 11A to serotype 11E, eliminating the O-acetyl epitope recognized by the lectin ficolin-2.^466^ In *S. aureus*, the O-acetylation pattern distinguishes serotype 5 from serotype 8.^467^ Pyruvylation adds a pyruvate ketal to a hydroxyl group of a sugar residue, introducing additional negative charge and antigenic specificity. The serotype 4 *S. pneumoniae* capsule contains a pyruvate modification on its tetrasaccharide repeat unit.^468^ Phosphate groups serve as structural bridges in some capsules, most notably in the *H. influenzae* type b polyribosylribitol phosphate capsule, where phosphodiester bonds link the ribose and ribitol units of the repeating chain.^469^

#### 8.1.4 Capsule Anchoring

Surface anchoring differs between monoderm and diderm bacteria, and even among diderms, it differs by capsule biosynthesis pathway (Section 8.1). Group 1 (Wzy-dependent) capsules are exported through the Wza translocon and captured non-covalently at the cell surface by the outer membrane lectin Wzi (Section 8.1.1).^293^ Group 2/3 (ABC transporter-dependent) capsules, by contrast, remain covalently attached to the phosphatidylglycerol-Kdo glycolipid on which they were synthesized, which anchors the mature polysaccharide in the OM (Section 8.1.3).^470^ In monoderm bacteria, completed polysaccharide chains are covalently attached to the PG layer by LCP family enzymes. One notable exception is *S. pneumoniae* serotype 3, whose capsule associates with the IM non-covalently through interactions with phosphatidylglycerol rather than through a covalent cell wall linkage.^471^ Polypeptide capsules have distinct anchoring strategies. The PDGA capsule of *B. anthracis* is tethered covalently to PG via the CapBCE synthetase complex.^472^

### 8.2 Bacterial Capsule Biogenesis and Assembly

#### 8.2.1 The Wzx/Wzy-Dependent Pathway

The Wzx/Wzy-dependent pathway (Figure 9A) is the most widely distributed mechanism for capsular polysaccharide synthesis in both monoderm and diderm bacteria.^431^ This pathway proceeds through the stepwise assembly of lipid-linked repeat units on the cytoplasmic face of the IM, followed by their flipping and polymerization on the periplasmic face. More than 90% of *S. pneumoniae* serotypes rely on this pathway, as do group I and group IV *E. coli* capsules and many other encapsulated pathogens.^431^

**Figure 9:**
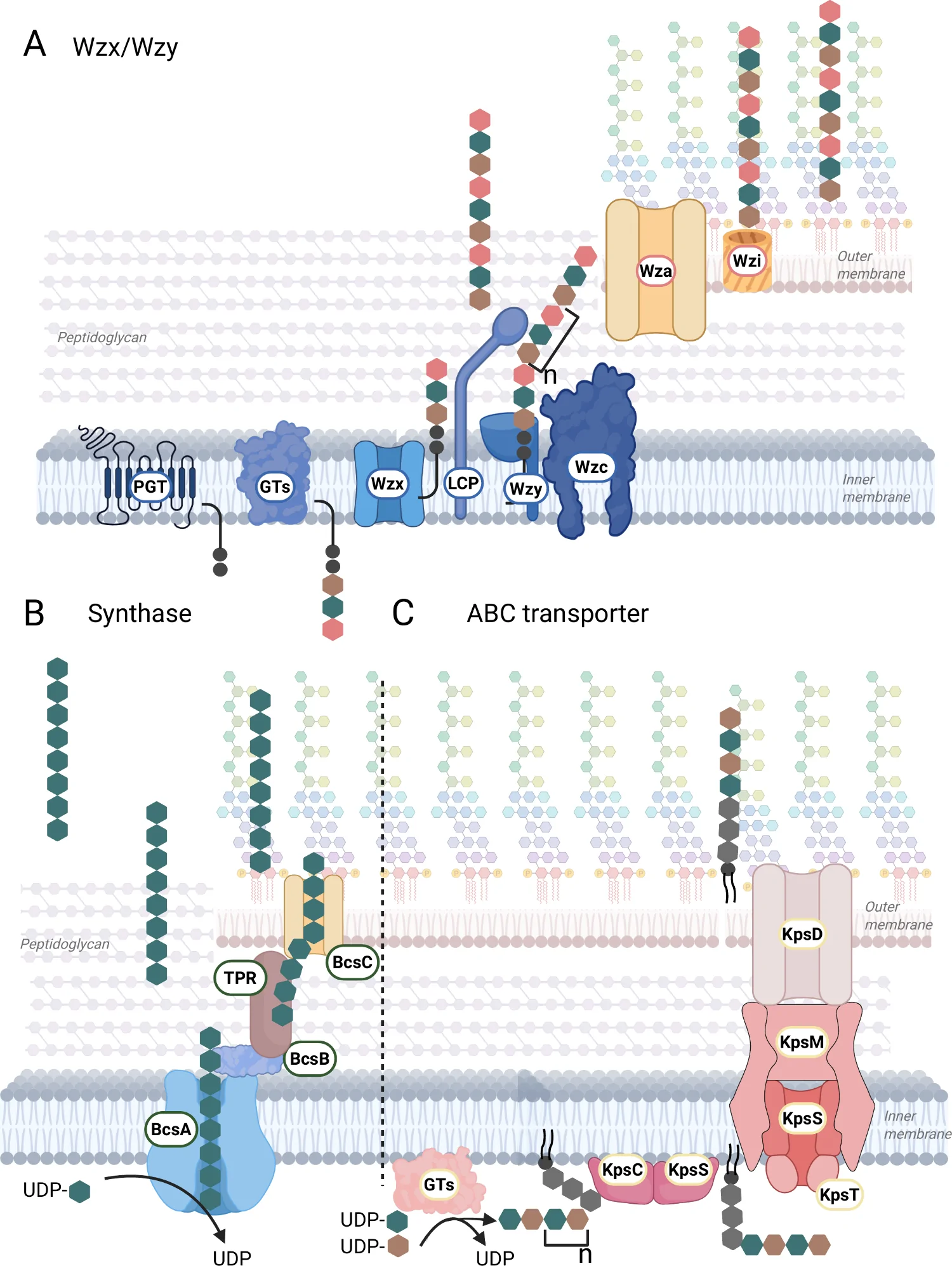
**Capsular polysaccharide (CPS) biosynthesis pathways in bacteria.** Three conserved CPS biosynthetic systems are depicted. **(A) Wzx/Wzy-dependent pathway.** CPS synthesis is initiated at the IM by a polyprenol phosphate phosphoglycosyltransferase (PGT), which transfers the first sugar of the repeat unit onto the undecaprenyl phosphate (Und-P) lipid carrier. Additional glycosyltransferases (GTs) sequentially add nucleotide sugars to complete the repeat unit on the cytoplasmic face of the IM. The lipid-linked repeat unit is then flipped across the IM by the Wzx flippase. On the periplasmic face, the Wzy polymerase couples incoming repeat units to the growing chain. The polysaccharide co-polymerase Wzc, a tyrosine autokinase, regulates chain length through cycles of autophosphorylation and dephosphorylation of its C-terminal tyrosine cluster. In bacteria with an OM, the completed CPS is exported through the OM translocon Wza, while Wzi, an OM lectin, facilitates surface retention of the capsule. In bacteria lacking an OM, LCP enzymes transfer the completed polymer directly onto MurNAc residues of the PG layer. The diverse capsular polysaccharide chains at the cell surface reflect the structural variability generated by this pathway across bacterial species. **(B) Synthase-dependent pathway.** A processive synthase complex (exemplified by the BcsAB cellulose synthase) simultaneously polymerizes and translocates the CPS chain. BcsA, the catalytic subunit, synthesizes the polymer directly from UDP-sugar precursors at the IM without a lipid-linked repeat unit intermediate; the intracellular concentration of nucleotide-sugar substrates is a key determinant of chain length. BcsB directs the nascent chain through the periplasm, and BcsC, a β-barrel translocon with a periplasmic tetratricopeptide repeat (TPR) domain, facilitates export across the OM. Modifying enzymes further elaborate the polymer following synthesis. **(C) ABC transporter-dependent pathway.** CPS synthesis is initiated by sugar-specific glycosyltransferases (GTs) that build the polymer on a conserved glycolipid terminus in the cytoplasm. The completed glycolipid-CPS is translocated across the IM by the ABC transporter complex KpsMT (KpsC and KpsS synthesize the conserved poly-Kdo linker that bridges the lipid anchor to the polysaccharide). The periplasmic polysaccharide co-polymerase KpsE (PCP-3) and the OM export protein KpsD (OPX) facilitate translocation across the periplasm and OM. The CPS is retained at the cell surface via its glycolipid anchor embedded in the OM, or through ionic interactions with LPS. Periplasmic enzymes that modify the polysaccharides exist, but are not shown in the schematics. Created in https://BioRender.com and adapted from Figure 1 in.^922^ Available under a CC-BY 4.0 license. Copyright 2025 Khadka et al.

The process begins with a phosphoglycosyltransferase (PGT) that transfers a sugar-1-phosphate from a nucleotide sugar donor onto the lipid carrier undecaprenyl phosphate (Und-P), generating undecaprenyl diphosphate (Und-PP)-linked repeat units on the cytoplasmic leaflet of the IM. Serotype-specific glycosyltransferases (GTs) sequentially add sugars to the lipid-linked primer. In *S. pneumoniae*, both CpsE (WchA) and related glycosyltransferases from the WcjG or WcjH homologous families carry out the first sugar transfer, depending on whether glucose is part of the repeat unit.^473^ Once the initial monomer unit is assembled, the integral membrane flippase Wzx translocates it across the IM to the periplasmic face.^474^ The polymerase Wzy catalyzes nonprocessive polymerization by transferring the growing polysaccharide chain from the existing Und-PP-linked polymer onto the new repeat unit (nearly identical to the polymerase for LPS biosynthesis).^475^ This block polymerization mechanism regenerates Und-PP from the chain donor, allowing that lipid carrier to re-enter the cytoplasmic assembly cycle. Chain length is regulated by the polysaccharide co-polymerase Wzc, an IM protein with a cytoplasmic tyrosine autokinase (BY-kinase) domain.^476^ Wzc cycles between a dephosphorylated octameric state that spans the IM and a phosphorylated monomeric state. This phosphorylation cycle, which is counteracted by the phosphotyrosine phosphatase Wzb, is essential for productive capsule assembly and export.^476^ In *S. pneumoniae*, the CpsB, CpsC, and CpsD proteins form an analogous tyrosine phosphoregulatory system.^477,478^

In monoderm bacteria, a distinct anchoring mechanism is present: the completed polysaccharide chain is transferred from its lipid carrier onto a PG assembly intermediate, which is then covalently attached to the cell wall by LCP family enzymes, as described in Sections 4.1 and 5.1.1. In diderm bacteria, once polymerized, the capsular polysaccharide is translocated across the OM through the OM polysaccharide export (OPX) family channel Wza, which forms an octameric ring spanning the OM and periplasm.^293^ It is interesting to note that Wza has an α-helical barrel that spans the outer membrane in contrast to the β-barrel structures of most OMPs (Section 6.2).^293,479^ The auxiliary lectin-binding protein Wzi then assists in organizing the exported polymer into a non-covalently surface-associated capsule structure.^293^ Some bacteria also use these same OPX proteins to secrete polysaccharides for biofilm formation.^480,481^

#### 8.2.2 The Synthase-Dependent Pathway

The synthase-dependent pathway (Figure 9B), present in monoderms and diderm bacteria, differs from the ABC transporter-dependent and Wzx/Wzy-dependent pathways in that a single multifunctional enzyme or a small enzyme complex is responsible for initiating, polymerizing, and translocating the polysaccharide simultaneously without a lipid-linked intermediate.^482^ This mechanism is most common for homopolysaccharide capsules and produces simpler, often linear polymer structures. The prototypic example of the synthase mechanism in capsule biology is cellulose biosynthesis (Figure 9B), where the IM catalytic subunit BcsA couples processive UDP-glucose-dependent polymerization to co-translational export through a channel across the IM.^483^ The crystal structure of the BcsA-BcsB complex from *Rhodobacter sphaeroides* containing a translocating polysaccharide revealed how synthesis and membrane translocation are mechanistically coupled, with the nascent chain extended by one glucose residue at a time.^483^

The HA capsule synthases of *S. pyogenes* (HasA) and *P. multocida* serogroup A (PmHAS) serve as the canonical examples of pathogenic capsule assembly.^484,485^ These enzymes add alternating GlcNAc and GlcA residues to the growing HA chain at the cytoplasmic face of the membrane while extruding the completed polymer to the cell exterior, producing a product chemically identical to vertebrate hyaluronic acid.^486^ Similarly, the PSA capsule of *E. coli* K1 is synthesized by the polysialyltransferase NeuS, which elongates the poly-α-2,8-Neu5Ac chain processively from the cytoplasm.^487^

In monoderms, once they are synthesized and released into the periplasm, the polysaccharides can either attach to the PG or be released into the extracellular environment. In didermic bacteria, additional proteins are needed to transport polysaccharides to the bacterial surface. BcsB directs the chain to the periplasmic tetratricopeptide repeat (TPR) protein that guides the polysaccharide to an OM transporter (BcsC). Once across the OM, the polysaccharide is released into the extracellular environment or attached to lipid A.^488^

#### 8.2.3 The ABC Transporter-Dependent Pathway

ABC transporter-dependent polysaccharide synthesis (Figure 9C) is exclusive to diderm bacteria (e.g., Group II and Group III *E. coli* capsules) and differs fundamentally from the Wzx/Wzy pathway.^477^ In this pathway, the entire polysaccharide chain is assembled by processive polymerization in the cytoplasm completely before transfer to a lipid. The growing chain is anchored to a conserved glycolipid acceptor containing a poly-3-deoxy-D-manno-oct-2-ulosonic acid (Kdo) linker joined to a phosphatidylglycerol terminus. The Kdo linker is assembled by the sequential action of two CMP-Kdo-dependent glycosyltransferases, KpsS and KpsC.^470^ A variation of this pathway is found in the Vi-antigen capsule of *S. enterica* serovar Typhi and certain *Burkholderiales*, which lack the capacity to synthesize the β-Kdo oligosaccharide linker and instead use a distinctive diacyl-HexNAc lipid terminus assembled by the Vi-specific acyltransferase VexE.^451^

Once polymerization is complete, the KpsMT ABC transporter exports the lipid-linked polysaccharide chain across the IM into the periplasm.^489^ The IMP KpsM and OMP KpsD then cooperate to translocate the chain across the periplasm and OM, respectively, to the cell surface. KpsD localizes to the cell pole and forms part of a multiprotein biosynthetic and export complex.^490^ Despite mechanistic differences from the Wzx/Wzy route, both pathways employ OMPs belonging to the OPX family for the final translocation step.^477^ Once at the cell surface, the polysaccharide can interact with Wzi (another OMP), non-covalently with LPS, or be released to the extracellular environment.

#### 8.2.4 Protein Capsule Synthesis

The biosynthetic pathway for the PDGA capsule of *B. anthracis* and related *Bacillus* species was first elucidated in *B. licheniformis*.^491^ Synthesis is carried out by a membrane-associated polyglutamyl synthetase complex, CapB, CapC, and CapE, in *B. anthracis*.^456,492^ The reaction proceeds through the ATP-dependent activation of l-glutamic acid to form a γ-l-glutamyl adenylate intermediate, which is then racemized to the D-form and transferred to a protein-bound thioester intermediate.^456,492^ The activated d-glutamyl unit is then transferred to the terminal amino group of a membrane-bound poly-γ-d-glutamyl acceptor chain, extending it by one residue.^456,492^ This cycle continues to generate high-molecular-weight PDGA at the membrane surface. The completed polymer is then covalently anchored to the PG by CapD, a γ-glutamyl transpeptidase.^472^ Export of the full-length chain to the cell exterior and the precise mechanism of release from the membrane remain incompletely understood.^456,492^

#### 8.2.5 Regulation of Capsule Synthesis

Capsule production is tightly regulated in response to environmental cues encountered during infection, including osmotic pressure, oxygen tension, temperature, pH, CO_2_ concentration, and iron availability.^430^ For example, entry into the bloodstream, with its 0.15 M NaCl osmolarity, triggers prioritized CPS synthesis in *S. enterica* serovar Typhi via the TviA auxiliary protein, which integrates osmolarity sensing into the RcsB regulon to coordinately upregulate Vi antigen expression while repressing flagella and the type III secretion system.^493^ In *S. aureus*, surface growth conditions strongly promote capsule production relative to liquid culture growth, a distinction relevant to the *in vivo* environment.^494^ In addition, CO_2_ downregulates synthesis of *S. aureus* type 5 and type 8 capsules.^495^ Temperature is the key regulatory signal for the *Y. pestis* F1 capsule, with expression occurring at 37°C in the mammalian host but not at the ambient temperature of the arthropod vector.^496^ Capsule biosynthesis in *B. anthracis* is controlled by the virulence plasmid pXO2 and is transcriptionally induced by elevated CO_2_/bicarbonate concentrations and 37°C temperature, signals that mimic the mammalian host environment.^497^

### 8.3 Capsules in Pathogenesis

The defining pathogenic function of most polysaccharide capsules is the inhibition of opsonophagocytosis. CPS forms a thick hydrophilic layer that physically masks underlying bacterial surface antigens, including lipoteichoic acid, PG fragments, and OMPs, thereby preventing their recognition by pattern recognition receptors on phagocytes. CPS also blocks the deposition and surface accessibility of the complement opsonin C3b; the capsule sterically prevents C3b from engaging its receptor, CR1, on neutrophils and macrophages, thereby impeding opsonophagocytosis.^498^ Capsules containing sialic acid additionally recruit the complement regulatory protein factor H from serum, which acts as a cofactor for factor I-mediated cleavage of C3b to the nonopsonic fragment iC3b, preventing membrane attack complex formation.^499^ *S. agalactiae* serotypes Ia through VIII, which all carry terminal sialic acid residues, exploit this mechanism to survive in the bloodstream of neonates where complement is the primary defense.^500^ Capsule charge and hydrophilicity further contribute by creating electrostatic and steric barriers that repel negatively charged phagocyte membranes.^444^ *S. aureus* capsule types 5 and 8 inhibit opsonophagocytosis by masking surface-associated proteins that would otherwise mediate phagocyte recognition.^501^ The antiphagocytic effect is also subject to dynamic regulation during infection: capsule expression is upregulated in response to host-specific signals such as entry into the bloodstream, as seen in *S. pneumoniae*.^502^

Several capsule types exploit the molecular mimicry of mammalian glycans to evade adaptive immunity. The HA capsules of *S. pyogenes*, *S. equi*, and *P. multocida* serogroup A are chemically identical to those of vertebrate hyaluronic acid, rendering them immunologically inert and unable to elicit a robust antibody response on their own.^432^ The HA capsule of *S. pyogenes* protects against neutrophil-mediated killing and is required for efficient initial colonization of the nasopharynx and skin.^443^ The PSA capsules of *E. coli* K1 and *N. meningitidis* serogroup B are structurally identical to the polysialic acid modification on the mammalian neural cell adhesion molecule NCAM, imposing central immune tolerance that historically complicated vaccine development against serogroup B meningococcal disease.^503^ The *E. coli* K1 capsule is required for survival in the bloodstream and for the bacteremic phase preceding invasion of the blood-brain barrier, and the K1 locus has independently emerged in at least four *E. coli* phylogroups, underscoring its selective advantage during invasive infection.^430^ Similarly, heparosan (*E. coli* K5) and chondroitin-like (*E. coli* K4) capsules mimic mammalian glycosaminoglycans, exploiting immunological self-tolerance to increase resistance to complement and serum-mediated killing in extraintestinal pathogenic strains.^432^

### 8.4 Capsule Therapeutic Targets

#### 8.4.1 Capsule Antibiotic Targets

The capsule does not present a viable target for conventional antibiotics because its biosynthesis is dispensable for viability in vitro, and inhibiting capsule production would reduce virulence rather than kill the bacterium, an anti-virulence rather than bactericidal strategy. The most developed approach to therapeutically exploiting the capsule is the use of phage-encoded polysaccharide depolymerases, enzymes that catalytically degrade specific CPS to expose the underlying bacterial surface. In *K. pneumoniae*, phage capsule depolymerases display extraordinary specificity, with distinct enzyme families targeting different capsule types through either intra-chain or exo-chain cleavage mechanisms; 58 characterized depolymerases have been described to date, with structural data revealing conserved substrate-recognition and phage-tethering domains alongside significant catalytic diversity.^504^ Depolymerases sensitize encapsulated *K. pneumoniae* to complement-mediated killing, phagocytosis, and antibiotic action, and recombinant depolymerases show efficacy in murine infection models; however, their extreme serotype specificity means that matched enzyme–capsule type pairing would be required for clinical use, necessitating rapid capsule typing as a prerequisite for treatment.^504^ The breadth of capsule types across clinically relevant species, particularly in *K. pneumoniae*, *Acinetobacter baumannii*, and hypervirulent strains, thus presents a significant barrier to broad clinical deployment of depolymerase-based strategies and has driven interest in engineering broader-spectrum variants or inhibitor cocktails.^505^

#### 8.4.2 Capsule Vaccine Targets

Capsules are not essential for bacterial growth under standard conditions, thus, they are not the primary targets for conventional bactericidal antibiotics; instead, the therapeutic utility of capsules is realized through vaccine development, where CPS serves as the antigen in some of the most clinically successful vaccines in history, and through enzymatic approaches that degrade the capsule to restore immune and antibiotic susceptibility.^506,507^ The same chemical properties that make capsular polysaccharides (CPSs) effective virulence factors, surface exposure, conservation within serotypes, and ability to elicit opsonizing antibodies, also make them attractive vaccine antigens. The central immunological principle underlying CPS-based vaccines is that antibodies directed against the capsule can restore opsonophagocytosis that the capsule itself suppresses, converting a survival advantage to a vulnerability. However, translating this principle into broadly protective vaccines has required overcoming two recurring chemical obstacles: the T-cell-independent nature of pure polysaccharide antigens, which fail to generate immunological memory in infants, and the serotype specificity of anti-capsule antibodies, which limits cross-protection across the enormous structural diversity of capsule types described in Section 8.^508,509^ For Group B *Streptococcus*, correlates of protection for CPS conjugate vaccines targeting serotypes Ia, Ib, II, III, and V have been defined, providing a clearer regulatory pathway and accelerating several clinical programs targeting neonatal disease.^510^

Pure polysaccharide vaccines were the first generation of CPS-based immunizations and remain in use for certain populations. The 23-valent pneumococcal polysaccharide vaccine (PPSV23) covers the serotypes responsible for most of the invasive pneumococcal disease in adults and elicits protective IgM and IgG responses in immunocompetent individuals through a T cell-independent mechanism.^508,511^ Similarly, the quadrivalent meningococcal polysaccharide vaccine (MPSV4) targets *N. meningitidis* serogroups A, C, W, and Y and is effective in adults,^512^ although both vaccines are poorly immunogenic in children under two years of age and do not induce durable immunological memory.^509^

Conjugate vaccines overcome the T-cell independence problem by covalently linking CPS to an immunogenic carrier protein, such as diphtheria toxoid CRM197, tetanus toxoid, or the OMP complex of *N. meningitidis*. Conjugation converts the T-cell-independent polysaccharide response into a T-cell-dependent one, enabling affinity maturation, isotype switching to IgG, and the formation of long-lived memory B cells.^506^ The *H. influenzae* type b (Hib) conjugate vaccine, introduced in the late 1980s, targets the polyribose ribitol phosphate capsule described in Section 8.1 and has essentially eliminated invasive Hib disease in vaccinated populations, representing one of the most successful applications of glycoconjugate chemistry in medicine.^513,514^ The 7-valent pneumococcal conjugate vaccine (PCV7) and its expanded successors PCV13 and PCV15/20 target the most prevalent invasive *S. pneumoniae* serotypes; widespread implementation has dramatically reduced rates of bacteremia, meningitis, and otitis media in children, with additional herd protection extending to unvaccinated adults.^515,516^ The most recent iteration, PCV21, expands coverage further but its deliberate omission of certain serotypes creates coverage gaps relevant to Indigenous and lower-income country populations where serotype 4 has re-emerged.^517^ Conjugate vaccines against *N. meningitidis* serogroups A, C, W, and Y and variants have reduced meningococcal disease globally; however, emerging variants still require surveillance and development of additional vaccines.^518^ A new pentavalent vaccine that introduces serogroup B (Bexsero, Trumenba) was approved by the FDA.^519^ These conjugate vaccines circumvent the self-tolerance problem posed by the PSA capsule described in Section 8.2.2 by incorporating OMPs rather than the capsule alone.

Despite these successes, the serotype coverage remains a fundamental limitation. Capsule replacement, the expansion of nonvaccine serotypes following selective pressure from conjugate vaccines, has been documented for *S. pneumoniae* following PCV introduction and represents an ongoing challenge for vaccine design.^520^ The extreme serotype diversity of *K. pneumoniae* (at least 77 K-types) and *E. coli* (approximately 80 K-antigens) has so far precluded comprehensive conjugate vaccine coverage for these pathogens, driving interest in conserved subcapsular antigens and broadly reactive approaches.^521^ Several CPS conjugate formulations targeting the most prevalent *K. pneumoniae* capsular types, particularly K1, K2, K47, and K64, which predominate among hypervirulent clinical isolates, have demonstrated protection in preclinical models, although no licensed product yet exists.^507,510^ The chemical and structural diversity of bacterial capsules thus remain both a central mechanism of immune evasion and a continuing challenge for the rational design of protective vaccines.

## 9 Overview of the structure and organization of extracellular appendages: Pili, fimbriae, and curli

Surface appendages (Figure 10) represent a structurally and functionally diverse class of bacterial surface structures that extend beyond the OM (in diderm bacteria) or the PG layer (in monoderm bacteria) into the extracellular environment. Unlike the relatively static protein and lipid components of the cell envelope discussed above, these filamentous structures are dynamic, often subject to phase and antigenic variation, and represent primary mediators of host–pathogen interaction at the earliest stages of infection. The major categories covered here are chaperone–usher fimbriae, type IV pili and their relatives, sortase-assembled pili in monoderm bacteria, conjugative pili, and the functional amyloid curli fibers.

**Figure 10:**
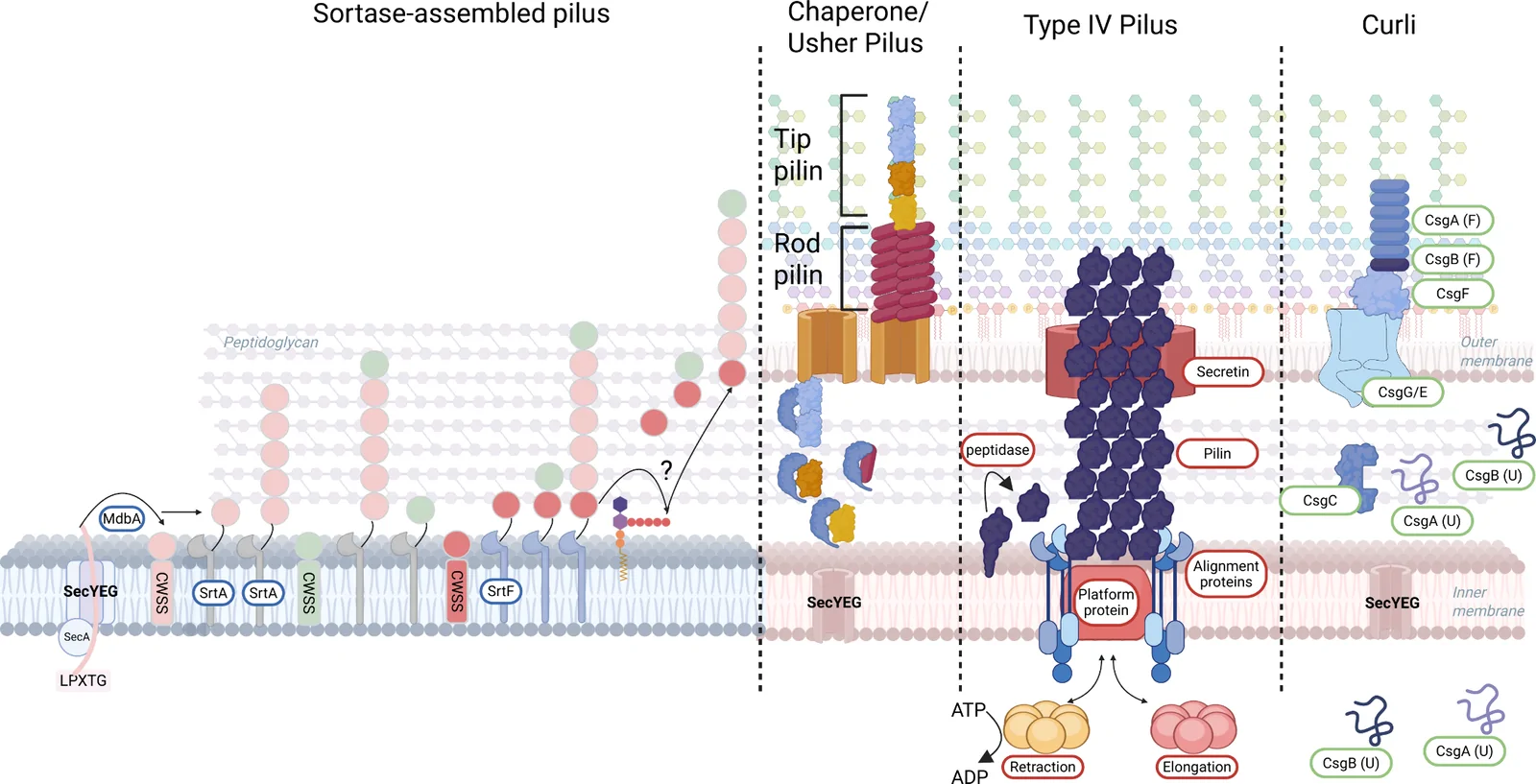
**Assembly mechanisms of bacterial surface appendages.** Four classes of surface appendages are depicted, each employing a distinct biogenesis strategy (Conjugative pili are assembled with T4SS which is shown in Figure 11). **Sortase-assembled pili** (left) are found in monoderm bacteria and built through a biphasic covalent mechanism. Pilin precursors carrying a C-terminal cell wall sorting signal (CWSS) are retained at the membrane, where a pilus-specific sortase (SrtA) catalyzes sequential transpeptidation reactions that covalently crosslink individual pilin subunits into a growing polymer, proceeding from tip to base. The sortase SrtF anchors the completed pilus to the basal pilin. Attachment to the PG cell wall differs between species. **Chaperone/usher (CU) pili** (second panel) are assembled in diderm bacteria via a periplasmic chaperone and OM usher platform. Pilin subunits are translocated across the IM by the Sec translocon and captured by a periplasmic chaperone, which maintains them in a foldedstate and delivers them to the OM usher. The usher catalyzes donor-strand exchange between successive pilin subunits, assembling tip and rod pilin subunits into a growing fiber that is extruded through the usher pore to the cell surface. **Type IV pili** (third panel) are dynamic filaments found in both monoderm and diderm bacteria. Prepilins are inserted into the IM by the Sec translocon and processed by a dedicated peptidase. The platform protein and alignment proteins at the IM coordinate subunit addition driven by an elongation ATPase, while a retraction ATPase powers depolymerization, both using ATP hydrolysis. The secretin forms the OM pore through which the pilus is extruded. **Curli fibers** (right panel) are functional amyloids assembled by a dedicated nucleation-precipitation mechanism. CsgA and CsgB are synthesized as unfolded monomers in the cytoplasm, translocated across the IM by Sec, and exported to the cell surface through the OM pore formed by CsgG/E, assisted by the accessory protein CsgF. CsgB acts as a nucleator that templates the polymerization of CsgA into amyloid fibers on the cell surface. CsgC prevents premature intracellular aggregation of CsgA and CsgB in the periplasm. Created in https://BioRender.com.

### 9.1 Chaperone–Usher Pili

The chaperone/usher (CU) pathway (Figure 10) is the most widespread mechanism by which Gram-negative bacteria assemble adhesive surface filaments called pili or fimbriae.^522,523^ CU pili are found predominantly in Enterobacteriaceae and related diderm pathogens, where they mediate attachment to host tissues as a critical early step in infection. The best-characterized examples are type 1 pili, which enable uropathogenic *E. coli* (UPEC) to colonize the bladder epithelium, and P pili, which mediate kidney colonization in pyelonephritis.^522^ Within UPEC alone, multiple distinct CU systems are expressed, including F1C pili mediating binding to renal tubular cells, S fimbriae targeting sialic acid-containing receptors in the urinary tract and meninges, and Dr/Afa fimbriae recognizing decay-accelerating factor on uroepithelial and endothelial cells.^524^ CU fimbriae extend well beyond uropathogens: colonization factor antigen I (CFA/I) and related CS fimbriae are assembled by this pathway in enterotoxigenic *E. coli* (ETEC), where they mediate adhesion to the small intestinal epithelium and are essential for diarrheal disease.^525,526^ CU systems have also been identified in *H. influenzae*, *K. pneumoniae*, *Salmonella* species, and *A. baumannii*, underscoring their broad distribution and importance across Gram-negative pathogens.^523^

CU pili are composite structures built from a series of protein subunits, each sharing an incomplete immunoglobulin-like fold that lacks the seventh C-terminal β-strand. The assembled filament consists of two morphologically distinct regions: a rigid, helical rod composed of hundreds to thousands of copies of the major pilin subunit (FimA in type 1 pili, PapA in P pili), and a short, flexible tip fibrillum that terminates in a tip adhesin responsible for receptor binding (FimH in type 1 pili, PapG in P pili).^522,527^ Between the rod and the adhesin, several minor subunits occupy defined positions: in type 1 pili, FimF and FimG form the junction between rod and tip, while in P pili, PapE, PapF, and PapK occupy equivalent linker positions.^528^ All subunits are incorporated through a mechanism called donor strand complementation, in which the N-terminal extension of each incoming subunit donates a β-strand to complete the immunoglobulin fold of its neighbor, creating an interlocked polymer.^529,530^ This non-covalent but highly stable interstrand bonding gives the rod its mechanical resilience and its capacity to uncoil under tensile stress and recoil upon relaxation, a property proposed to protect bacteria from detachment under shear flow in the urinary tract.^522^

Crystal structures of tip complexes, including FimH-FimG-FimF-FimC at 2.7 Å and the equivalent PapD-PapA system, reveal the geometry of this donor strand exchange and the conformational changes each subunit undergoes upon interlocking with its neighbor.^528,531,532^ In the assembled rod, hundreds to thousands of major pilin subunits are wound into a right-handed superhelix stabilized entirely by these non-covalent interstrand contacts, giving the filament both mechanical resilience and the capacity to uncoil reversibly under tensile stress.^522^ The tip adhesin domains adopt a conserved elongated β-barrel jelly-roll fold that presents the receptor-binding site at its apical surface, a scaffold shared across structurally characterized CU adhesins including FimH, PapG, GafD, and DraE despite minimal sequence identity.^533,534^

#### 9.1.1 Type IV Pili

Type IV pili (T4P) (Figure 10) are highly dynamic surface filaments found across a broad phylogenetic range of both Gram-negative and Gram-positive bacteria as well as archaea, making them one of the most widely distributed bacterial surface organelles known.^535,536^ T4P mediate a remarkable diversity of cellular functions, including adhesion to host tissues and abiotic surfaces, twitching motility driven by cycles of filament extension and retraction, natural competence for DNA uptake, bacteriophage infection, and biofilm formation.^536,537^ T4P are divided into three subclasses: type IVa (T4aP), type IVb (T4bP), and type IVc (T4cP, also called Tad or tight adherence pili), which differ in pilin subunit size, the extent of N-terminal processing, assembly machinery composition, and functional outputs, though all share the defining features of the superfamily.^535,537,538^ In pathogenic bacteria, T4P are critical virulence determinants: *N. gonorrhoeae* and *N. meningitidis* use T4P to colonize mucosal epithelium and resist phagocytosis; *P. aeruginosa* T4P mediate initial attachment to airway epithelium and are required for establishing chronic lung infections in cystic fibrosis patients; *Vibrio cholerae* toxin-coregulated pilus (TCP) is essential for intestinal colonization and is among the most potent colonization factors known; and in enteropathogenic *E. coli* (EPEC), the bundle-forming pilus (BFP) mediates microcolony formation on the intestinal epithelium.^535,536^ T4cP (Tad pili) are found in an equally broad range of organisms including *Aggregatibacter actinomycetemcomitans*, *Caulobacter crescentus*, *Myxococcus xanthus*, and *Vibrio vulnificus*, where they mediate surface adhesion, biofilm formation, cell–cell aggregation, and surface sensing.^538–540^

The T4P filament is a homo- or hetero-polymeric helical assembly built primarily from thousands of copies of the major pilin subunit, designated PilA or PilE depending on the organism, along with minor pilins that occupy the filament tip and play roles in initiating assembly and mediating adhesion.^536,541^ In T4aP and T4bP systems, each pilin subunit consists of a conserved hydrophobic N-terminal α-helix (α1) that forms the core of the filament, packed against a globular C-terminal head domain containing a four-stranded antiparallel β-sheet and variable surface-exposed loops that determine receptor specificity and antigenicity.^541,542^ The N-terminal α1 helix partially unfolds at a conserved segment near the helix base, and this flexible region accommodates the tight packing geometry of subunits in the filament interior.^542,543^ T4cP Flp pilins are structurally distinct: they lack the globular C-terminal β-sheet head domain entirely and are instead purely α-helical, producing a thinner and structurally simpler filament than those of T4aP and T4bP systems.^538^ n all three subclasses, filament stability arises from hydrophobic interactions among the N-terminal helices of adjacent subunits rather than from the intermolecular β-strand exchange that stabilizes CUP pili.^538,541,544^

The atomic structure of individual pilin subunits has been determined for multiple T4P systems. The *N. gonorrhoeae* PilE pilin crystal structure established the canonical T4aP pilin fold: a long N-terminal α-helix packing against a globular C-terminal domain containing a disulfide-bonded D-region loop that is critical for receptor recognition and immune evasion through antigenic variation.^541^ Cryo-EM reconstructions of intact filaments from *N. gonorrhoeae* and *P. aeruginosa* revealed how subunits pack in the assembled fiber, with the hydrophobic N-terminal helices forming a tight apolar core and the globular C-terminal domains coating the outer surface and presenting variable loops for host interactions.^542,543^ A structure of *M. xanthus* T4aP revealed that species-specific differences in pilin length and intersubunit contact geometry produce measurable differences in filament mechanical properties, linking atomic-level structural variation to the macroscopic function of motility on varied surfaces.^544^ For T4cP, the first intact in situ structure of the complete Tad pilus machine was resolved in *C. crescentus* by cryo-ET and subtomogram averaging, revealing that the Tad filament is built from purely α-helical PilA subunits lacking the globular C-terminal head domain of T4aP and T4bP pilins, and capturing multiple assembly intermediates of the nanomachine within the intact cell envelope.^538^

#### 9.1.2 Sortase-Assembled Pili in Monoderm Bacteria

Sortase-assembled pili (Figure 10) are surface appendages found exclusively in Gram-positive bacteria, where they serve as critical mediators of adhesion, colonization, biofilm formation, and immune evasion.^545,546^ This class of pili is named after the transpeptidases (sortases) that polymerize the pilus (class E) and attach the pilus to the cell wall (a housekeeping sortase of class F). They are broadly distributed across clinically significant pathogens and commensals, including *S. pyogenes*, *S. agalactiae*, *S. pneumoniae*, *E. faecalis*, *E. faecium*, *Corynebacterium diphtheriae*, *Actinomyces oris*, and *Bacillus cereus*, as well as gut commensals such as *Lactobacillus* and *Bifidobacterium* species.^545,547–550^ In addition, single bacterial species have more than one type of sortase-assembled pili. There are two classes of sortase pili: heterotrimeric and heterodimeric. Heterotrimeric sortase pili have one protein forming the base attachment to the cell wall (basal pilin), the polymerized subunit forming the rod (the major or backbone pilin), and the tip protein (minor pilin adhesins). Heterodimeric lack a basal pilin but have tip and major pilin proteins. Although bacteria all have this architecture, the individual subunits differ in sequence and structure. Most basal, major, and minor pilins have an immunoglobulin (Ig)-like fold β-sandwich domain named collagen adhesin (Cna) and are divided into two subfamilies CnaA and B. All Cna folds have isopeptides; however, the number of Cna domains and the placement of the isopeptide bonds differ. The structure of the *S. pyogenes* major pilin has two Cna domains, each is stabilized by an intramolecular isopeptide bond between a lysine and an asparagine that contributes to the stability of the individual subunits.^551,552^ Each major pilin is connected to the next by an intermolecular isopeptide bond between a lysine in one subunit with the carboxyl terminus of another subunit.^551^ In contrast, the *S. pneumoniae* major pilin has four domains (D1-4) each with an intermolecular isopeptide and an intermolecular isopeptide bond between D1 and D4.^553^ The adhesion subunits have a binding domain that adopts a classic α/β open-twisted (or para-Rossmann) fold (also termed von Willebrand factor type A (vWFA) fold). The vWFA domains typically have a metal ion-dependent adhesion site (MIDAS; D-x-S-x-S-T-D) and bind extracellular matrix proteins such as mucin, laminin, collagen, fibrinogen, and fibronectin.^554,555^ The vWFA domain is followed by a series of Can domains. In *Lactobacillus rhamnosus* GG, the vWFA is followed by a CnaA domain and three CnaB domains.^555^ In contrast, the minor pilin of *S. pneumoniae* has four domains: the vWFA domain followed by one CnaB and two CnaA domains.^554^

#### 9.1.3 Conjugative Pili

Conjugative pili are surface appendages produced by donor bacteria that mediate the initial cell–cell contact required for bacterial conjugation, the direct transfer of DNA between cells, making them the primary drivers of horizontal gene transfer across bacterial populations.^556,557^ They are encoded by conjugative plasmids and integrative conjugative elements (ICEs) found across a wide range of Gram-negative and Gram-positive bacteria, with the F plasmid-encoded system of *E. coli* serving as the paradigm.^557,558^ The conjugative pilus is an extracellular appendage of the type IV secretion system (T4SS), which spans the entire cell envelope and provides the channel through which the DNA-protein transfer complex is delivered to the recipient cell; the T4SS machinery itself is discussed in detail in section 10.0.4.^556,559^ Because conjugative plasmids frequently carry antibiotic resistance and virulence genes, conjugative pili represent a central mechanism in the dissemination of antimicrobial resistance across bacterial communities.^560,561^

Conjugative pili are thin, flexible tubular filaments approximately 8–9 nm in outer diameter with a hollow lumen of approximately 2.5–3 nm.^560–562^ The filament is built from a single major pilin species, TraA in F-type systems and VirB2 in *Agrobacterium tumefaciens* T-pili, that is processed from a propilin precursor synthesized with an unusually long leader peptide of approximately 50 amino acids.^562,563^ The mature TraA pilin is approximately 64 amino acids long and folds into a purely α-helical structure comprising three helices (α1, α2, α3), with no β-sheet component.^562^ A defining and unexpected structural feature revealed by cryo-EM is that pilin subunits assemble with phospholipid molecules at a strict 1:1 stoichiometric ratio:^561,562^ mass spectrometry confirmed these are phosphatidylglycerol (PG) species (primarily PG 32:1, PG 33:1, and PG 34:1) selectively incorporated from the IM, with PG head groups exposed to the electronegative lumen and acyl chains buried between adjacent helical segments.^560^ Mutations in the phospholipid binding sites (Y37V and A28F in pED208) abolish pilus assembly, demonstrating that lipid incorporation is structurally essential and not incidental.^562^ The helical symmetry of the assembled filament varies between systems: F-type and pED208-type pili display five-fold rotational symmetry (five-start helical filaments), while the pKpQIL pilus from carbapenem-resistant *K. pneumoniae* forms a one-start.^561,562^ An additional structural variant is the H-pilus of IncH plasmids, in which the TrhA pilin undergoes covalent cyclization connecting the N- and C-termini following cleavage of the last five residues; this cyclization is essential for pilus biogenesis and efficient plasmid transfer.^560^

#### 9.1.4 Curli Fibers

Curli (Figure 10) are functional amyloid fibers that form the major proteinaceous component of the extracellular matrix in biofilms produced by many Gram-negative bacteria. They were first identified in *Salmonella* and *E. coli* and are phylogenetically widespread across at least four bacterial phyla, including Proteobacteria and Bacteroidetes, with curli genes detectable in environmental, clinical, and commensal metagenomes.^564^ Within the biofilm matrix, curli mediate initial adhesion to abiotic and biotic surfaces, provide structural cohesion by forming interbacterial bundles, and contribute to the mechanical stability and stress resistance of the biofilm community.^565,566^ A notable biological feature is the capacity of curli fibers for cross-species seeding: curli subunit homologues from *E. coli*, *Salmonella*, and *Citrobacter* cross-seed one another *in vitro* and support interspecies fiber formation in mixed-species biofilms, a process facilitated by the high conservation of the curlin repeat architecture.^567,568^

In *E. coli*, the curli system is encoded by two divergently transcribed operons, *csgBAC* and *csgDEFG*, controlled by the master transcriptional regulator CsgD and the stationary-phase sigma factor σS.^569^ CsgA is the major structural subunit and principal polymerizing component; CsgB is a minor subunit that serves as the membrane-anchored nucleator of CsgA amyloidogenesis; both are approximately 13 kDa polypeptides.^566^ Each subunit carries a 20-residue N-terminal signal peptide for Sec-dependent export, followed by a 22-residue N-terminal targeting sequence (N22) that directs the subunit to the OM secretion machinery (CsgG), and an amyloidogenic core composed of five imperfect pseudo-repeat sequences of approximately 22 amino acids (R1–R5); each repeat forms a strand-arc-strand β-arch unit built from two motifs flanked by conserved Asn and Gln residues.^566,568^ Cryo-EM analysis combined with ΑFold2 modeling of CsgA established that the folded subunit adopts a left-handed β-solenoid, and solid-state NMR had previously shown that curli fibers do not adopt the in-register parallel β-sheet architecture of pathological amyloids.^568,570^ In the assembled protofibril, successive CsgA subunits stack head-to-tail via seamless β-solenoid augmentation with pure translational symmetry and no helical twist, producing a fiber with 4.8 Å inter-repeat spacing, approximately 20 Å width, and a half-unit stagger between opposing β-strands.^570^ CsgB adopts a structurally equivalent β-solenoid compatible with CsgA fiber extension; the lower intrinsic polymerization propensity of CsgB compared to CsgA is associated with degenerate residues in its C-terminal repeats R4 and R5.^568,571^ High-speed AFM imaging at single-fiber resolution demonstrated that curli fibers grow from both ends with asymmetric kinetics and display stop-and-go dynamics in which bursts of steady-state elongation alternate with periods of stagnation.^572^

### 9.2 Pili, Fimbriae, and Curli Biogenesis

The assembly of pili, fimbriae, and curli fibers requires dedicated biogenesis machinery that is as structurally diverse as the appendages. Each system must solve the same fundamental problem: transporting structural subunits from the cytoplasm across one or two membranes and assembling them into ordered extracellular structures without premature aggregation or mislocalization. The solutions that have evolved are remarkably varied, ranging from the periplasmic chaperone and OM usher platform used by CU fimbriae, to the dynamic ATPase-powered extension and retraction machinery of type IV pili, to the covalent isopeptide crosslinking catalyzed by dedicated sortases in monoderm bacteria, to the amyloid nucleation and secretion pore system that produces curli fibers. The following subsections describe the biogenesis machinery and assembly mechanisms for each class of surface appendages in turn.

#### 9.2.1 CU Pili Biogenesis

Biogenesis of CU pili (Figure 10) requires two dedicated components: a periplasmic chaperone and an OM usher.^522,527^ The chaperone, exemplified by FimC in type 1 pili and PapD in P pili, adopts a two-domain immunoglobulin-like fold and binds each newly translocated subunit in the periplasm by donating a G1 β-strand to complete the subunit’s incomplete fold, a mechanism called donor strand complementation.^529,532^ This interaction stabilizes the subunit in a high-energy activation state, storing the free energy that will subsequently drive fiber elongation.^527^ Chaperone-subunit complexes are delivered to the OM usher, a 24-stranded β-barrel occluded by an internal plug domain, where subunits are incorporated into the growing fiber through donor strand exchange as described above in Section 9.1.^527,573–575^ The usher coordinates this process through spatially distinct functional domains: the N-terminal domain recruits incoming chaperone-subunit complexes, the β-barrel lumen accommodates the translocating subunit, and the C-terminal domains anchor the elongating fiber on the extracellular face.^574,575^ Assembly is terminated when the capping subunit FimI engages the usher irreversibly, preventing further subunit incorporation.^534^

#### 9.2.2 Type IV Pili Biogenesis

T4P biogenesis (Figure 10) requires the coordinated action of more than a dozen proteins spanning both membranes.^536,537^ Newly synthesized pre-pilins are inserted into the IM by the Sec translocon and processed by a dedicated prepilin peptidase (PilD in T4aP/T4bP) that cleaves the short leader sequence and methylates the new N-terminal phenylalanine, a modification essential for filament assembly.^536,576,577^ Processed pilins are then extracted from the IM by the extension ATPase PilB, which forms a cytoplasmic hexameric ring that interacts with the IM platform protein PilC; together these drive pilin polymerization at the base of the growing filament, threading it through the OM secretin pore formed by the PilQ multimer.^537,578^ Retraction is powered by the PilT ATPase, which drives depolymerization at the same IM interface, generating tensile forces sufficient to pull attached objects and propel the cell across surfaces.^536,579^ The alignment complex, comprising PilM, PilN, PilO, and PilP in *P. aeruginosa*, physically bridges the IM and OM components and is required for efficient assembly.^537,578^ T4cP biogenesis follows the same general logic, Flp pilins are processed by a dedicated prepilin peptidase prior to incorporation, and a single assembly ATPase (TadA) drives polymerization, but differs in that the periplasmic conduit is formed by the RcpC dodecamer rather than a canonical alignment complex, the OM channel is the phylogenetically distinct RcpA secretin rather than PilQ, and the additional OMPs RcpB and TadD are required for proper secretin assembly and stability.^538,580,581^ The in situ cryo-ET structure of the *C. crescentus* Tad machine further revealed the stepwise assembly process of the complete nanomachine within the intact cell envelope, providing the first architectural framework for understanding how all core Tad components are spatially organized during pilus biogenesis.^538^

#### 9.2.3 Sortase-Assembled Pili Biogenesis

Sortase-assembled pilus biogenesis (Figure 10) proceeds through a two-stage biphasic mechanism.^545,547^ Pilin precursors are first translocated across the cytoplasmic membrane by the Sec machinery, then properly folded by a dedicated oxidative protein-folding machine and retained at the membrane via their C-terminal cell wall sorting signal (CWSS), composed of an LPXTG motif followed by a hydrophobic domain and a cluster of positively charged residues.^545^ In the first stage of assembly, a pilus-specific class C sortase polymerizes pilin subunits by catalyzing sequential isopeptide bond formation at a membrane-associated platform: the enzyme cleaves the LPXTG motif to form a thioester-linked acyl-enzyme intermediate, which reacts with the lysine within the pilin motif of the next incoming subunit.^582,583^ It is assumed that polymerization is initiated by first crosslinking the tip adhesin pilin to the major rod pilin, building the pilus from tip to base (the “tip first rule”).^545,548^ For heterotrimeric pili, polymerization is terminated when the base pilin outcompetes the shaft pilin for the sortase thioester intermediate.^550,584^ For heterodimeric pili, termination and pilus length are thought to be determined by the housekeeping sortase that attaches the major pilin to the cell wall.^583^ In the second stage, the completed pilus polymer is transferred to the PG cell wall through transpeptidation catalyzed by either the housekeeping sortase or the pilus-specific sortase itself.^585^ The mechanism of cell wall anchoring varies across systems: in *S. aureus* the housekeeping sortase SrtF links the pilus polymer to newly synthesized PG via lipid II,^105^ while in *E. faecalis* the Ebp pili are anchored directly to un-cross-linked cell wall independently of newly synthesized PG,^586^ suggesting that the cell wall anchoring step is more mechanistically diverse across Gram-positive bacteria than previously appreciated.^545^ A recently discovered regulatory layer in *Actinobacteria* involves SafA, a conserved transmembrane protein that directly interacts with the housekeeping sortase and prevents its cleavage by signal peptidase LepB2, thereby maintaining sortase membrane homeostasis and ensuring proper pilus assembly.^587^

#### 9.2.4 Conjugative Pili Biogenesis

Conjugative pilus biogenesis is driven entirely by the T4SS machinery encoded on the conjugative element, and the pilus is therefore an extracellular appendage of that secretion system rather than an independent assembly pathway; the molecular details of the T4SS are covered in Section 10.1.3.^556,558^ PropilinTraA insertion into the IM is Sec-independent and instead is inserted by the F plasmid-encoded transmembrane protein TraQ.^563,588^ The 5.5 kDa leader peptide is then cleaved by the periplasmic signal peptidase LepB, after which the new N-terminus of the mature 7 kDa pilin is acetylated by the F plasmid-encoded TraX.^563,589^ Phospholipid incorporation into the filament occurs concurrently with polymerization, though the mechanism by which PG species are selectively recruited into the growing pilus at 1:1 stoichiometry remains to be elucidated.^561,562^ A distinctive feature of conjugative pili is their dynamic behavior: live-cell imaging has shown that F-pili undergo spontaneous cycles of extension and retraction, with extension and retraction accompanied by rotation about the long axis of the filament; this dynamic sampling of the environment increases the probability of contact with recipient cells, and once contact is established, the flexible pilus maintains the mating pair against shear forces in liquid media.^590^ Retraction is thought to draw donor and recipient cells into close enough contact to establish the stable mating pair required for DNA transfer.^556,558^ Unlike CUP pili, T4P, or sortase-assembled pili, conjugative pili require no dedicated OM usher, no periplasmic chaperones for pilin stabilization, and no independent assembly ATPase, all these functions are integrated within the T4SS complex.^557^

#### 9.2.5 Curli Fibers Biogenesis

Curli biogenesis (also known as type VIII secretion) begins with the synthesis of CsgA and CsgB with an N-terminal signal peptide. CgsA and CgsB are then translocated to the periplasm via the SecYEG translocon (Figure 10), where their signal peptides are cleaved.^566^ In the periplasm, the 11 kDa protein CsgC acts as a sub-stoichiometric amyloid inhibitor that maintains CsgA and CsgB in a soluble, non-β-sheet conformation, preventing toxic premature aggregation prior to secretion.^591^ Secretion of CsgA and CsgB across the OM is mediated by CsgG, a nonameric β-barrel diffusion-driven peptide channel.^566,592^ CsgE forms a nonameric periplasmic cap that closes the cytoplasmic vestibule of the CsgG pore, providing substrate specificity by directly engaging CsgA through its positively charged head region (centered on R47) while preventing non-specific protein passage.^593,594^ On the extracellular surface, the accessory protein CsgF is essential for CsgB surface attachment and nucleator activity: in *csgF* null mutants, secreted CsgA is released as soluble monomers rather than assembling into cell-associated fibers, because CsgB fails to localize to and remain on the OM.^595^ Once CsgB is surface-anchored and activated by CsgF, an interaction that depends on CsgB’s C-terminal repeats, it rapidly templates CsgA into fibers, initiating nucleation-dependent polymerization in a head-to-tail manner that propagates through seamless continuation of the β-arcade.^568,571,593,594,596^

### 9.3 Role of Pili, Fimbriae, and Curli in Pathogenesis

Surface appendages are among the most direct mediators of host–pathogen interaction, functioning as the first points of contact between a bacterium and its target tissue. The tip adhesins determine host cell tropism, establish intimate attachments required for colonization, and in many cases, trigger downstream signaling cascades in host cells that facilitate invasion or immune evasion. Curli fibers contribute to biofilm formation on host surfaces and are potent activators of innate immune pathways. The pathogenic contributions of these structures are not limited to adhesion: surface appendages also participate in horizontal gene transfer, motility, biofilm development, and subversion of complement and phagocytic clearance. The following subsections describe the specific pathogenic roles of each appendage class, with emphasis on the molecular interactions that contribute to infection.

#### 9.3.1 CU Fimbriae in Pathogenesis

FimH mediates binding to mannosylated glycoproteins on uroepithelial cells through a mechanosensitive catch bond: tensile force from urine flow induces a conformational change in the FimH lectin domain that paradoxically strengthens adhesion, allowing uropathogenic *Escherichia coli* (UPEC) to resist voiding. The allosteric mechanism couples the lectin domain to the pilin domain such that force-induced separation of the two domains shifts FimH to a high-affinity conformation for its mannose receptor.^528^ Genetic deletion of *fimH* abolishes bladder colonization in murine models.^597^ P fimbriae mediate binding to globoside receptors in the upper urinary tract via PapG, facilitating pyelonephritis; the structural basis of PapG and GalNAcβ1-3Galα1-4Galβ1-4Glc recognition was determined using crystallography.^598^

Beyond UPECs, CUP fimbriae are virulence factors across a wide range of enterobacterial pathogens. Dr fimbriae of *E. coli* bind decay-accelerating factor (DAF/CD55) on uroepithelial cells, and this interaction has been shown to trigger lipid-raft-mediated invasion.^599^ Mrp fimbriae of *Proteus mirabilis* facilitate catheter-associated urinary tract infections.^600^ *K. pneumoniae* type 3 fimbriae mediate biofilm formation on host tissues and indwelling devices through binding to collagen IV and fibronectin.^601^ The MrkD tip adhesin of type 3 fimbriae adopts a lectin fold structurally unrelated to FimH, indicating that CUP pili have recruited diverse adhesin architectures to engage different host receptors.^602,603^ CU fimbriae of enterotoxigenic *E. coli* (ETEC) mediate adhesion to small intestinal epithelium and are major determinants of diarrheal disease.^604,605^

#### 9.3.2 Type IV Pili in Pathogenesis

T4P contributes to pathogenesis through two mechanistically distinct modes: direct receptor-mediated adhesion that establishes initial host contact and force-generating retraction that triggers intracellular signaling cascades facilitating invasion, immune modulation, and biofilm development. T4P mediates initial surface attachment and, through retraction, generates mechanical force that triggers signaling cascades in host cells. In *N. gonorrhoeae* and *N. meningitidis*, pilus retraction activates Rac1 and cortactin-dependent actin rearrangements in epithelial cells, facilitating bacterial engulfment.^606^ The tip-localized minor pilin PilC directly mediates binding to epithelial cell receptors.^607^ Antigenic variation of the major pilin subunit PilE through a G-quadruplex DNA-directed recombination mechanism generates extensive structural diversity at the surface, constituting a major mechanism of immune evasion and persistence in *N. gonorrhoeae* infection.^1000^

In *P. aeruginosa*, the pilus-associated protein PilY1 is required for bacterial adherence to mucosal epithelial tissue and mediates binding to basolateral host cell surfaces.^608^ T4P are required for initial colonization in cystic fibrosis,^609^ where *P. aeruginosa* T4aP engage glycolipid receptors on the apical surface of airway epithelial cells.^610^ Twitching motility, driven by T4P retraction, directs the formation of structured biofilms central to chronic infection.^611^ The bundle-forming pilus (BFP) of enteropathogenic *E. coli* (EPEC) mediates bacterial autoaggregation and microcolony formation on intestinal epithelial cells, which is required for the attaching and effacing lesion.^612,613^ The toxin-coregulated pilus (TCP) of *V. cholerae* is both the primary intestinal colonization factor and the receptor for the CTXφ bacteriophage that encodes cholera toxin, linking colonization factor biogenesis directly to the acquisition of the primary virulence determinant.^614^

T4cP (Tad pili) contributes to pathogenesis primarily through mediating irreversible surface adhesion and biofilm initiation rather than dynamic retraction-based signaling. In *A. actinomycetemcomitans*, Tad pili are required for the initial tight adhesion to surfaces that precede microcolony formation and biofilm development, and clinical isolates with deletions in the *tad* locus are severely attenuated for colonization of the oral cavity and virulence in animal models of periodontal infection.^615^ The functional versatility of T4P across pathogenic bacteria, from generating mechanical signals that remodel host actin to serving as the entry receptor for a phage-borne toxin gene, reflects the evolutionary plasticity of a shared assembly platform and underscores the difficulty of predicting the pathogenic mechanism from filament structure alone.

#### 9.3.3 Sortase-Assembled Pili in Pathogenesis

Sortase-assembled pili are critical virulence determinants in the Gram-positive pathogens in which they are found, mediating adhesion to host cells and matrix components, promoting biofilm formation, and in several cases contributing directly to immune evasion. In *S. agalactiae* (Group B streptococcus), pili mediate adhesion to respiratory epithelial cells under physiological flow conditions, promote biofilm formation, and are required for virulence in neonatal infection models.^616,617^ The tip pilin PilA facilitates the interaction of GBS with endothelial cells of the human blood–brain barrier, contributing to blood–brain barrier penetration in neonatal meningitis.^618^ In *C. diphtheriae*, three distinct pilus types (SpaA, SpaD, and SpaH) each mediate adhesion to specific host tissues including pharyngeal, laryngeal, and lung epithelial cells.^547,619^ In *E. faecalis*, the endocarditis and biofilm-associated pilus (Ebp) is an important virulence factor in animal models of endocarditis and catheter-associated urinary tract infection.^620^ Relative to the collagen adhesin Ace, Ebp pili account for most of serum-stimulated fibrinogen adherence in *E. faecalis*, and deletion of the *ebpABC* operon significantly attenuates experimental urinary tract infection.^621^ In oral commensal *A. oris*, type 2 fimbriae mediate coaggregation with streptococci and are essential for oral biofilm development, illustrating the role of sortase-assembled pili beyond canonical pathogenesis.^583^ In *S. pyogenes*, the FCT-region pilus mediates adhesion to multiple host matrix components and promotes pharyngeal colonization and biofilm formation.^622,623^ The broad phylogenetic distribution of sortase-assembled pili across pathogenic and commensal Gram-positive bacteria, and the conservation of their receptor-binding adhesin domain folds across diverse organisms, underscores their fundamental importance as a class of adhesion organelle in the monoderm lifestyle.^545^

#### 9.3.4 Conjugative Pili in Pathogenesis

The pathogenic significance of conjugative pili lies not in direct host interaction but in their role as the extracellular machinery of horizontal gene transfer, which drives the dissemination of resistance and virulence determinants across bacterial communities. Conjugative pili are not virulence factors in the classical sense; they do not mediate adhesion to host tissues, but their role in horizontal gene transfer makes them central to the dissemination of antibiotic resistance determinants and virulence plasmids across bacterial populations. The spread of plasmids encoding extended-spectrum β-lactamases (ESBLs), carbapenemases (KPC, NDM-1, OXA-48), and other resistance mechanisms through conjugative transfer is a primary driver of the global emergence of multidrug-resistant Gram-negative bacterial infections.^624^ The pKpQIL plasmid encoding KPC-2 carbapenemase in *K. pneumoniae* has been shown by cryo-EM to assemble a conjugative pilus with a one-start helical symmetry distinct from F-type pili, making it among the structurally characterized conjugative elements in a clinically critical resistance plasmid.^561^ Additionally, the F pilus serves as the attachment receptor for male-specific bacteriophages including MS2 and M13, which exploit pilus retraction for injection of their genome into the cell.^625^ The *A. tumefaciens* Ti plasmid T4SS represents a remarkable evolutionary adaptation in which the conjugation machinery has been repurposed to transfer oncogenic T-DNA directly into plant eukaryotic cells.^626^ The structural diversity of conjugative pili, including the stoichiometric phospholipid incorporation unique to F-type systems and the distinct helical symmetries of different IncF plasmid pili, suggests that conjugation machinery has undergone substantial evolutionary divergence even as its core function in resistance gene transfer has remained constant.^625^

#### 9.3.5 Curli Fibers in Pathogenesis

Curli fibers mediate adhesion to a broad range of host substrates. CsgA fibers bind fibronectin, laminin, and plasminogen on host cell surfaces, contributing to colonization and biofilm formation on both biotic and abiotic surfaces.^627,628^ Curli are potent innate immune activators. CsgA fibers are recognized by a Toll-like receptor (TLR)­2-dependent mechanism, and amyloid fibrils of both microbial and host origin stimulate TLR2-mediated NOS2 expression in macrophages; an amyloidogenicity-lowering substitution in CsgA reduces this TLR2 agonist activity.^629^ TLR2 alone is not sufficient for full recognition of curli: TLR2 cooperates with TLR1 to recognize curli amyloid fibrils from enterobacterial biofilms, and an inability to produce curli markedly reduces TLR2-dependent responses to intact bacterial cells both in vitro and in vivo.^630^ Curli–DNA complexes within the biofilm matrix activate TLR9 and drive type I interferon production via a STING-dependent pathway, a mechanism proposed to contribute to chronic inflammatory pathology during systemic infection.^630^ Curli production is associated with invasive *Salmonella* and *E. coli* infections. The multicellular behavior of *S. enterica* and *E. coli* is characterized by the co-expression of curli and cellulose, which promotes surface adhesion, biofilm formation, and environmental persistence.^631^ The capacity of curli to simultaneously promote surface adhesion, reinforce biofilm mechanical stability, and activate multiple innate immune receptor pathways positions these fibers as central orchestrators of the host–pathogen interface during enterobacterial biofilm-associated infection.

### 9.4 Pili, Fimbriae, and Curli Therapeutic Targets

#### 9.4.1 Pili, Fimbriae, and Curli Antibiotic Targets

The most clinically advanced strategy focused on pili, fimbriae, and curli targets FimH, the mannose-binding adhesin at the tip of type 1 pili of uropathogenic *Escherichia coli* (UPEC), both as a target for small-molecule inhibitors and as a vaccine antigen.^632,633^

Type 1 pili of UPEC mediate initial attachment to mannosylated uroplakins on bladder epithelial cells through the lectin domain of the tip adhesin FimH, and this interaction is required for bladder invasion and intracellular bacterial community formation that underlies recurrent UTI. Mannosides, orally available β-D-mannose derivatives designed to competitively occupy the FimH mannose-binding pocket, block this adhesion step, keep UPEC extracellular, and sensitize otherwise-resistant strains to antibiotics by preventing tissue invasion and prolonging exposure to urinary drug concentrations.^633^ Type 1 pili also promote UPEC persistence in the gut, oral mannoside administration reduces UPEC gut colonization in murine models, suggesting that FimH inhibition could address both bladder infection and its reservoir simultaneously.^634^ Two independent anti-virulence strategies targeting pili biogenesis itself, pilicides that disrupt chaperone-usher pathway assembly, were characterized *in vitro* against both type 1 and P pili. Additionally, the compound FN075 inhibits curli fiber assembly, classifying it as a dual pilicide and curlicide. However, neither pilicides nor dedicated curli inhibitors have advanced to clinical development.^633^ Curli fibers, the functional amyloid produced by *E. coli* and *Salmonella*, contribute to biofilm matrix integrity through synergistic interactions with extracellular DNA. The eDNA-curli network enhances biofilm robustness, impedes antibiotic penetration, and sequesters antimicrobial peptides, identifying disruption of this amyloid matrix as a mechanistically distinct therapeutic target for biofilm-associated infections.^635^

#### 9.4.2 Pili, Fimbriae, and Curli Vaccine Targets

FimH is the most clinically advanced pilus-based vaccine antigen. The FimCH vaccine (recombinant FimH complexed with its chaperone FimC and formulated with a TLR4 agonist adjuvant) was evaluated in a phase 1 clinical study. All dosages were well-tolerated with no serious adverse events, and vaccination induced both binding and functional anti-FimH antibodies.^632^ Long-term follow-up data from an expanded access study in high-risk patients with carbapenem-resistant Enterobacteriaceae showed approximately a 75% reduction in symptomatic Gram-negative UTI occurrences in the 12-month period following administration.^636^ The current Sequoia Vaccines program has not yet advanced to Phase 2 as of mid-2026, although the Phase 1 results and expanded access data support continued development. Beyond type 1 pili, the RrgA adhesin of pilus islet 1 in *S. pneumoniae* mediates binding to PECAM-1/CD31 on brain microvascular endothelial cells and is immunogenic, providing a mechanistic basis for including pilus antigens in next-generation pneumococcal vaccine formulations targeting invasive disease.^517^ No vaccine targeting curli fibers has entered clinical development, though the immunogenicity of curli during natural infection and the conservation of CsgA across *Enterobacteriaceae* have been noted as features that could be exploited in multiantigen formulations targeting biofilm-forming pathogens.^635^

## 10 Overview of the Structure and Function of Specialized Secretion Systems

Bacterial secretion systems are diverse envelope-spanning machines that export proteins, nucleoprotein complexes, toxins, adhesins, enzymes, and other macromolecules. The current literature recognizes the general Sec and Tat export pathways, together with at least 11 named specialized secretion systems, T1SS through T11SS (Figure 11). It is important to note that the systems are not all equally widespread, equally ancient, or equally well-characterized. However, a fundamental organizational principle in bacterial protein trafficking is that each system is tuned to the structural and energetic constraints of its substrates.

**Figure 11:**
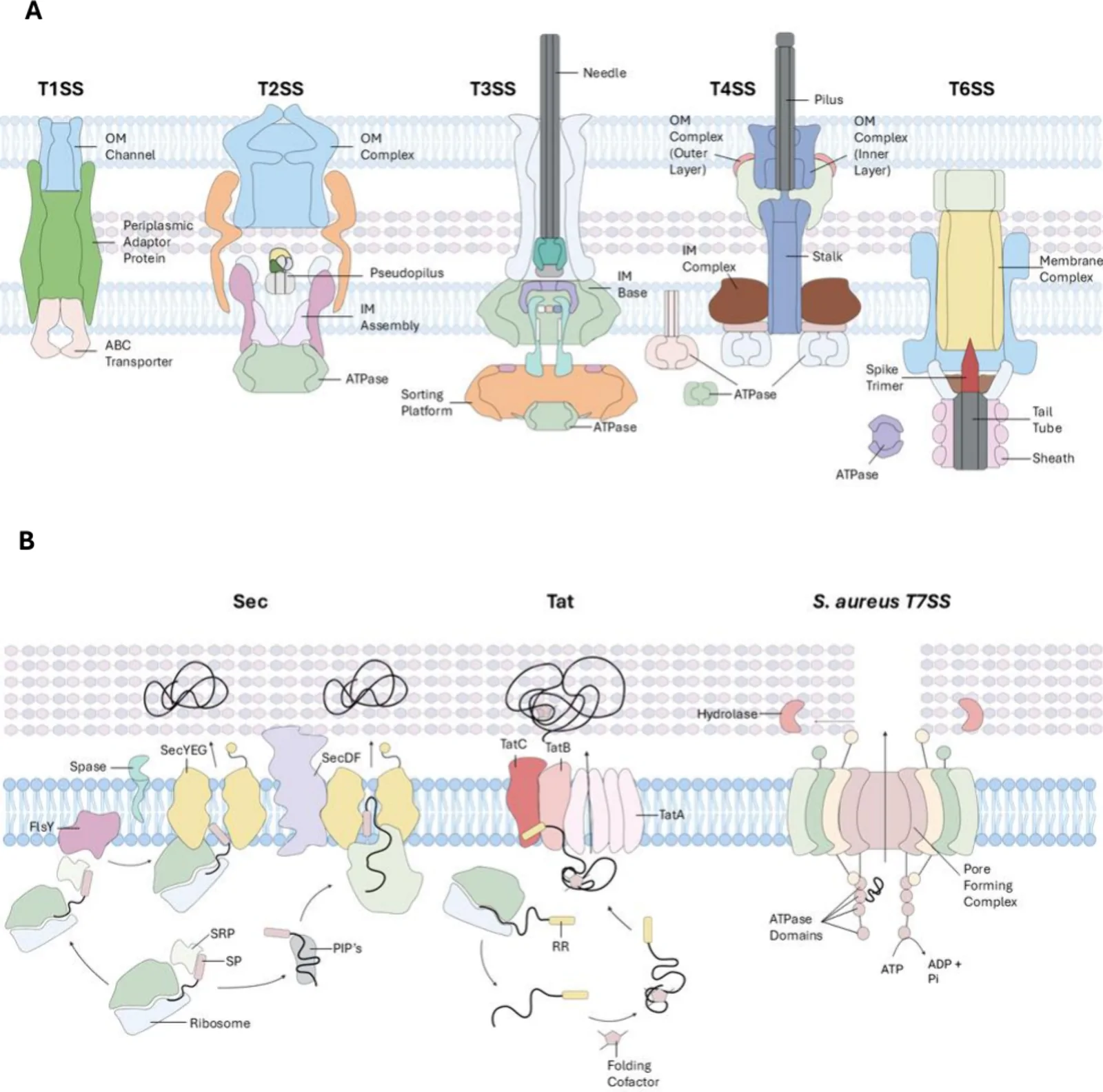
**Secretion mechanisms found in monoderm and diderm bacteria. (A) Diderm secretion systems.** Panel A illustrates five major secretion systems found in diderm bacteria, each spanning the IM (IM), periplasm, and OM (OM) to varying degrees. The Type I secretion system (T1SS) is a streamlined tripartite pump composed of an IM complex (IMC), membrane fusion protein (MFP), and the OM channel, which together form a continuous conduit that bypasses the periplasm entirely. In contrast, the Type II secretion system (T2SS) operates in two steps, first relying on the Sec or Tat pathway to deliver substrates to the periplasm before a dedicated pseudopilus assembly drives them through the secretin pore. The Type III secretion system (T3SS) resembles a molecular syringe, using a basal body anchored in both membranes and a hollow extracellular needle to inject effector proteins directly into eukaryotic host cells. The Type IV secretion system (T4SS), exemplified here by the Agrobacterium VirB/D system, can translocate both proteins and nucleoprotein complexes across membranes, making it central to processes ranging from bacterial conjugation to effector delivery during infection. The Type VI secretion system (T6SS) operates on a fundamentally different contractile mechanism, firing a spring-loaded TssB-TssC sheath that drives the VgrG spike and Gcp tube outward to puncture neighboring cells. **(B) Monoderm secretion mechanisms.** Panel B focuses on the monoderm context, showing how the Sec and Tat pathways deliver substrates across a single membrane into and through the thick PG wall, with the monoderm-specific Type VII secretion system (T7SS) of *S. aureus* shown as an emerging and structurally distinct mechanism that uses ATP hydrolysis to export small virulence proteins lacking classical signal peptides. Created in https://BioRender.com.

In diderm bacteria, secretion is especially complex because substrates often must cross both the IM and the OM. These organisms encode most of the named secretion systems, including the classical T1SS–T6SS and additional systems, namely T8SS, T9SS, T10SS, and T11SS. Monoderm bacteria, on the other hand, have a simpler envelope that lacks an OM. These organisms therefore rely heavily on Sec (unfolded substrates)^23,637^ and Tat (folded substrates)^45–47^ (both described in detail in Section 2.1.2) for export across the cytoplasmic membrane. In addition to these generalized pathways, monoderm bacteria have a distinctive specialized system, the T7SS, although T4SS-like systems have also been reported.^557^

The 11 secretion systems employ fundamentally distinct mechanisms to move substrates across the bacterial cell envelope. These range from ATP-driven threading of unfolded polypeptides through dedicated trans-envelope channels to proton motive force-coupled translocation of folded proteins to the spring-loaded mechanical firing of contractile nanomachines that puncture neighboring cells. Despite this diversity, recurring mechanistic themes emerge: the coupling of nucleotide hydrolysis or membrane potential to directional substrate movement, the use of periplasmic or cytosolic chaperones to maintain substrates in a secretion-competent state, and the dynamic assembly of multi-component complexes that open only transiently to maintain envelope integrity. The following subsections describe the architectures of each secretion system and our current understanding of the secretion mechanism, highlighting both the principles shared across systems and the features that make each system mechanistically unique.

### 10.1 Type 1 Secretion System

The type 1 secretion system (T1SS) is a broadly distributed pathway in diderm bacteria that mediates the direct, one-step translocation of substrates from the cytoplasm to the extracellular environment, bypassing the periplasm entirely.^638–640^ The core architecture spans both membranes and consists of three principal elements: an IM ATP-binding cassette (ABC) transporter that provides energy and substrate specificity, a periplasm-spanning adaptor that bridges the two membranes, and an OM exit duct that forms a continuous channel to the cell surface.^641–643^ These components assemble into a trans-envelope conduit only upon substrate engagement, creating a contiguous path that shields the translocating polypeptide from the periplasmic space.^639,641,644–646^ Substrates are typically large, multidomain proteins such as toxins, proteases, lipases, adhesins, and surface-layer proteins, many of which are associated with virulence or environmental adaptation.^638^ Unlike Sec and Tat substrates, T1SS substrates generally carry noncleaved C-terminal secretion signals,^647,648^ and they are maintained in an unfolded or partially unfolded state prior to export.^639,641,644–646^ The system was first characterized in the late 1980s and early 1990s through studies of toxin secretion,^647–649^ and high-resolution structures of individual components and assembled subcomplexes have since been determined by X-ray crystallography and cryo-electron microscopy, revealing a modular yet tightly coordinated architecture capable of long-range conformational coupling across the cell envelope.^650,651^

Mechanistically, T1SS transport is powered by ATP hydrolysis at the cytosolic face of the inner membrane transporter, which drives substrate engagement and processive translocation through the channel.^644,650–652^ Substrate binding induces conformational changes that promote the recruitment and docking of the periplasmic adaptor and outer membrane duct, forming a sealed trans-envelope channel. Translocation proceeds in a vectorial manner, with the polypeptide threaded through the conduit in a largely extended conformation, and folding is thought to occur predominantly after emergence at the cell surface.^639,645,653^ Unlike Sec and Tat, which rely on electrochemical gradients and/or multiple energy inputs, T1SS appears to depend primarily on ATP-driven conformational cycling, although contributions from substrate folding outside the cell may help prevent backsliding and enhance directionality.^646,651,653,654^ Several mechanistic questions remain unresolved, including how substrate recognition is achieved with high specificity given the diversity of exported proteins, how assembly of the trans-envelope complex is temporally coordinated, and how channel opening is regulated to prevent leakage across the envelope.^641,651,655–657^ In comparison to other secretion systems, T1SS is distinguished by its one-step, periplasm-independent mechanism and its reliance on a tripartite channel that transiently bridges both membranes, contrasting with systems that operate sequentially across individual membranes or that require periplasmic intermediates.

#### 10.1.1 Type 2 Secretion System

The type 2 secretion system (T2SS) is a multiprotein apparatus in diderm bacteria that mediates the two-step export of folded proteins from the periplasm to the extracellular environment (Figure 11).^658–660^ In contrast to T1SS, substrates first traverse the IM via the Sec or Tat pathways, where they fold and often acquire cofactors before being recognized by T2SS.^661,662^ The T2SS architecture spans the entire cell envelope and includes an OM secretin protein that forms a gated exit pore, a periplasmic pseudopilus composed of pilin-like subunits, an IM platform that organizes assembly, and a cytosolic ATPase that drives pseudopilus dynamics.^663,664^ These components are arranged into a coordinated machine in which the pseudopilus extends from the IM toward the OM pore.^664–666^ Substrates include a wide range of enzymes and virulence factors, such as proteases, lipases, toxins, phosphatases, and degradative enzymes, many of which function in nutrient acquisition or host interactions.^663,667^ The system was elucidated in the 1990s through genetic and biochemical studies of extracellular enzyme secretion.^668^ Structural characterization by cryoelectron microscopy, crystallography, and electron tomography has revealed a modular assembly with distinct inner and OM subcomplexes connected across the periplasm.^661,662,669–671^

The T2SS operates through a piston-like model in which cycles of ATP hydrolysis drive the extension and retraction of the pseudopilus, physically pushing folded substrates through the OM secretin.^658^ Substrate recognition is thought to occur in the periplasm, potentially involving structural features that become available after the substrate folds in the periplasm rather than simple linear sequence motifs, although the precise determinants remain incompletely defined.^666,672–674^ Following secretion, disassembly or resetting of the pseudopilus prepares the system for subsequent cycles. Despite substantial advances, key questions remain unresolved, including how substrates are selectively recognized among the diverse pool of periplasmic proteins, how the pseudopilus length and force generation are regulated, and how coordination between inner and OM components is achieved over the periplasmic distance.^661,664,666,672,674,675^ In comparison to other secretion systems, T2SS is distinguished by its reliance on prior Sec/Tat-mediated export and periplasmic folding of secretion substrates as well as its use of a dynamic filamentous structure to drive secretion.

#### 10.1.2 Type 3 Secretion System

Virulence-associated type 3 secretion systems (T3SSs) are specialized, syringe-like nanomachines in diderm bacteria that mediate the direct translocation of effector proteins from the bacterial cytoplasm into eukaryotic host cells (Figure 11).^676–681^ Its architecture spans both bacterial membranes and extends outward as a needle-like projection that can dock onto host membranes.^682–687^ The basal structure consists of a multiring scaffold embedded in the inner and OMs, connected to a central export conduit.^688–690^ On the cytosolic side, a sorting platform has been proposed to coordinate substrate recognition and delivery, while an associated ATPase complex facilitates substrate processing and engagement.^691–694^ Extending from the OM is a rigid needle filament, which terminates in a tip complex that mediates host cell contact and assembly of a translocon pore within the host membrane, thereby completing a continuous channel from the bacterial cytoplasm into the host cytosol.^687,695^ Substrates include effector proteins that manipulate host cell signaling pathways, as well as structural components required for system assembly and function.^696^ These effectors typically lack cleavable signal peptides and are instead recognized through N-terminal secretion signals and chaperone-mediated targeting mechanisms. The T3SS was first identified in the 1990s through studies of bacterial pathogenesis,^697,698^ and its structural organization has been resolved in remarkable detail by cryo-electron microscopy and cryo-electron tomography, revealing near-atomic models of the basal body, needle, and associated cytosolic components.^682–687,695^

The T3SS operates as a highly coordinated protein export machine that couples ATP hydrolysis and the proton motive force to drive substrate unfolding and translocation through a narrow (∼2–3 nm) channel.^694,699,700^ Substrates are maintained in an unfolded or partially unfolded state, often through interactions with specific chaperones that guide them to the secretion apparatus.^696,701–703^ Upon engagement, substrates are threaded through the export conduit in a process that involves sequential unfolding and linearization, followed by rapid passage through the needle into the host cell.^704,705^ The ATPase is proposed to function in substrate recognition, chaperone release, and initial unfolding,^691,692^ while the proton motive force contributes to sustained translocation, although the precise division of labor between these energy sources remains an active area of investigation.^694,700,706,707^ Additional dynamic processes include hierarchical substrate switching during assembly (from structural components to translocators to effectors),^708–710^ gating of the export apparatus,^711,712^ and dynamic turnover of T3SS subunits or complexes thereof.^713–716^ Outstanding questions include how substrates are selectively prioritized, which path(s) the substrates take prior to being threaded into the needle, and how energy transduction is coordinated across the multi-component needle complex.^677–680^ In comparison to other secretion systems, T3SS is distinguished by its ability to inject proteins directly into host cells through a continuous trans-kingdom conduit, its reliance on a needle-like delivery apparatus, and its central role in bacterial virulence, setting it apart from systems that primarily mediate extracellular secretion or periplasmic export.

#### 10.1.3 Type 4 Secretion System

The Type 4 secretion system (T4SS) is a versatile trans-envelope machine found in both diderm and monoderm bacteria that mediates the transfer of macromolecules, including proteins and nucleoprotein complexes, across cellular membranes (Figure 11).^717–720^ In diderm bacteria, the system spans the IM and OM and often extends outward as a pilus-like appendage that can contact recipient cells. High-resolution structures revealed large, multi-layered assemblies with pronounced architectural complexity.^559,721–724^ The core architecture consists of an IM assembly platform containing multiple ATPases, a periplasm-spanning scaffold, and an OM pore complex that together form a continuous conduit. Many Type 4 systems also contain an extracellular pilus or filament that facilitates cell–cell contact and exchange of genetic material.^725,726^ Substrates therefore include DNA–protein complexes involved in conjugation, effector proteins delivered into host cells, and enzymes or toxins associated with intercellular interactions.^727^ Substrate targeting is frequently mediated by C-terminal or internal secretion signals, often in conjunction with adaptor proteins that couple substrates to the secretion machinery.^727–729^ The T4SS was first characterized in the context of bacterial conjugation in the mid-20th century, and subsequent work has revealed its broader role in horizontal gene transfer and pathogenesis.^559,727,730,731^

Mechanistically, T4SS function is driven by ATP hydrolysis at multiple cytosolic ATPase sites, which coordinate substrate processing, channel opening, and, in some systems, pilus biogenesis.^732–734^ Substrate transfer is initiated by recruitment to the IM platform, where conformational changes associated with ATP binding and hydrolysis are thought to promote engagement with the translocation channel.^735–737^ Secretion of DNA substrates is often coupled to relaxase-mediated processing, whereas protein substrates are translocated in a manner that may involve partial unfolding and threading through the conduit.^725,732,738^ In systems that form pili, cycles of extension and retraction may contribute to establishing contact with recipient cells and facilitating transfer, although the relationship between pilus dynamics and substrate movement remains incompletely defined.^720,729^ Despite extensive structural and biochemical characterization, key questions remain, including how T4SSs assemble *in vivo*,^720,722^ how substrates of different classes are selectively recognized and which assembly state(s) facilitate their secretion,^723,739^ how substrates traverse the cell envelope,^720,722^ how energy transduction is coordinated across multiple ATPases,^720^ and how channel gating is regulated to maintain envelope integrity.^720,722,724,734,735^ In comparison to other secretion systems, T4SS is distinguished by its ability to transport both proteins and DNA, its frequent role in cell-to-cell transfer, and its evolutionary relationship to conjugative machinery, setting it apart from systems that are dedicated solely to protein export or host cell intoxication.

#### 10.1.4 Type 5 Secretion System

The Type 5 secretion system (T5SS) is a widespread pathway in diderm bacteria that mediates the export of proteins across the OM following prior translocation into the periplasm via the Sec system (Figure 11).^740–742^ This pathway has historically been termed the autotransporter pathway^743,744^ although this designation is mechanistically misleading for several T5SS subfamilies because passenger translocation is not autonomous. In classical Type Va autotransporters, the passenger and a C-terminal β-barrel domain are encoded within the same polypeptide; however, the β-barrel assembly machinery (BAM) participates directly in secretion. During assembly, the nascent substrate β-barrel engages the lateral gate of BamA to form a transient BamA–substrate hybrid barrel, and experimental mapping of a translocation intermediate demonstrated that the passenger traverses the lumen of BamA rather than the lumen of the fully assembled substrate β-barrel.^279^ Type Vc trimeric autotransporters likewise depend on BAM for assembly and surface exposure, although three substrate polypeptides first associate to form a partially folded trimeric β-barrel intermediate in the periplasm before OM insertion and passenger translocation.^268,745^ In contrast, Type Vb two-partner secretion systems contain a separately encoded Omp85-family transporter whose mature β-barrel constitutes the dedicated OM translocation channel for its cognate passenger protein. Type Vd proteins combine an Omp85-family β-barrel with an N-terminal effector domain, but recent structural work indicates that this subfamily may differ substantially from classical autotransporters, and the extent to which its effector domains undergo extracellular translocation remains unresolved.^746^ The Type Ve subfamily, also known as inverse autotransporters, represents an important pathogenic variant in which the topology is inverted relative to classical autotransporters: the β-barrel translocation unit is N-terminal and the passenger domain is C-terminal and surface-exposed; canonical examples include intimin from enteropathogenic *E. coli* and invasin from *Yersinia* species, which are discussed in the context of OM adhesins in Section 6.3.1.^347^ Substrates are typically adhesins, proteases, toxins, and surface-exposed virulence factors, many of which function in host interaction, immune evasion, or biofilm formation.^741,744^ The system was first described in the late 1980s and early 1990s through studies of secreted virulence proteins,^747^ and structural insights from X-ray crystallography and cryo-electron microscopy have revealed the architecture of both β-barrel domains and assembled complexes.^741,744,748–750^

T5SS operates without direct consumption of ATP or coupling to the proton motive force at the OM. In Type Va autotransporters, Sec-dependent delivery to the periplasm is followed by engagement of the passenger-bearing precursor with BAM. The substrate β-barrel forms a transient hybrid barrel with BamA, while the passenger enters and traverses the BamA lumen in a largely extended conformation. Passenger translocation proceeds from the C-terminus toward the N-terminus and is coupled to progressive folding on the extracellular side, providing a thermodynamic and kinetic bias that favors vectorial export and prevents backward movement.^751–753^ In the Type Ve inverse autotransporters intimin and invasin, the same thermodynamic principles presumably apply, but in reverse orientation: the C-terminal passenger domain folds progressively on the extracellular face to drive translocation through the N-terminal β-barrel. Of note, however, the passenger domains of inverse autotransporters are structurally different from those of classical autotransporters, and it remains unclear how the passenger domains of inverse autotransporters fold progressively to enable secretion. Despite extensive study, several important mechanistic questions remain unresolved, including how passenger engagement and release are coordinated with formation and resolution of the transient BamA–substrate hybrid barrel and how extracellular folding is kinetically coupled to vectorial translocation.^745,754^ In comparison to other secretion systems, the T5SS is distinguished by its minimalist architecture and reliance on periplasmic intermediates, as well as its lack of a dedicated energy source at the OM, contrasting with ATP- or proton motive force-driven systems that actively power substrate export.^755^

#### 10.1.5 Type 6 Secretion System

The Type 6 secretion system (T6SS) is a contractile, phage tail-like apparatus in diderm bacteria that mediates the direct delivery of effector proteins into neighboring bacterial or eukaryotic cells (Figure 11).^756–760^ Its architecture spans the cell envelope and comprises a membrane-anchored baseplate complex connected to a contractile sheath that surrounds an inner tube, capped by a sharp spike complex.^761–764^ The baseplate is embedded in the IM via a multicomponent membrane complex, which also provides a docking platform for assembly.^765,766^ The inner tube is built from stacked ring-like protein subunits and terminates in a trimeric spike sharpened by a penetrating tip, while the surrounding sheath forms a spring-loaded contractile structure.^758,767,768^ Upon contraction, the sheath drives the inner tube and spike outward, puncturing target cell membranes.^769^ Substrates include a diverse repertoire of toxic effector proteins, such as cell wall-degrading enzymes, membrane-disrupting toxins, nucleases, and other antibacterial or anti-eukaryotic factors, which are either attached to the spike/tube or loaded into its lumen.^758,759,769,770^ The T6SS was discovered in the mid-2000s through studies of virulence and interbacterial competition,^771–774^ and its structure has been elucidated in detail using cryo-electron microscopy and cryo-electron tomography, revealing striking similarity to contractile bacteriophage tails, which is consistent with their proposed common origin.^758,761–768,775^

The T6SS functions through a rapid contraction event that converts stored mechanical energy into forceful propulsion of the inner tube and the associated effectors. Assembly begins with formation of the baseplate and membrane complex, followed by polymerization of the inner tube and extension of the surrounding sheath in a high-energy, metastable state.^766,776^ Effector proteins are either fused to structural components or non-covalently associated with the tube and spike, ensuring co-delivery during firing.^758,767,777^ Triggering of sheath contraction results in a dramatic conformational change that shortens and thickens the sheath, driving the tube-spike complex outward across the bacterial envelope and into target cells.^768,776^ After contraction, the sheath is disassembled and recycled by a cytoplasmic ATPase, allowing repeated cycles of assembly and firing.^760,768,778,779^ Despite extensive structural and functional characterization, key questions remain, including how assembly and firing is spatially and temporally regulated in response to environmental or competitive cues,^776,780–782^ how diverse effectors are selectively loaded and targeted.^767,783,784^ In comparison to other secretion systems, T6SS is distinguished by its use of a contractile nanomachine that delivers effectors via mechanical force, rather than translocating substrates through a continuously open channel, and by its prominent role in interbacterial competition and microbial community dynamics.

#### 10.1.6 Type 7 Secretion System

The Type 7 secretion system (T7SS) is a specialized export pathway found predominantly in monoderm bacteria with thick, hydrophobic cell envelopes as well as in mycobacteria (Figure 11).^785–788^ The T7SS mediates the translocation of small, helix-rich proteins and associated complexes across the cytoplasmic membrane and the cell wall/mycomembrane.^789^ The core architecture is centered on a multimeric IM complex composed of several transmembrane helices that assemble into a gated export conduit, flanked on the cytosolic side by a multi-subunit ATPase assembly that organizes substrate recruitment and energizes export.^789–793^ In mycobacteria, this membrane complex is further connected to a periplasmic/cell envelope-associated scaffold that spans the thick cell wall, forming a continuous path to the cell surface.^791,792,794^ Substrates are typically small, α-helical proteins (often secreted as heterodimers), virulence factors, membrane-permeabilizing toxins, and proteins involved in nutrient acquisition and metal homeostasis; many are secreted in pairs or complexes that depend on one another for stability and targeting.^795–797^ Five paralogous T7SSs, referred to as ESX-1 through ESX-5, have been identified in mycobacteria, with each exporting distinct substrates relevant to pathogenesis.^786^ The T7SS was first identified in the early 2000s through studies of major virulence determinants in pathogenic mycobacteria,^798^ and structural characterization by cryo-electron microscopy and X-ray crystallography has revealed large, multi-protein assemblies with defined cytosolic, membrane, and periplasmic domains.^786,790–793,795–797,799^

T7SS-mediated export is driven by ATP hydrolysis within the cytosolic ATPase assembly, which undergoes nucleotide-dependent conformational changes that are thought to coordinate substrate recognition, unfolding (when required), and engagement with the membrane-embedded conduit.^790,792,793,796,800,801^ Substrate targeting involves short C-terminal or internal sequence motifs and obligate partner interactions, and in many cases substrates are delivered as pre-formed complexes that must be selectively recognized by the secretion machinery.^797,802–805^ Current models propose that ATP-driven conformational cycling opens and closes the transmembrane channel and may also remodel substrate complexes to facilitate passage through a relatively narrow conduit.^792,794,806^ In mycobacteria, additional factors are thought to mediate transport across the outer, lipid-rich cell envelope, although the precise mechanism by which substrates traverse this barrier remains incompletely understood.^785,807^ Outstanding questions include how substrates are crossing the mycobacterial OM after reaching the periplasm,^808,809^ how different T7SS subtypes achieve specificity for distinct substrate groups,^810^ and how energy transduction is coupled to transport across both the membrane and the extended cell envelope.^795,801,808,809^ In comparison to other secretion systems, T7SS is distinguished by its adaptation to thick, low-permeability cell envelopes, its reliance on ATP-driven cytosolic assemblies rather than proton motive force, and its specialization for small, structured protein substrates that are often secreted as complexes, highlighting a mechanistic niche distinct from both Sec/Tat and the multi-component systems of diderm bacteria.

#### 10.1.7 Type 8 Secretion System

The Type 8 secretion system (T8SS) is a specialized OM export pathway in diderm bacteria that mediates the assembly and secretion of extracellular amyloid fibers, most notably curli (Figure 11).^291,811–813^ It functions as a two-step system: structural subunits are first translocated across the IM via the Sec pathway and accumulate in the periplasm, after which they are exported across the OM through a dedicated β-barrel pore (OM secretin-like channel).^289,290,814^ The core architecture consists of this OM pore-forming β-barrel, a periplasmic chaperone network that prevents premature aggregation, and surface-localized nucleator proteins that template fiber assembly.^571,591,593,594,815,816^ Together, these components form a coordinated system in which secretion is tightly coupled to extracellular polymerization. Substrates are primarily amyloidogenic fiber subunits and associated accessory proteins, which assemble into extracellular fibrils that contribute to surface adhesion and cell-to-cell cohesion in biofilms.^566,811,817^ The pathway was elucidated in the late 1990s and early 2000s through genetic and biochemical studies of curli biogenesis,^569,817,818^ and structural insights from X-ray crystallography, solid-state NMR, and cryo-electron microscopy have revealed both the β-barrel secretion pore and the cross-β architecture of the assembled fibers.^289,290,568,570,814,819^

T8SS operates without direct ATP consumption or coupling to the proton motive force at the OM, instead relying on Sec-dependent delivery to the periplasm and assembly of amyloid fibers at the cell surface to drive secretion. In the periplasm, dedicated chaperones bind subunits and maintain them in a soluble secretion-competent state, preventing premature aggregation. Upon reaching the OM pore, amyloidogenic substrates are translocated in an unfolded or partially structured conformation and are immediately incorporated into growing extracellular fibers through a templated polymerization process, in which a surface-exposed nucleator protein seeds assembly.^571,816,820–822^ This coupling of secretion to polymerization provides a thermodynamic bias that ensures directionality and minimizes backsliding.^291,823^ Despite substantial advances, several key questions remain, including how the secretion pore accommodates substrates with repetitive, aggregation-prone sequences without clogging, how substrate flux is regulated, and how specificity is maintained among multiple amyloid subunits and accessory proteins.^291,568,813,814,824,825^ In comparison to other secretion systems, T8SS is distinguished by its tight coupling of secretion to extracellular self-assembly, its reliance on periplasmic chaperone control rather than cytosolic targeting, and its role in producing functional amyloids rather than soluble effector proteins.

#### 10.1.8 Type 9 Secretion System

The Type 9 secretion system (T9SS) is a specialized protein export pathway found in members of the Bacteroidetes, where it mediates the translocation of proteins from the periplasm across the OM to the cell surface or extracellular milieu (Figure 11).^826–831^ As with several OM secretion systems, T9SS operates downstream of the Sec pathway, which delivers substrates into the periplasm in an unfolded state, after which they fold prior to secretion. The architecture spans the cell envelope and includes an OM translocon that forms a gated secretion pore, a large periplasmic and IM-associated scaffold, and a cytoplasmic/IM energy-transducing complex.^826,832–839^ These components are organized into a coordinated assembly that couples energy input at the IM to secretion at the OM over a considerable periplasmic distance. A hallmark of T9SS substrates is the presence of a conserved C-terminal targeting signal, which directs proteins to the system and is often cleaved or modified during secretion.^840^ Substrates include cell-surface adhesins, gliding motility proteins, proteases, s-layer proteins, polysaccharide-degrading enzymes, and virulence factors, many of which are either retained on the cell surface through covalent attachment to cell envelope components or released extracellularly.^827,828,841^ The system was first delineated in the late 2000s through genetic analyses of gliding motility and surface protein, which identified a conserved set of envelope-associated components required for both processes.^842^ Subsequent structural studies using cryo-electron microscopy, X-ray crystallography, and cryo-electron tomography have revealed that the OM translocon forms a large β-barrel channel with a gated lumen, while IM-associated complexes assemble into motor-like structures that couple the proton motive force to secretion.^826,832–839,827,828,841^

Substrates of the T9SS bearing the C-terminal targeting signal are recognized in the periplasm and delivered to the secretion machinery, where they are translocated through the OM pore.^843,844^ In many cases, secretion is coupled to post-translocation processing events, including cleavage of the targeting signal and covalent attachment of the protein to cell surface glycoconjugates, effectively anchoring the substrate to the OM.^845–848^ The available information supports a model in which T9SS exports and anchors large adhesins to the cell surface, where they are translocated along helical tracks to drive gliding motility.^832,835^ However, the overall organization and full 3D architecture of the complete T9SS and the precise molecular mechanism by which energy derived from the proton motive force is transmitted across the periplasm remain incompletely understood. Additional open questions include how substrate specificity is achieved given the diversity of exported proteins, what is the sequence of interactions and conformational changes during substrate translocation, and how secretion is coordinated with surface attachment and motility functions in response to environmental signals.^827,831^ In comparison to other secretion systems, T9SS is distinguished by its restriction to a specific bacterial phylum, its reliance on a C-terminal targeting signal, and its integration with cell surface remodeling and motility processes, setting it apart from systems that primarily mediate soluble protein secretion or direct injection into target cells.

#### 10.1.9 Type 10 Secretion System

The Type 10 secretion system (T10SS) is a recently described export mechanism in diderm bacteria that mediates the release of proteins from the cytoplasm to the extracellular environment through controlled disruption of the cell envelope (Figure 11).^849–851^ Unlike canonical secretion systems that form dedicated trans-envelope channels, T10SS is composed of IM pore-forming proteins that oligomerize to create large lesions, together with periplasmic or envelope-targeted cell wall–degrading enzymes that locally remodel or cleave the PG layer.^852–854^ In some cases, these activities are coordinated with OM destabilization or vesiculation processes, resulting in the release of cytoplasmic contents without the assembly of a continuous protein-conducting conduit.^855–859^ Substrates include cytoplasmic enzymes, toxins, and other proteins lacking classical secretion signals, many of which are released in response to environmental cues or during specific developmental or stress conditions.^860,861^ The system was identified in the early 2020s through genetic and functional studies of toxin release pathways,^48^ and structural characterization of its components by cryo-electron microscopy and time lapse fluorescence microscopy has revealed oligomeric membrane pores and enzymatic domains consistent with phage-derived lysis modules.^862–864^

T10SS function is driven by the regulated formation of large membrane pores, which permit the passage of proteins that would otherwise be excluded by size or folding state.^865,866^ Activation typically involves controlled oligomerization of the IM pore-forming proteins, leading to rapid permeabilization of the cytoplasmic membrane, followed by enzymatic degradation of the PG layer, thereby relieving mechanical constraints on the envelope.^863,867^ In some systems, this process results in partial or complete cell lysis, while in others, it appears to be more tightly regulated, allowing transient permeability or localized envelope disruption.^849,850,862,868^ Because substrate release does not require unfolding or threading through a narrow channel, T10SS can accommodate fully folded proteins and large macromolecular assemblies.^866,869^ However, this mode of secretion raises important questions regarding how cells regulate how T10SS pores assemble and select cargo, how nonlytic vs lytic outcomes are specified, how diverse the phage-derived modules are, and how broadly T10SSs contribute to bacterial interaction with hosts and environments.^849,850,862,868^ In comparison to other secretion systems, T10SS is distinguished by its reliance on phage-like lytic modules rather than structured secretion machines, its ability to release proteins without a defined trans-envelope conduit, and its frequent coupling to stress responses or programmed cell lysis, highlighting a fundamentally different strategy for protein export.

#### 10.1.10 Type 11 Secretion System

The Type 11 secretion system (T11SS) is a recently defined pathway in diderm bacteria that mediates the export and surface display of lipoproteins across the OM (Figure 11).^870,871^ It functions downstream of the Sec pathway, which delivers substrates to the periplasm and in conjunction with the Lol pathway (“localization of lipoproteins” pathway), which enables substrate processing as lipid-anchored proteins tethered to the inner leaflet of the OM.^872–874^ The T11SS mediates the surface exposure of lipoproteins across the OM.^875,876^ The defining structural feature of T11SS is an OM β-barrel translocon that assembles into a gated pore, often accompanied by periplasmic accessory domains or partner proteins that facilitate substrate engagement.^319,870,875,877^ Together, these proteins are known as the Slam (Surface Lipoprotein Assembly Modulator) family of proteins. T11SS substrates are typically lipoproteins that remain membrane-anchored via lipid modifications while being translocated through the OM to the cell surface.^319,875^ These substrates include surface-exposed adhesins, nutrient acquisition proteins, immune modulators, and host interaction factors, many of which play central roles in colonization and symbiosis.^871,878,879^ The system was delineated in the early 2020s through comparative genomics and functional studies that identified the Slam family as a conserved class of OMPs required for lipoprotein surface exposure. Biochemical and reconstitution experiments demonstrated that Slam proteins function as OM translocons for surface lipoproteins, consistent with predicted β-barrel architectures capable of forming protein-conducting pores.^870,875^

T11SS operates without direct ATP consumption at the OM, instead it is thought to rely on conformational dynamics of the OM translocon to drive export.^319,874^ Substrate lipoproteins are first inserted into the OM inner leaflet, after which they are recognized by the T11SS machinery and guided through the β-barrel pore to the cell surface.^319,877^ Current models suggest that translocation may involve partial unfolding or reorientation of the lipoprotein, followed by refolding or stabilization once exposed extracellularly, although the extent of unfolding required remains unclear.^319^ The energetic basis of this process is not fully resolved and may involve contributions from periplasmic chaperones, membrane insertion energetics, and substrate folding on the cell surface.^880^ Key open questions include how the translocation pore carries out its function,^319,870^ how specificity is achieved among diverse lipoproteins,^871,881^ and how T11SS activity integrates with other pathways for surface exposure of OM lipoproteins and LPS.^880,882–884^ In comparison to other secretion systems, T11SS is distinguished by its specialization for lipoprotein surface exposure, its reliance on OM translocation without a continuous trans-envelope channel, and its integration with lipidation and membrane trafficking pathways, setting it apart from systems that export soluble proteins or inject effectors into target cells.

### 10.2 Role of Secretion in Pathogenesis

Bacterial secretion systems contribute to pathogenesis not through the machinery itself but through the effectors, toxins, and other macromolecules that they deliver. Across the 11 named specialized systems described in Section 10 and 10.1, the pathogenic roles range from direct injection of effector proteins into host cells to release of degradative enzymes into the extracellular environment to dissemination of antibiotic resistance genes through bacterial populations.^885,886^ The systems most directly linked to virulence during infection are T3SS, T4SS, T6SS, and T7SS, each of which delivers substrates that manipulate host cell biology in distinct ways. T1SS, T2SS, and T5SS contribute to pathogenesis primarily by secreting toxins, proteases, and adhesins into the extracellular environment. T8SS contributes through biofilm formation on host surfaces, with curli fiber assembly described in Section 9. T9SS, T10SS, and T11SS have more specialized or restricted roles, which are discussed briefly below.

The T3SS is among the best-characterized virulence delivery systems in diderm pathogens, injecting effector proteins directly into the host cell cytoplasm, where they subvert signaling, cytoskeletal organization, and immune responses.^680,885^ In *S. enterica*, the SPI-1 T3SS injects effectors including that collectively remodel the host actin cytoskeleton to drive bacterial uptake into nonphagocytic cells.^887–889^ A second T3SS encoded by SPI-2 is expressed in later stages of infection and injects effectors that remodel the *Salmonella*-containing vacuole to prevent lysosomal fusion and support intracellular replication.^890^ In *Yersinia* species, the Ysc T3SS injects effectors that collectively disrupt phagocytosis, inhibit inflammatory signaling, and induce macrophage apoptosis, allowing the bacteria to resist clearance in lymphoid tissue.^891^ In enteropathogenic *E. coli*, the LEE-encoded T3SS injects Tir into the host membrane, where it acts as the receptor for the bacterial adhesin intimin (Section 6.2.1), driving intimate attachment and actin pedestal formation beneath the bacterium.^348^

The T4SS contributes to pathogenesis through two distinct mechanisms: direct effector injection into host cells and horizontal transfer of virulence and resistance determinants between bacteria. In *H. pylori*, the Cag pathogenicity island encodes a T4SS that injects the oncoprotein CagA directly into gastric epithelial cells, where it is phosphorylated by host Src and Abl kinases and disrupts cell polarity, promotes proliferation, and inhibits apoptosis, contributing to the progression from chronic gastritis to gastric cancer.^892^ In *L. pneumophila*, the Dot/Icm T4SS injects over 300 effector proteins into the host macrophage that collectively manipulate vesicle trafficking, prevent phagosome maturation, and remodel the *Legionella*-containing vacuole into an endoplasmic reticulum-derived compartment permissive for intracellular replication.^893^ The PG remodeling enzyme PgdA, described in Section 4, is required for proper assembly of the *L. pneumophila* Dot/Icm apparatus, illustrating how cell envelope components directly support secretion system function. T4SS also drives pathogenesis indirectly through conjugative transfer of virulence and antibiotic resistance plasmids, as described in Section 9, contributing to the spread of resistance determinants, including extended-spectrum β-lactamases and carbapenemases, across clinical pathogen populations.^894^

The T6SS delivers its effectors through a contractile phage tail-like apparatus that punctuates target cell membranes directly, as described in Section 10.1.5. In *P. aeruginosa*, T6SS effectors include phospholipases, cell-wall hydrolases, and nucleases that kill competing bacteria in polymicrobial infections and in the chronic lung infection environment of cystic fibrosis, providing a competitive fitness advantage.^895^ The T6SS also has direct antihost roles: *Burkholderia* species deploy T6SS to escape macrophage phagosomes and gain access to the host cell cytoplasm, facilitating intracellular survival and cell-to-cell spread.^896^ *V. cholerae* T6SS delivers effectors that lyse competing bacteria and can damage intestinal epithelial cells during infection.^897^ The competitive interbacterial function of the T6SS is unique and is increasingly recognized as relevant to infection outcomes because it shapes the composition of polymicrobial communities at infection sites, with implications for the severity and persistence of chronic infections.^895^

The T7SS is the primary virulence secretion pathway in mycobacteria, where five paralogous systems ESX-1 through ESX-5 each export distinct substrates relevant to pathogenesis, as introduced in Section 7 and described in the context of the mycobacterial cell envelope in Section 6.2.^786^ ESX-1 secretes the heterodimeric complex EsxA (ESAT-6) and EsxB (CFP-10), which permeabilizes phagosomal and plasma membranes, allowing *M. tuberculosis* to access the host cytoplasm and trigger NLRP3 inflammasome activation and type I interferon responses.^898,899^ ESX-3 is essential for iron and zinc acquisition during infection and is required for *M. tuberculosis* viability within the host, making it a target of interest for antimicrobial development.^900^ ESX-5 is the primary export pathway for the large PE/PPE protein family described in Section 7, many of which modulate TLR2 and TLR4 signaling, suppress inflammatory cytokine production, and facilitate immune evasion during chronic infection.^901^ In some strains of the monoderm pathogen *S. aureus*, T7SS secretes the effector EsaD, a nuclease toxin, and a companion antitoxin, EsaG.^902^

T1SS, T2SS, and T5SS contribute to pathogenesis primarily through extracellular secretion of toxins and degradative enzymes rather than direct host cell injection. T1SS secretes large, unfolded substrates including the *E. coli* pore-forming hemolysin HlyA, which lyses erythrocytes and epithelial cells to release nutrients during urinary tract infection,^639^ and *Serratia marcescens* lipase and hemolysin, which degrade host cell membranes and contribute to opportunistic infection.^903^ T2SS secretes folded enzymes of broad virulence relevance, including cholera toxin in *V. cholerae*, which ADP-ribosylates the Gs α subunit to drive constitutive adenylate cyclase activity and the secretory diarrhea that defines cholera,^904,905^ and exotoxin A in *P. aeruginosa*, which ADP-ribosylates elongation factor 2 to halt host cell protein synthesis.^906^ T5SS autotransporters and two-partner secretion systems export surface-exposed and secreted virulence factors including the serine protease autotransporters of *Enterobacteriaceae* such as EspP and Pet, which cleave host complement proteins, coagulation factors, and mucosal barrier components to promote colonization,^907^ and the trimeric adhesins YadA and NadA discussed in Section 6.2.1.^341,342^ T8SS contributes to pathogenesis through curli fiber assembly, which promotes biofilm formation on host tissues and implanted devices, and through curli-induced co-activation of TLR2 and TLR1 that can drive systemic inflammatory responses during bacteremia.^908^ T9SS, expressed in Bacteroidetes including the periodontal pathogen *P. gingivalis*, secretes gingipain cysteine proteases that degrade host immunoglobulins, complement components, and connective tissue matrix proteins, contributing to the tissue destruction characteristic of severe periodontitis.^909^ T10SS and T11SS have pathogenic roles that remain less fully characterized; T11SS in *Neisseria* species mediates the surface display of lipoproteins including the transferrin-binding protein TbpB described in Section 5.3, contributing to iron acquisition and immune evasion during invasive infection.^318,360,909^

### 10.3 Secretion System Therapeutic Targets

The 11 classified secretion systems (T1SS–T11SS) all share one defining therapeutic property: they are required for the delivery of virulence factors and are therefore attractive anti-virulence targets whose inhibition would disarm the pathogen. Because secretion system components are absent from mammalian cells and are surface-exposed or periplasm-accessible, they are also exploitable as vaccine antigens. Therapeutic development has concentrated overwhelmingly on T3SS, T4SS, and the mycobacterium-specific T7SS (ESX systems), where the molecular architecture and pathogenic mechanisms are best characterized, while T1SS, T2SS, T5SS–T9SS, T10SS, and T11SS remain at the target validation stage with no clinical programs specifically targeting the secretion machinery.^910^

The type III secretion system is the most extensively pursued antivirulence target among the secretion systems. Multiple small-molecule inhibitor classes have been identified that disrupt T3SS assembly, needle polymerization, or effector translocation *in vitro* and in animal models of infection. However, none have advanced to clinical trials, in part because T3SS is dispensable for bacterial growth, raising concerns about the stringency of selective pressure and the achievable therapeutic window.^910^ Anti-PcrV monoclonal antibodies have a well-established preclinical track record: the foundational murine anti-PcrV antibody MAb166 demonstrated that blocking PcrV prevents T3SS toxin delivery and protects against lethal *P. aeruginosa* lung infection.^911^ And, combination of anti-PcrV antibodies with antibiotics provides a synergistic survival benefit beyond either alone.^912^ The bispecific antibody MEDI3902 (MedImmune/AstraZeneca), targeting both PcrV and the Psl exopolysaccharide, provided dose-dependent protection in both murine and rabbit pneumonia models and entered phase 2 clinical trials.^913^ The more recently characterized humanized antibody COT-143 (INFEX702, Shionogi) demonstrates the same mechanism with additional evidence of neutrophil protection and facilitated phagocytosis confirmed by the crystal structure of the antibody-PcrV complex.^914^ AAV-vectored delivery of anti-PcrV antibodies has also been explored as a long-term prophylactic strategy for high-risk populations including those with cystic fibrosis.^915^

The type IV secretion system, exemplified by the *H. pylori* Cag pathogenicity island apparatus, translocates the oncoprotein CagA directly into gastric epithelial cells, where it triggers signaling cascades associated with gastric carcinogenesis. The T4SS complex is therefore a validated virulence target, and several small-molecule inhibitors of T4SS ATPase activity and assembly have been identified in preclinical studies, although none are in clinical development.^916^ In mycobacteria, the ESX-1 T7SS secretes proteins that permeabilize the phagosomal membrane, enabling *M. tuberculosis* to escape into the cytoplasm and prevent phagolysosomal maturation. ESX-1 is essential for full virulence and is absent from the BCG vaccine strain, identifying it as a high-priority antivirulence target. Virtual screening campaigns and cell infection models have identified candidate ESX-1 inhibitors, although the field remains at the early preclinical stage.^917^

#### 10.3.1 Secretion System Vaccine Targets

Secretion system structural proteins are immunogenic during natural infection and are vaccine antigens, most notably in the context of *Shigella* and *P. aeruginosa*. In *Shigella*, the T3SS needle tip protein IpaD and the translocon protein IpaB are surface exposed during infection, highly conserved across *Shigella* serotypes, and elicit protective immune responses in animal models. As conserved structural components independent of serotype, they are particularly attractive for serotype-independent protection, addressing one of the fundamental limitations of polysaccharide-based *Shigella* vaccines.^918^ Despite more than a century of research, no globally licensed *Shigella* vaccine has been developed. Multiple candidates have progressed to phase 2 and phase 3 clinical trials, including live attenuated strains, conjugate polysaccharide formulations, and protein subunit approaches incorporating IpaB, IpaD, and other T3SS-associated proteins. The T3SS component PcrV of *P. aeruginosa* is immunogenic in infected patients and is included as an antigen in multicomponent *P. aeruginosa* vaccine formulations under investigation. However, the most clinically advanced anti-PcrV program, the bispecific antibody MEDI3902 targeting both PcrV and the Psl exopolysaccharide, did not meet its primary end point in the Phase 2 trial. This disappointing result emphasizes that surface exposure and preclinical efficacy do not guarantee clinical translation for T3SS-targeted approaches.^919^ In *M. tuberculosis*, the ESX-1 substrates, ESAT-6 and CFP-10, are among the most immunodominant T cell antigens in natural infection and are included in multiple next-generation subunit TB vaccine formulations, most notably M72/AS01E. The M72/AS01E Phase 3 trial initiated first doses in March 2024 and continues to be followed.^425^ Across T1SS through T11SS other than T3SS, T4SS, and T7SS, the structural components have not yielded clinical vaccine candidates because the apparatus components are generally not surface-exposed in a manner accessible to antibody-mediated neutralization.^910^

## 11 Summary and Future Directions

The bacterial surface is one of the most chemically and architecturally diverse interfaces in biological world. As this Review has described, the layers that constitute this surface, from the IM outward through the PG, teichoic acids, S-layer, OM, mycomembrane, capsule, and surface appendages, each represent distinct evolutionary solutions to the shared challenge of maintaining cellular integrity while navigating a hostile host environment. The remarkable variation in lipid composition, protein architecture, carbohydrate modification, and secretion machinery documented across the *Gracilicutes* and *Terrabacteria* clades underscores how deeply membrane chemistry is intertwined with the mechanisms of infection, from initial adhesion and colonization to immune evasion and persistence.

Several themes emerge from the survey of this diversity. First, layering and redundancy are recurring organizational principles: the cell envelope is not a single barrier but a series of functionally distinct compartments, each with dedicated biogenesis machinery and each contributing independently to pathogenesis. Second, chemical modifications are a universal adaptation strategy. Whether through lipid A remodeling to attenuate TLR4 activation, d-alanylation of teichoic acids to resist antimicrobial peptides, O-acetylation of CPS to alter antigenicity, or antigenic variation of S-layer proteins to evade adaptive immunity, bacteria continuously tune the chemistry of their surfaces in response to host pressures. Third, biogenesis and pathogenicity are inseparable: the machinery that builds the surface (the BAM complex, the Lol pathway, the sortases, the secretion systems, and the chaperone networks) is simultaneously the machinery that equips it for infection. Understanding one therefore informs the other.

These insights have direct therapeutic implications. The components described in this Review represent a rich landscape of potential antimicrobial targets, many of which are either absent from or structurally distinct from their eukaryotic counterparts. The LPS transport machinery, sortase-mediated surface protein display, OMP biogenesis via the BAM complex, lipoprotein trafficking through the Lol pathway, capsule biosynthetic enzymes, pilus assembly systems, and type III through type VII secretion systems have all been explored as targets for small molecules, biologics, or vaccine antigens with varying degrees of preclinical and clinical success. Capsular polysaccharide conjugate vaccines against *H. influenzae*, *S. pneumoniae*, and *N. meningitidis* stand as proof of concept that a deep mechanistic understanding of surface architecture can be translated into transformative public health interventions.

Significant gaps remain. The biogenesis pathways for the integral proteins of the mycomembrane have not been identified. The mechanisms by which many surface lipoproteins are flipped to the cell exterior remain poorly understood. The full structural diversity of BAM complexes across bacterial phyla, as recently highlighted by divergent architectures in Bacteroidota and spirochetes, is just beginning to be appreciated. The regulation of capsule expression in response to host signals, the molecular basis of curli-mediated innate immune activation, and the precise mechanisms by which type IX and type X secretion systems couple energy to substrate export are areas of active investigation where much remains to be learned. In addition, the contributions of surface structures to polymicrobial infections, biofilm communities, and interbacterial competition represent an expanding frontier that the field is just beginning to address systematically.

As antibiotic resistance continues to erode the efficacy of existing therapeutics, a deeper molecular understanding of bacterial surface diversity will be essential for the development of next-generation interventions. The chemical determinants of surface assembly, stability, and host recognition described throughout this Review provide a framework for that effort, and the breadth of mechanisms now recognized across bacterial lineages ensures that this remains one of the most fertile areas of inquiry in microbiology and chemical biology.

## Abbreviations

ABC: ATP-binding cassette
ACP: acyl carrier protein
AGM: arabinogalactan mycolates
ATP: adenosine triphosphate
BAM: β-barrel assembly machinery
BCE: B-cell epitope
BcsA/B/C: bacterial cellulose synthase subunits A, B, C
CBP: choline-binding protein
CCL2: C–C motif chemokine ligand 2
CCR: C–C chemokine receptor
CD: cluster of differentiation
CDP: cytidine diphosphate
CEACAM: carcinoembryonic antigen-related cell adhesion molecule
CL: cardiolipin
CPS: capsular polysaccharide
CsgA/B/C/E/F/G: curli-specific gene proteins (curli fiber assembly components)
CU: chaperone-usher (pili assembly pathway)
CWSS: cell wall sorting signal
DAG: diacylglycerol
DC-SIGN: dendritic cell-specific ICAM-3-grabbing non-integrin
DegP: periplasmic serine endoprotease/dual-function chaperone
DNA: deoxyribonucleic acid
EPEC: enteropathogenic Escherichia coli
ESX: ESAT-6 secretion system (Type VII secretion system)
FabB/D/F/H/I: fatty acid synthesis enzymes (FASII pathway)
FASII: type II fatty acid synthesis pathway
FH: Factor H (complement regulator)
FkpA: FK506-binding protein A (periplasmic chaperone/PPIase)
FtsH: filamentous temperature-sensitive protease H (AAA+ membrane protease)
G3P: glycerol-3-phosphate
GroP: glycerophosphate (LTA backbone unit)
GT/GTase: glycosyltransferase
IM: inner membrane
IMP: inner membrane protein
KDO: 3-deoxy-D-manno-oct-2-ulosonic acid (keto-deoxyoctulosonate)
KpsM*T*/KpsD/KpsE: ABC transporter-dependent CPS export components
LC3: microtubule-associated protein 1*A*/1B light chain 3 (autophagy marker)
LCP: LytR-CpsA-Psr enzyme (transfers SCWP to PG)
Lgt: prolipoprotein diacylglyceryl transferase
Lit: lipoprotein intramolecular transacylase
Lnt: apolipoprotein N-acyltransferase
LolA/B/C/D/E: lipoprotein OM localization components A–E
LOS: lipooligosaccharide
LPS: lipopolysaccharide
LpqY-SugABC: sugar uptake ABC transporter system (mycobacteria)
LspA: lipoprotein signal peptidase II
LTA: lipoteichoic acid
LtaA/S: LTA flippase (LtaA)
(LtaS): LTA synthase
mAGP: mycolyl-arabinogalactan-PG complex
MAPK: mitogen-activated protein kinase
MFP: membrane fusion protein (T1SS component)
Mla: maintenance of lipid asymmetry pathway
MmpL3: mycobacterial membrane protein large 3 (TMM transporter)
MOM: mycobacterial OM
MP: membrane protein
MSCRAMM: microbial surface components recognizing adhesive matrix molecules
MurA–MurG/MurJ: PG biosynthesis enzymes
NAG/GlcNAc: N-acetylglucosamine
NAM/MurNAc: N-acetylmuramic acid
NF-κB: nuclear factor kappa-light-chain-enhancer of activated B cells
NMR: nuclear magnetic resonance
NOD1/NOD2: nucleotide-binding oligomerization domain proteins 1 and 2 (pattern recognition receptors)
OM: OM
OMP: OM protein
OMV: OM vesicle
PA: phosphatidic acid
PC: phosphatidylcholine
PDIM: phthiocerol dimycocerosate
PE: phosphatidylethanolamine; also PE protein family (proline-glutamate) in mycobacteria
PE_PGRS: PE proteins with polymorphic GC-rich repetitive sequence domain
PG: phosphatidylglycerol; also peptidoglycan (context-dependent)
PGT: polyprenol phosphate phosphoglycosyltransferase
PI: phosphatidylinositol
PlsB/C/X/Y: acyltransferases for phosphatidic acid biosynthesis
POTRA: polypeptide transport-associated (domain of BamA)
PPE: PPE protein family (proline–proline-glutamate) in mycobacteria
PS: phosphatidylserine
SCWP: secondary cell wall polysaccharide
SecA/B/D/E/F/G/Y: components of the Sec translocon machinery
SecA2/SecY2: accessory Sec secretion system (subset of monoderm organisms)
SLH: surface layer homology (domain)
SLP: surface-layer protein
Skp: 17 kDa protein (periplasmic chaperone)
SP: signal peptide
SRP: signal recognition particle
SrtA–F: sortase enzymes A through F
SurA: periplasmic chaperone/peptidylprolyl isomerase
T1SS–T11SS: Type I through Type XI secretion systems
TarO/A/B/F/L/M/S/GH/TUV: wall teichoic acid biosynthesis enzymes in S. aureus
TDM: trehalose dimycolate (cord factor)
TLR: Toll-like receptor
TMM: trehalose monomycolate
TNF: tumor necrosis factor
TPR: tetratricopeptide repeat
TRIF: TIR-domain-containing adapter-inducing interferon-β
UDP: uridine diphosphate
Und-P: undecaprenyl phosphate (lipid carrier)
WTA: wall teichoic acid
Wzc/Wza/Wzi/Wzx/Wzy: Wz-family CPS biosynthesis and export proteins
YidC: inner membrane insertase and chaperone
YpfP: glycolipid anchor synthase (LTA biosynthesis)
